# Photoinduced movement: how photoirradiation induced the movements of matter

**DOI:** 10.1080/14686996.2022.2142955

**Published:** 2022-11-30

**Authors:** Tetsuo Yamaguchi, Makoto Ogawa

**Affiliations:** a Department of Energy and Materials Engineering, Dongguk University-Seoul, Seoul, South Korea; b School of Energy Science and Engineering, Vidyasirimedhi Institute of Science and Technology (VISTEC), Rayong, Thailand

**Keywords:** Photochromism, photocatalysis, photoinduced movement, interface, patterning

## Abstract

Pioneered by the success on active transport of ions across membranes in 1980 using the regulation of the binding properties of crown ethers with covalently linked photoisomerizable units, extensive studies on the movements by using varied interactions between moving objects and environments have been reported. Photoinduced movements of various objects ranging from molecules, polymers to microscopic particles were discussed from the aspects of the driving for the movements, materials design to achieve the movements and systems design to see and to utilize the movements are summarized in this review.

## GRAPHICAL ABSTRACT

**Figure:**
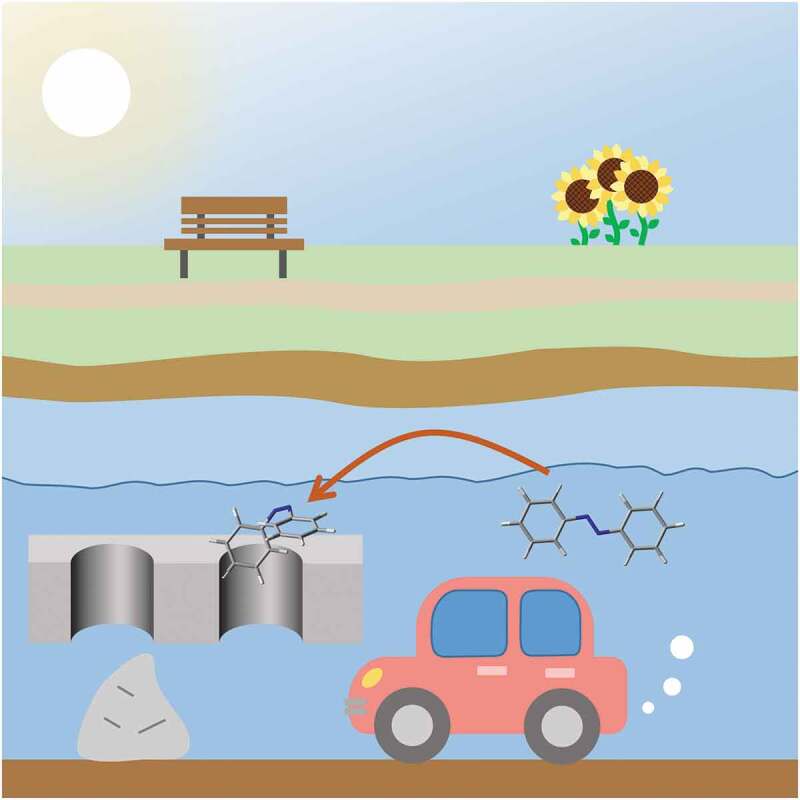

## 1. Introduction

Photoirradiation induces many movements in nature in different size scales. In geosphere, ocean currents originate from the differences in sun light irradiation, which is stronger at the equator and weaker at the polar regions. In biosphere, phototaxis, which is an ability of organisms to move toward or away from light, is seen in different size scales, ranging from bacteria and protists to invertebrates such as insects and jelly fish [1,2]. In a smaller world, photosynthesis is a photoinduced phenomenon, in which chemical reactions and movement of reactant and product molecules occur simultaneously under sun light [3]. Many more macroscopic phenomena in biological and geological systems are initiated by photochemical, photothermal and photobiochemical events at the molecular level [4].

As the above mentioned phenomena are induced by the molecular reactions, chemical systems that show movements of matter have been called as smart materials (photoresponsive materials) [5,6]. One of the key developments in the photoinduced movement in artificial systems had been reported by Shinkai et al. in the 1980s, who demonstrated the regulation of the binding properties of crown ethers by covalently linked photoisomerizable azobenzene units to drive active transport of ions across liquid membranes and membrane mimetic systems by photoirradiation [7]. Since then, set-up to follow the movement, molecules to act as receptor, and the moving objects have been designed from various expertise extensively. The range of the objects that can be moved by irradiation has been extended from molecule/ion to macrosocpic bodies, which is followed directly using an optical microscope and naked eyes. As an extension, photoinduced motion of macromolecules in micrometer scale has been used to create grating (surface relief grating) for optical application. In some systems, Marangoni flow, which was induced by photochemical and photothermal reaction, was thought to play the movement of objects; it can be regarded as ‘nano’ ocean current! In this review article, molecular design, supramolecular design and system design to show photoinduced movements of various objects from nano- to micro-meter scales will be introduced. Photochromic molecules [8] have been used to convert light to mechanical energy using photoinduced changes (photoinduced molecular structure changes, photocatalytic reactions, photoinduced hydrophilicity and photothermal effects).

## 2. Photochromic units used in the studies introduced in this review

**Scheme 1.**
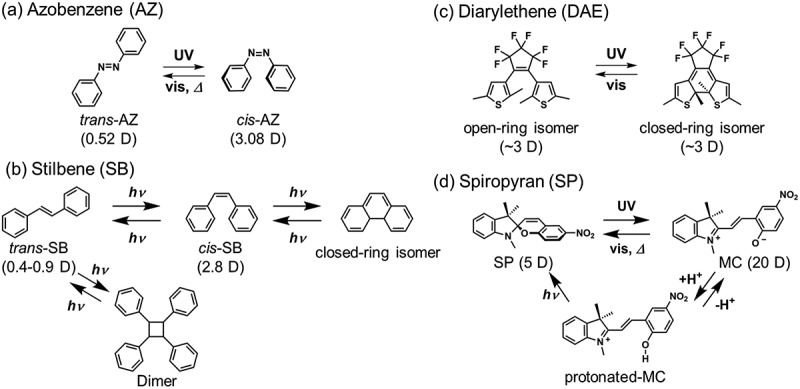
Photochromism of (a) azobenzene (AZ), (b) stilbene (SB), (c) diarylethene (DAE) and (d) spiropyran (SP), where values in brackets are dipole moments of the photochromic compounds [9–13].

As photochromic molecules [8] have been used to convert light to mechanical energy, photochromic molecules introduced in the present review articles are introduced here. There are several photochromic species and, among them, azobenzene (abbreviated as AZ), stilbene (abbreviated as SB), diarylethene (abbreviated as DAE), spiropyran (abbreviated as SP) and spirooxazine (abbreviated as SO) have commonly been used to design photoresponsive systems. The changes in the molecular geometry and molecular polarity are shown in Scheme 1. AZ shows photoisomerization from *trans*- to *cis*-isomers by UV irradiation as shown in Scheme 1(a). Metastable *cis*-isomer returns to thermodynamically stable *trans*-isomer by visible light irradiation or heat. The molecular structure change of AZ accompanied change in dipole moment (*trans*-isomer: 0.52 D and *cis*-isomer: 3.08 D) [9,10]. *trans*-SB also shows *trans-cis* isomerization as shown in Scheme 1(b). The *cis*-isomer of SB shows both the isomerization to the *trans*-isomer and a ring closing reaction. The dipole moments of the *trans*- and *cis*-isomers were reported as 0.4–0.9 D and 2.8 D, respectively [11]. SB shows [2 + 2] photodimerization following Woodward-Hoffmann rules when two SB molecules are close each other. The substitution of the ethylene moiety of *cis*-SB suppressed the *trans-cis* isomerization and the dimerization of SB, where the substituted derivatives are called diarylethene (DAE). DAE shows reversible ring-closing and ring-opening reactions by UV and visible light irradiation, respectively, as shown in Scheme 1(c) [14]. The isomerization does not occur by heat because both of the isomers are thermodynamically stable, so that one can expect real photochromic systems. The dipole moments of the two isomers of DAE were almost same, while the difference was controllable by the substituents up to 3 D [12]. SP transforms to zwitter ionic form called as merocyanine (MC) or photomerocyanine by UV irradiation as shown in Scheme 1(d). The electronically neutralized SP has smaller dipole moment (5 D) than that (20 D) of the zwitter ionic MC [13]. SP is the stable isomer in non-polar environment to show photochromism. On the other hand, MC is stabilized in polar environment to show negative photochromism [15,16], which is the opposite reaction of conventional photochromism from colorless SP to colored MC. In order to control the photochromic reactions from the aspects of reaction yield, kinetics, selectivity, color variation and stability, molecular design of those photochromic molecules has been done extensively [17–19]. Host-guests systems and supramolecular systems containing photochromic moieties have been designed for possible smart materials (photoresponsive materials) [20–22].

## 3. Photoinduced adsorption/desorption on/from solids

Photoswitching of gas adsorption has been achieved by combining photoresponsive molecular units and nanospace materials, such as mesoporous silicas (MPSs) [23–25], metal-organic frameworks (MOF) and clay minerals [26,27]. Optical illumination, change in local temperature, void volume, polarity in pore, and pore structure, as well as open/close the pore entrances, were used as physical mechanisms of the photoswitching. In this section, photocontrol of gas adsorption on MOFs and layered materials such as clay minerals will be discussed.

### 3.1. Photoinduced adsorption/desorption onto metal-organic framework

**Figure 1.**
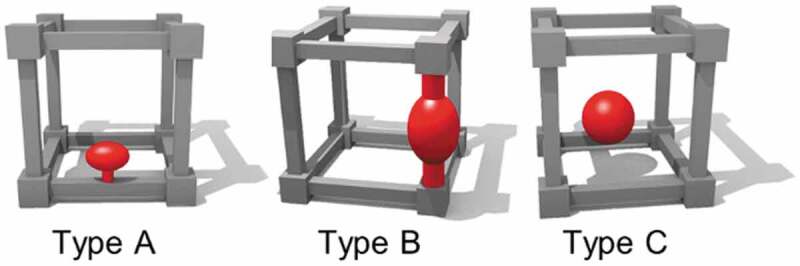
Schematic drawing for MOFs including photoresponsive moieties (Type A) in the side chain of linker, (Type B) in the framework backbone and (Type C) as the guest. (Reproduced from the reference [31] with permission).

Metal-organic framework (MOF) or porous coordination polymer (PCP) is a porous crystalline material consisting of metal ions or metal oxide clusters (node) and organic ligands (linkers) [28–31]. The porous structure of MOF has been used for gas storage [28] and molecular separation [32,33], in addition, photochromism of organic photochromic molecules in MOFs have been reported [34–37]. The photoresponsive MOFs are categorized into three types: MOFs with photoresponsive moieties (Type A) as the side chain of backbone, (Type B) in the framework backbone and (Type C) as the guest (Figure 1) [31]. Covalently attached photoresponsive units (Type A and B) have been more conveniently used to achieve photoinduced movements and there are few examples of photoresponses for Type C structure. The release of nitrogen (N_2_) gas from an azide incorporated in a MOF by the photodecomposition of the azide groups was reported [38]. Photochromism of azobenzene (unsubstituted AZ) adsorbed in MOF was used to switch the N_2_ adsorption capacity [39].

#### 3.1.1. Type A, MOFs including photochromic moiety in the side chain of linker

As the photochromic moieties were in the pore and on the external surface as shown in Figure 1, the photochromism of MOFs with photochromic side chains (Type A) does not accompany significant crystal lattice change. The polarity change in the pore by the photochromism caused the change in the host-guest interactions, and local heating by the light irradiation may also affect the response [40,41]. Photoswitching of the adsorption/desorption of N_2_ [39], methane [42], CO_2_ [40,41,43–47,47] and Ar [45,47] for Type A photofunctional MOFs has been reported.

**Figure 2.**
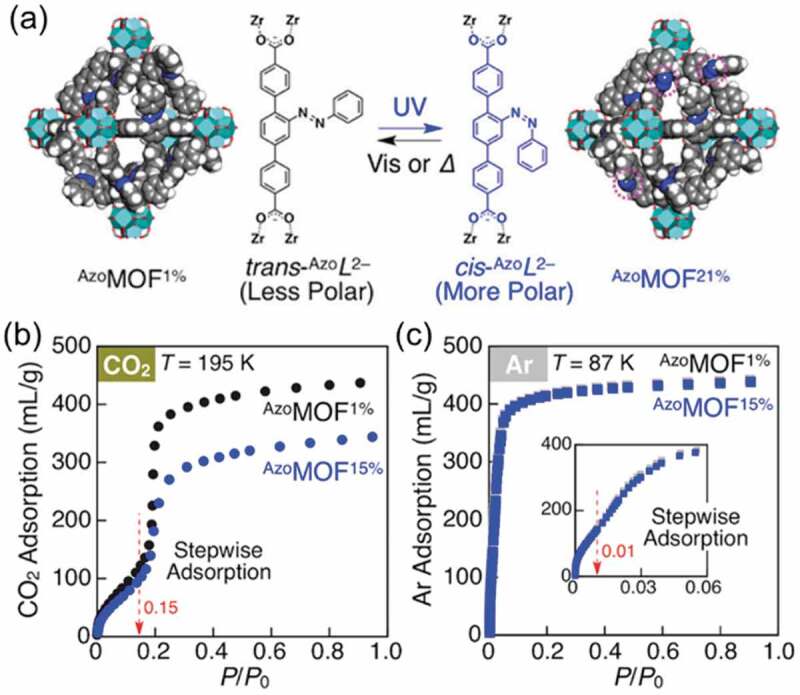
(a) Schematic representation of the MOF with azobenzene groups and adsorption isotherms of (b) CO_2_ and (c) Ar with the *cis*-isomer ratios of 1% (black dots) and 15% (blue dots). (Reproduced from the reference [45] with permission).

As shown in Figure 2, the adsorption of CO_2_ on a MOF containing zirconium oxo cluster as the node (^Azo^MOF, Zr_6_O_4_(OH)_4_L_6_; L^2−^ = 2′-phenyldiazenyl-1,1′:4′,1′′-terphenyl-4,4′′-dicarboxylate) largely depended on the *cis*-/*trans*-isomer ratio, while the adsorption of Ar was not affected by the *cis*-/*trans*-isomer ratio [45]. The effect of the polarity of gasses on the diffusion in ^Azo^MOF was examined from Equation (1) [48]. It was hypothesized that the diffusion of gasses in ^Azo^MOF occurs through two types; diffusion through triangular windows and in polyhedral pores with diffusion constants ^*W*^*k*_*d*_ and ^*P*^*k*_*d*_, respectively.

(1) $$M_{t}/M_{e}=A_{1}[1-exp\left({-}^{W}k_{d}t\right)+A_{2}1-exp\left({-}^{P}k_{d}t\right)]$$

*M*_*t*_ and *M*_*e*_ represent the amounts of gas molecules adsorbed at time *t* and at equilibrium, respectively, whereas *A*_*1*_ and *A*_*2*_ denote relative contributions of the triangular window and polyhedral pores. The ^*W*^*k*_*d*_ and ^*P*^*k*_*d*_ values for Ar were almost same at high (15%) and low (1%) *cis*-/*trans*-AZ ratio. On the other hand, the ^*W*^*k*_*d*_ (0.04 s^−1^) for CO_2_ at the lower *cis*-/*trans*-AZ ratio was larger than that at the higher *cis*-/*trans*-ratio (0 s^−1^), although ^*P*^*k*_*d*_ values were same irrespective of the ratio. Taking the sizes of CO_2_ and Ar into account, the polar *cis*-AZ disturbed the diffusion through the trihedral window of the MOF by the dipole-dipole interactions.

A SP functionalized MOF-808 ([Zr_6_O_4_(OH)_4_(btc)_2_(HCOO)_5.95_(P)_0.03_(SP-MeCOOH)_0.02_], where btc: 1,3,5-benzenetricarboxylate, P: 1-(carboxymethyl)-2,3,3-trimethyl-3*H*-indol-ium iodide (precursor of SP), SP-MeCOOH: 2-(3′,3′-dimethyl-6-nitrospiro-[chromene-2,2′-indolin]-1′-yl)acetic acid), was synthesized by post-synthetic grafting and showed the photoswitching of adsorption of Ar and CO_2_ gasses [47]. The adsorbed amount of Ar decreased by UV irradiation, while that of CO_2_ increased. The generation of polar MC was thought to contribute the increase in the adsorbed amount of CO_2_.

#### 3.1.2. Type B, MOFs with photochromic framework backbone

For Type B MOF, pore structure of MOFs transforms by the photochromism. Because of the rigidity of the framework, some MOFs did not accept the structural change to be photochemically silent [35,49–54]. The photoinduced change in the pore structure led to the controlled adsorption of gasses and the change in the crystal shape [14,55]. Photoinduced adsorption/desorption of gasses by the Type B photofunctional MOFs was reported using AZ [56–61], DAE [62] and SB [63] as photoresponsive units.

**Figure 3.**
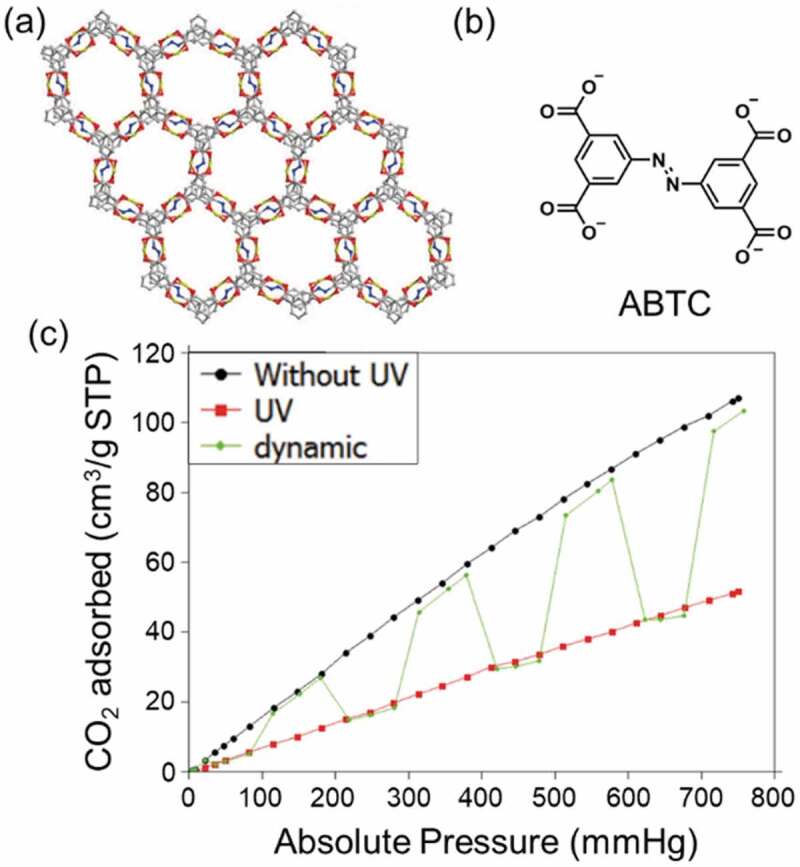
(a) Crystal structure of JUC-62, (b) molecular structure of AZ linker for JUC-62 (ABTC) and (c) CO_2_ adsorption of JUC-62 in static and dynamic condition at 273 K. (Reproduced from the reference [61] with permission).

Because the crystal structure did not accept the drastic change of the molecular structure of AZ [35], some reports concluded that local molecular conformation change [61] and heat generated by light absorption [60] affected the adsorbed amount more than the pore structure change. By UV irradiation, adsorbed amount of CO_2_ on a MOF, JUC-62 (Cu_2_(ABTC)(H_2_O)_2_·(DMF)_2_(H_2_O); ABTC; 3,3′,5,5′-azobenzenetetracarboxylate anion) changed from 102 cm^3^/g to 52 cm^3^/g at standard pressure and temperature (STP) (Figure 3) [61,64]. UV-vis absorption spectrum of JUC-62 changed slightly before and after the UV irradiation while IR spectrum changed by the UV irradiation, suggesting that small conformation change of ABTC in JUC-62 affected the adsorption of CO_2_. In order to use the change of the molecular structure of AZ for the change in the crystal structure of MOFs, DUT-49 [65] was functionalized with AZ linkers [66]. The MOF showed effective photochromism and contraction of the crystal structure with 0.1–0.15 mm sample thickness by the UV irradiation, although photoswitching of the adsorbed amount of H_2_ was not observed. DUT-49 was known to be softened to accept disordering of the crystal structure by functionalizing with 4,4′-diamino-SB [67,68].

**Figure 4.**
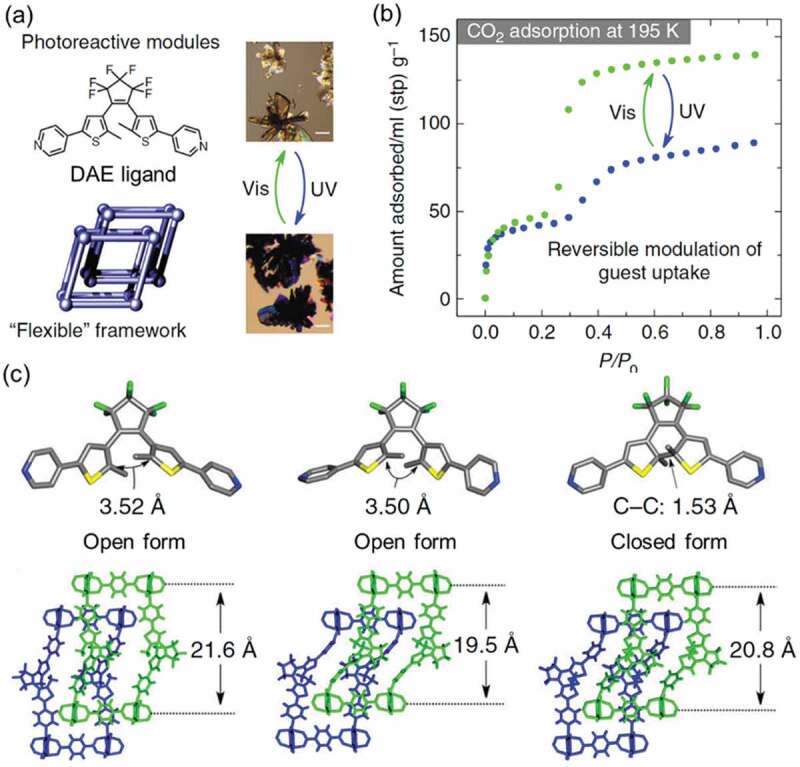
(a) Molecular structure of DAE ligand and photograph for color change of the MOF, (b) adsorption isotherms for CO_2_ of the DAE-functionalized MOF before and after UV irradiation at 195 K and (c) crystal structure the MOF (left) as prepared, (middle) guest free and (right) after the UV irradiation. (Reproduced from the reference [62] with permission).

As the photochromism of DAE accompanies relatively small change in molecular structure, the photochromism was acceptable in MOFs. As shown in Figure 4, the change of the crystal structure of a DAE-substituted MOF ([Zn_4_(bdc)_4_(DAE)_2_·4DMF·H_2_O]_n_, bdc; 1,4-benzenedicarboxylate anion) induced switching of adsorption/desorption of CO_2_ by UV irradiation [62]. Before the UV irradiation, the adsorbed amount of CO_2_ was 136 cm^3^/g (STP), which was 96 cm^3^/g (STP) after the UV irradiation.

#### 3.1.3. Type C, MOFs with photochromic guests

Photochromic compounds were incorporated in MOFs to give Type C structure [69–72]. Photoinduced adsorption/desorption of N_2_ by a MOF, [Zn_2_(terephthalate)_2_(triethylenediamine)]_n_, loaded with azobenzene (unsubstituted AZ) was reported [39]. Azobenzene was incorporated into the 2D pore (7.5 Å ×7.5 Å) of the MOF by soaking the MOF in molten azobenzene at 120°C. By UV irradiation, the adsorbed amount of N_2_ on the azobenzene loaded MOF increased from ca. 5 to 45 mL g^−1^ by the photoisomerization of the incorporated azobenzene. The difference in the adsorbed amount of N_2_ caused by the UV irradiation was explained by the structural change of the MOF, which was shown by powder X-ray diffraction patterns (XRD). After the subsequent heating at 120°C, the structure of the MOF returned to the original one as the incorporated azobenzene returned to *trans*-isomer, which was not achieved by light irradiation. After the heating, the MOF adsorbed the same amount of N_2_ as the MOF adsorbed before the UV irradiation.

Photoinduced adsorption/desorption of gasses by MOFs in Type A [46] and Type B [59] has been used for gas separation. Selectivity coefficients *s*_*1,2*_ were derived as follows [73],

(2) $$s_{1,2}=\frac{x_{1}/y_{1}}{x_{2}/y_{2}}=\frac{{P_{2}}^{0}}{{P_{1}}^{0}}$$

Where, *x*_*i*_ and *y*_*i*_ (*i* = 1 and 2) are mole fractions of component *i* in adsorbed and gas phases, respectively, and *P*_*i*_^*0*^ is equilibrium pressure of pure component *i*. The *s*_*1,2*_ values for CO_2_ against CH_4_ and N_2_ before and after UV irradiation were different in a Zn based MOF with an AZ side chain ligand (2-(phenyldiazenyl) terephthalate) [43] adsorbed tetraethylenepentamine [46]. The *s*_*1,2*_ values for CO_2_ against CH_4_ and N_2_ before the UV irradiation was 456 and 334 at 273 K and 0.1 bar, which became 92 and 137 after the UV irradiation. It was thought that the intercalated tetraethylenepentamine acted as an adsorption site for CO_2_ and the polar *cis*-AZ ligand interacted with the tetraethylenepentamine to disturb the adsorption of CO_2_. Adsorbed amount of CO_2_ was larger than those of CH_4_ and N_2_ and the differences of the adsorption of CH_4_ and N_2_ before and after the irradiation were smaller than that of CO_2_, suggesting possible photoinduced gas separation. Furthermore, the photocontrolled gas adsorption by the MOFs is expected to be applied as a light harvesting system by using the heat generation by the light irradiation [74] and for the decomposition of the adsorbed gasses by the hybridized photocatalyst [75].

### 3.2. Photoinduced structural change of layered materials incorporating photochromic compounds

Layered materials provides large surface area so that they have been used as adsorbent for noble species and pollutants from environments [76,77]. The controlled release of drugs and pesticide/herbicide has also been well documented [78]. Smectite group of clay minerals, which is composed of negatively charged silicate layer and interlayer exchangeable cations [79–81], is well-investigated layered materials for a wide variety of application.　In addition to smectites, there are several ion exchangeable layered solids used for the photoresponsive materials [82]. Cationic photochromic molecules have been intercalated [78,83,84] to show unique photochromic phenomena such as improvement of reversibility of photochromic reaction of DAE [85–87], controlled selectivity of dimerization/isomerization of cinnamic acids in layered double hydroxides (LDHs) [88–91] and SB in smectites [89,91–93], and photoresponsive magnetic layered materials including layered cobalt oxides [94–101].

**Figure 5.**
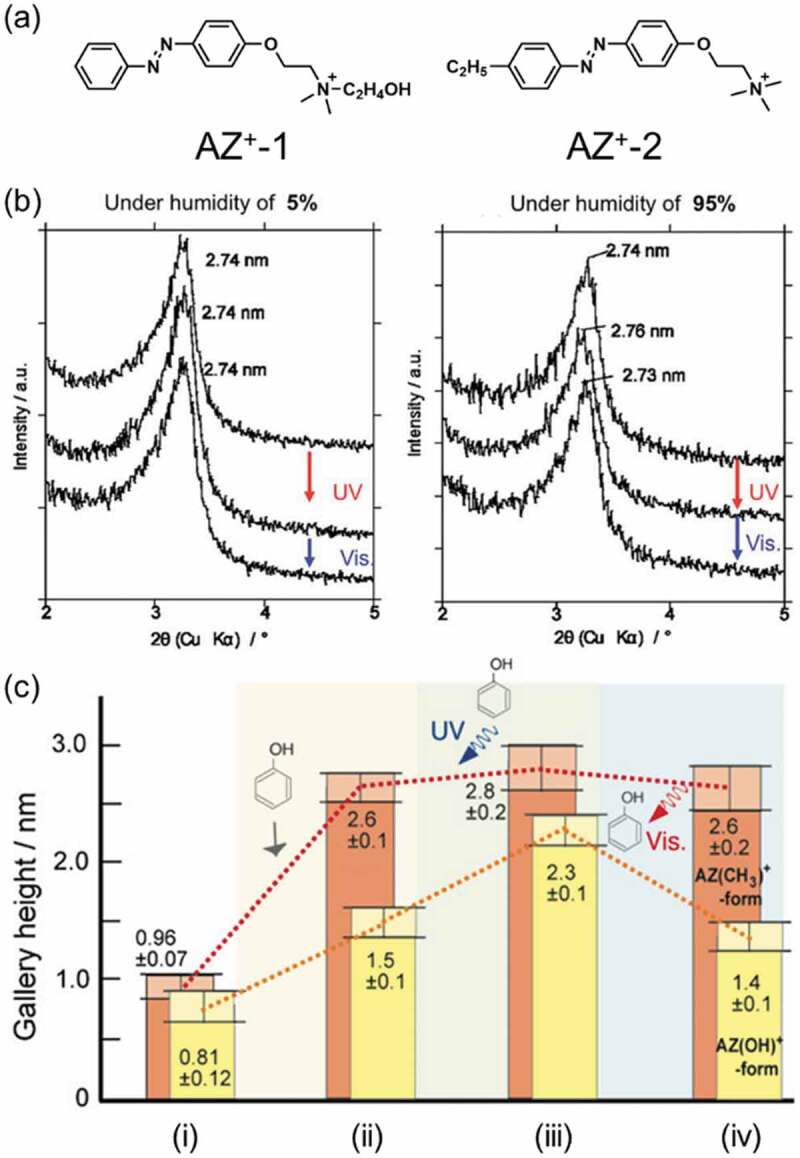
(a) Molecular structure of the cationic AZs used, (b) changes in the interlayer spacing of magadiite recorded under the humidity of 5% (left) and 95% (right). (c) the change in the gallery heights of AZ^+^-1 and AZ^+^-2 intercalated montmorillonites; (i) before the intercalation of phenol, (ii) after the phenol intercalation, (iii) after the UV irradiation and (iv) after the subsequent visible light irradiation. (Reproduced from the references [103] and [104] with permission).

As the first example of photoinduced structural change of the intercalation compounds, ionically neutral *p*-aminoazobenzene was incorporated into the interlayer space of a smectite after the organophilic modification [102]. Later on, cationic AZs were synthesized and were intercalated into the interlayer spaces of various layered silicates including smectites. The basal spacing increased by 0.06 nm in AZ^+^-1-magadiite (the change is shown in Figure 5(a)) [105,106]. The basal spacing of an AZ+-1 intercalated montmorillonite with the cation exchange capacity (CEC) of 119 meq/100 g did not change by the photoisomerization [107] while that of AZ^+^-1-magadiite (CEC: 159 meq/100 g) changed [108,109]. The basal spacings of the *p*-aminoazobenzene- and 4,4′-diaminoazobenzene-montmorillonite (CEC: 143 meq/100 g) were examined by molecular dynamics simulation in order to discuss the mechanism of the changes in the basal spacings observed by the experiments [110]. The basal spacings decreased from 2.0 nm (for the *trans-p*-aminoazobenzene-montmorillonite) and 2.1 nm (for the *trans*-4,4′-diaminoazobenzene-montmorillonite) to 1.8 nm for the both *cis*-products, which was not consistent with the experimental results showing the increase of the basal spacing from 3.0 nm to 3.1 nm [102]. This suggested that the change in basal spacing observed in the experiments was caused by adsorption/desorption of molecular species (such as water) to/from the products from/to the environments. As shown in Figure 5(b), the basal spacing of AZ^+^-1-magadiite increased by UV irradiation under the humidity of 95%, while it did not change under the humidity of 5%, indicating that photoinduced adsorption of water in air was important for the changes in the basal spacing [103].

Photoisomerization of azobenzene (unsubstituted AZ) in organophilic clays led to a decrease in the basal spacing [102]. The change was observed in fluor-tetrasilicic mica intercalated with azobenzene and trimethylalkylammonium with varied alkyl chain length (C_12_, C_14_, C_16_ and C_18_) and the exchange ratio (ammonium surfactant/CEC, varied from 0.25 to 2.0) [111,112]. Trimethyloctadecylammonium surfactant (C_18_) formed bimolecular paraffin-type aggregate in fluor-tetrasilicic mica. The basal spacing of C_18_-fluor-tetrasilicic mica did not change by photoisomerization of incorporated azobenzene [112,113], while the basal spacing became smaller when trimethyldodecylammonium surfactant (C_12_), which formed *pseudo*-trimolecular layer in the interlayer space, was used [113,114]. Azobenzene was intercalated from vapor into trimethylalkylammonium-beidellite with varied alkyl chains (12, 14 and 16) and the changes in the basal spacing upon irradiation were larger when shorter alkyl chain was used [115].

**Figure 6.**
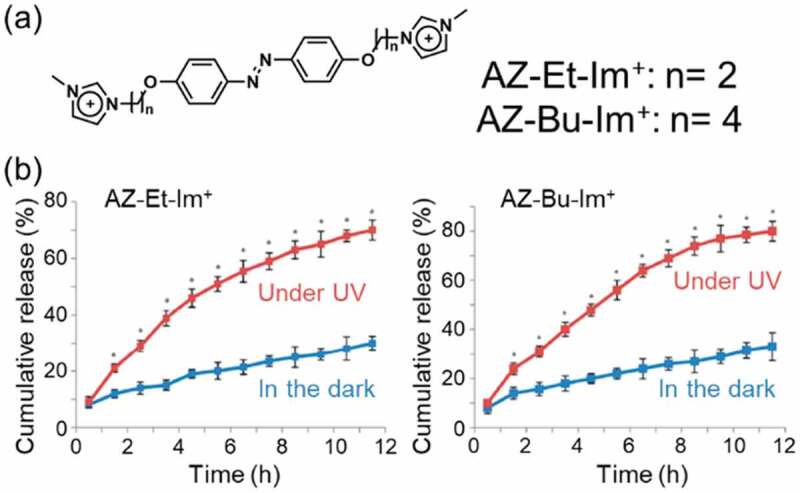
(a) Molecular structure of imidazolium cation substituted AZs and (b) in vitro release profiles of *p*-aminobenzoic acid from the AZ-Et-Im^+^ and AZ-Bu-Im^+^ intercalated montmorillonites in the dark and under UV radiation at 34 ± 1°C in phosphate buffer solution. (Reproduced from the reference [116] with permission).

Photoinduced adsorption and release [116] of nonionic organic molecules [104,117,118] were reported for cationic AZ intercalated smectites. As shown in Figure 5(c), the basal spacings of AZ^+^-1- and AZ^+^-2-montmorillonites increased by the adsorption of phenol form water [104]. The adsorbed amount of phenol increased by UV irradiation, which was suggested by the increase of the basal spacing, and returned to the initial values by subsequent visible light irradiation. Imidazolium cation substituted AZs (AZ-Et-Im^+^ and AZ-Bu-Im^+^, the molecular structures are shown in Figure 6(a)) were intercalated in a montmorillonite from water/ethanol ( = 5/3) mixture and subsequently a model drug, *p*-aminobenzoic acid, was intercalated to the hybrids [116]. The *p*-aminobenzoic acid loaded AZ/montmorillonite hybrids were placed in a phosphate buffer (pH 5.8) at 34 ± 1°C in the dark and release ratios of the model drug by UV irradiation was examined. As shown in Figure 6(b), the released ratios in the dark in 12 h were 30% for AZ-Et-Im^+^ and 33% for AZ-Bu-Im^+^, while those under the UV irradiation were 71% for AZ-Et-Im^+^ and 80% for AZ-Bu-Im^+^, respectively. It was thought that increase of the basal spacing was originated by the photoisomerization of AZ-Et-Im^+^ (0.06 nm) and AZ-Bu-Im^+^ (0.10 nm) in the interlayer space, which resulted in the efficient release of the model drug by the irradiation.

The particle shape is expected to change by the photoisomerization of the intercalated AZ in the layered materials, and as examples, sliding of niobate nanosheet in μm scale [119–122] and bending motion of a layered siloxane film in mm scale [123] have been reported. These morphological changes may involve associating changes such as volume, lubricant, rheology, etc. so it is worth investigating further from different aspects also.

## 4. Ion and molecular transport through liquid-liquid interface

Extraction of solute from solution by using immiscible solvents combination is a common way of separation and purification. Photoinduced migration of a molecule between two immiscible solvents through liquid-liquid interface has been reported as a smart separation triggered by light. The systems can be categorized into two; photoresponsive materials are 1) dissolved and move with the target species through the interface and 2) fixed on the membrane to act as valves.

### 4.1. Molecular migration through liquid-liquid interface

**Figure 7.**
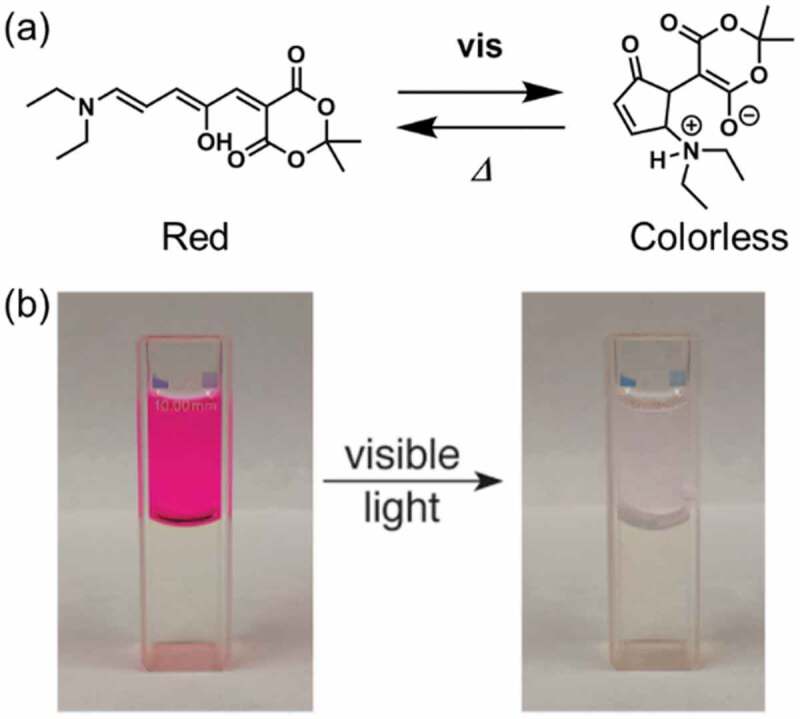
(a) Photochromism of DASA and (b) photoinduced migration from toluene (upper) to water (bottom) by visible light irradiation. (Reproduced from the reference [124] with permission).

Donor-acceptor Stenhouse adduct (DASA) [124] changes its electronic state by light irradiation to give zwitter ionic colorless state and it shows negative photochromism in non-polar environment thanks to the long π-conjugated system as the thermodynamically stable colored state (Figure 7(a)) [125,126]. The change in the electronic state led photoinduced migration of DASA between water (for colorless) and toluene (for colored), which is shown in Figure 7(b).

### 4.2. Ion transport through liquid membrane by photoresponsive molecules

**Figure 8.**
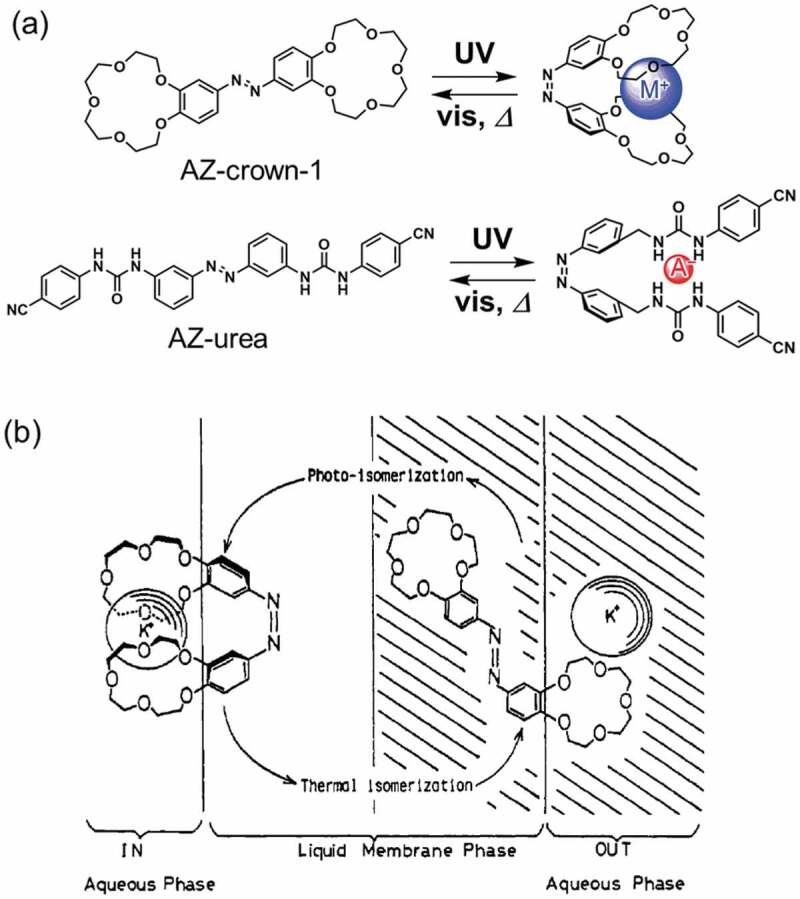
Photochromism of AZ-crown-1 with the complexation with metal cations (blue spheres) and AZ-urea with anion (red sphere) and (b) schematic representation of cation transfer through liquid membrane. (Reproduced from the reference [7] with permission).

The photoresponsive molecules were not fixed at liquid-liquid interface and acted as vehicles for ion transport through the membrane. The ion transport was triggered by photochromism [127] of such as AZ [7,128–130] and SP [131–133], photoinduced charge separation of porphyrin [134–138] and photodecomposition of ligand in a metal complex [139]. The first example of the photoinduced cation transport through liquid-liquid interface was reported by Shinkai et al [7]. Photoisomerization of AZ-crown-1 (shown in Figure 8(a)) enabled photoswitching of its metal cation capturing [128–149]. By the change of the affinity between AZ-crown-1 and the metal cations, alkali metal cations were transported through a liquid membrane by irradiation [7,128–130]. Cation binding ability of both the *trans*- and *cis*-isomers of AZ-crown-1 was estimated by the solvent extraction of metal salts of methyl orange from water to *o*-dichlorobenzene induced by irradiation. The binding ability for *cis*-AZ-crown-1 for K^+^ (55.2%) was 42.5 times higher than that (1.3%) of *trans*-AZ-crown-1 [7]. The photoinduced transport of the metal cations was also examined in a U-shaped tube as shown in Figure 8(b). Two aqueous solutions were separated by the liquid membrane of *o*-dichlorobenzene containing AZ-crown-1. An aqueous solution (left side in Figure 8(b) noted ‘IN’) contained metal cations and an indicator (2-nitrodiphenylamine-4-sulfonic acid) and the other solution (right side in Figure 8(b) noted ‘OUT’) did not contain metal ions. Absorbance due to the indicator in the right side solution was measured to quantify the cation transport to show that the transport of K^+^ by AZ-crown-1 under high pressure Hg lamp (0.34 μmol/h) was 17 times higher than that (0.02 μmol/h) in the dark.

By using this concept, anion transport was also achieved using AZ-urea (the molecular structure is shown in Figure 8(a)) [150–152]. AZ-urea had two urea substituents and the *cis*-isomer interacted with Cl^−^ effectively. An association constant of *cis*-AZ-urea for Cl^−^ (8400 M^−1^) was 15 times larger than that of *trans*-AZ-urea (570 M^−1^) [150]. Fluorescence intensity in Fischer rat thyroid epithelial cells including halide sensitive fluorescent proteins (YFP) was used to quantify the halide in a cell separated by a plasma membrane. The concentration of Cl^−^ decreased to 90% of the initial concentration by UV irradiation for 2 h while the concentration did not change in the dark. These observations indicated that AZ-urea accommodated Cl^−^ to transport across the plasma membrane. A cyanide ion sensor was proposed by using the different interactions between the substituents of the two phenyl rings of AZ and cyanide ion [153]. In addition, photoinduced adsorption/desorption of metal cations on polymer beads crosslinked with AZ-crown-1 [154], and photoinduced extraction of metal cations from water to organic solvent [155–159] and transport between two liquid phases through a liquid membrane [131–133] with crown ether functionalized SP were reported. Proton transport by utilizing protonated MC which released proton by light irradiation (Scheme 1(d)) was reported to make electric voltage (up to 210 mV) [133].

**Figure 9.**
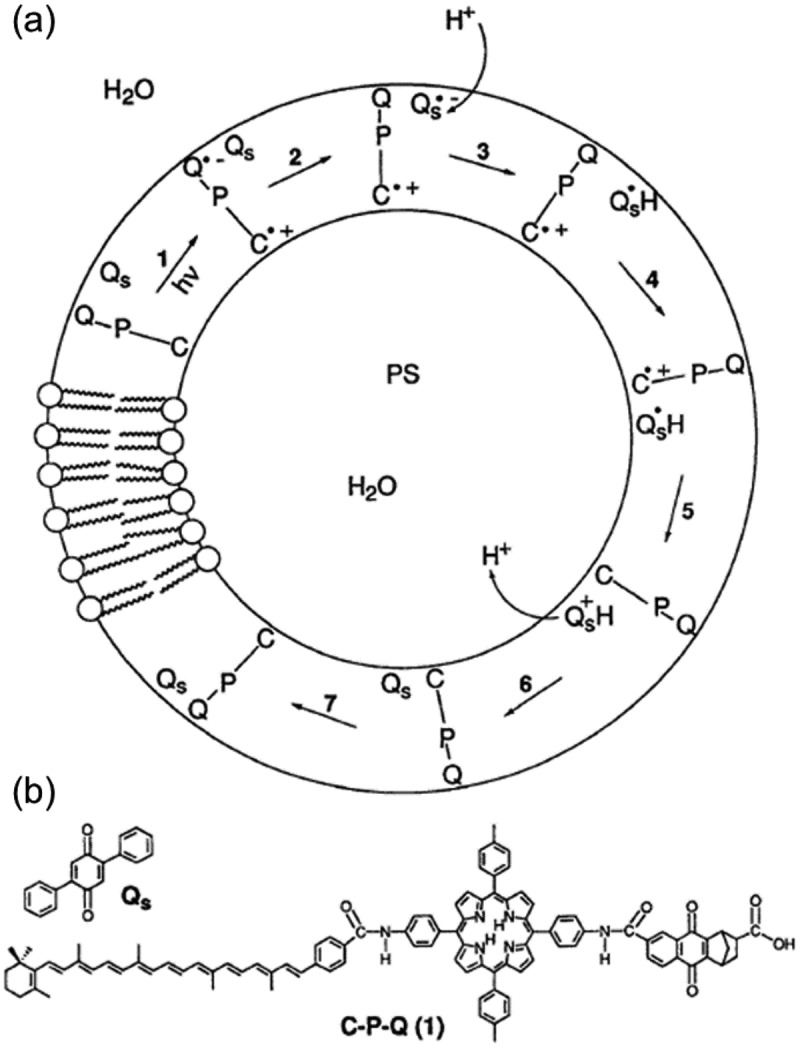
(a) Photocatalytic cycle of proton transfer through liposome membrane and (b) molecular structure of Q_s_ and C-P-Q. (Reproduced from the reference [134] with permission).

Inspired by the light-driven cation pumps in biological systems seen in bacteriorhodopsin [160,161] and photosynthesis, cation transport through lipid bilayer membranes of liposome was designed [134–137]. As shown in Figure 9, a photocatalytic cycle of cation transport was achieved in the liposome incorporating 2,5-diphenylbenzoquinone (Q_s_) and molecular triad (C-P-Q) composed of tetraarylporphyrin (P), naphthoquinone with norbornene and carboxylic acid (Q) and carotenoid (C) [134]. The photocatalytic cycle progressed as 1) photoexcitation of P induced electron transfer from C to Q in order to form a charge separated state (C^∙+^-P-Q^∙−^), 2) Q_s_ accepted the electron from the radical anion (Q^∙−^) and converted to a radical anion Q_s_^∙−^, 3) Q_s_^∙−^ reacted with H^+^ in water outside of the liposome, 4) the protonated radical Q_s_H^∙^ moved into the lipid layer and 5) oxidation of Q_s_H^∙^ by the radical cation C^∙+^ in the triad to regenerate the initial state of the triad (C-P-Q) with emitting H^+^ to inside of the liposome. Each intermediate was characterized by transient absorption spectroscopy. The local pH value in the liposome was estimated by the pH dependent emission of 8-hydroxypyrene-1,3,6-trisulfonic acid (HPTS) at 510 nm [134]. ATP was synthesized by the photoinduced H^+^ transfer with the molecular triad in the liposome with F_0_F_1_-ATP synthase [135]. When hydroquinone derivative (1-(2,5-dihydroxyphenyl)hexadecan-1-one) was used instead of Q_s_, transport of Ca^2+^ was reported [137].

**Figure 10.**
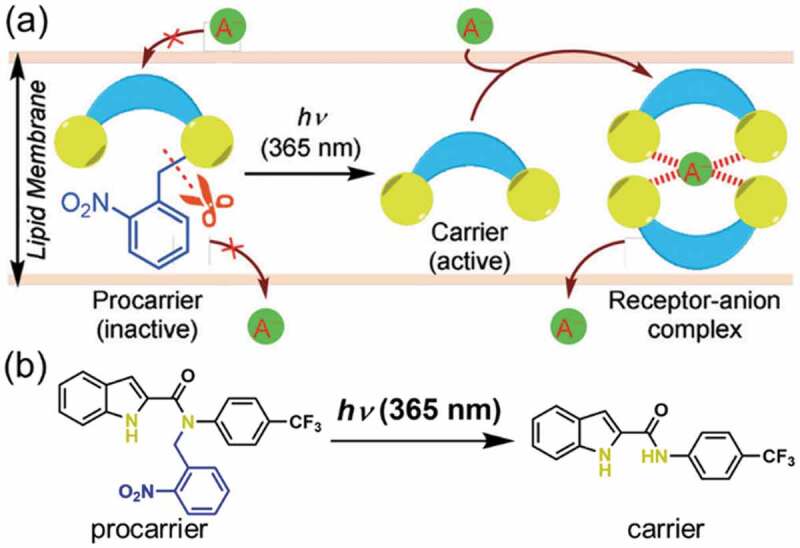
(a) Schematic representation of anion transfer and (b) molecular structure of procarrier and carrier. (Reproduced from the reference [139] with permission).

Anion (Cl^−^) transport was also achieved by photodecomposition of *N*-phenyl-1*H*-indole-2-carboxamides named as ‘procarrier’. The schematic representation of the transport is shown in Figure 10 [139]. The procarrier (shown in Figure 10(b)) decomposed by UV irradiation to give ‘carrier’ which formed a complex with Cl^−^. The complex transported Cl^−^ into vesicle of egg-yolk phosphatidylcholine (EYPC) following the concentration gradient. After the UV irradiation, fluorescence from HPTS in the vesicle at 510 nm increased. The fluorescence of HPTS at 510 nm was attributed to the protonated HPTS, and the protonation occurred at lower pH probably caused by Cl^−^ in the present system. Thus, the change in the fluorescence of HPTS at 510 nm was used to shown the transportation of Cl^−^. The Cl^−^ amount increased by the light irradiation while no change was seen without the irradiation.

### 4.3. Ion transfer using photoresponsive membrane

The development of an artificial ion pump driven with sun light is expected to provide a novel light harvesting system [133,162,163]. Materials mimicking the ion channels on 1) the modification of channel proteins and 2) the preparation of artificial ion channels in lipid bilayer have been examined in addition to 3) design fully artificial ion channel.

#### 4.3.1. Modification of ion channel

**Figure 11.**
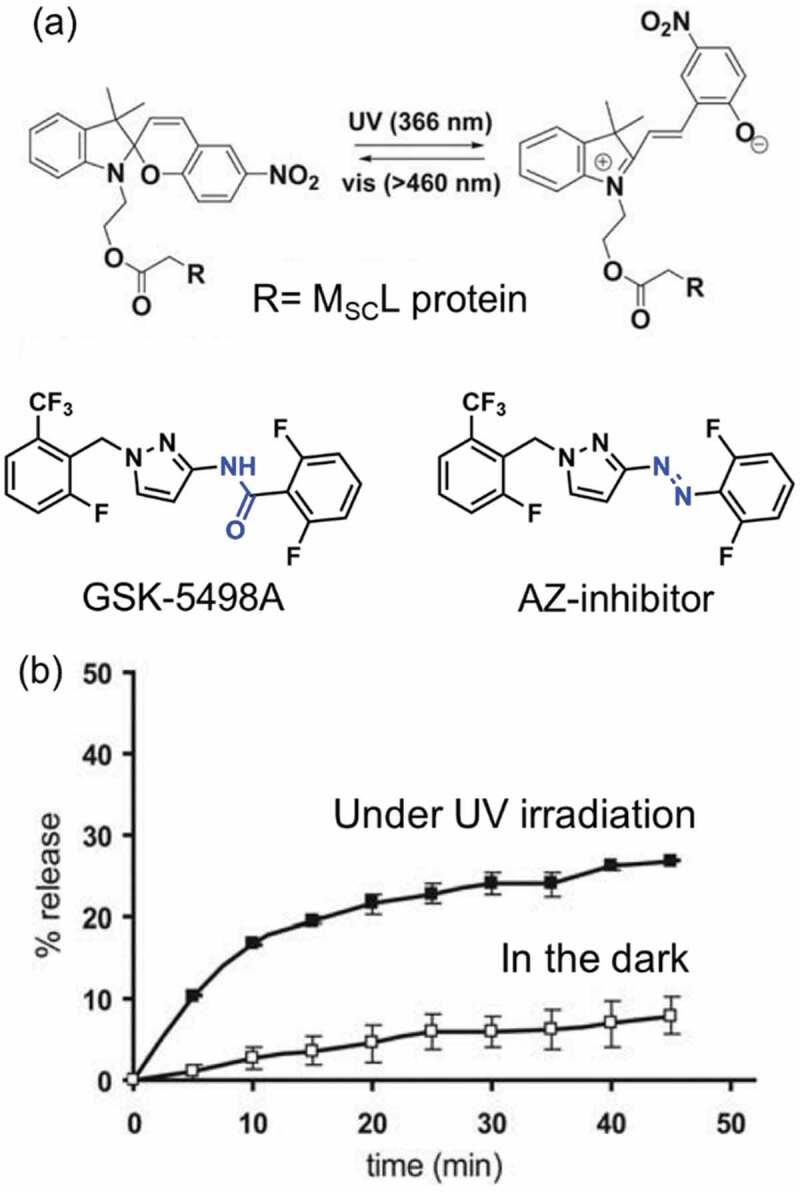
(a) Molecular structure of SP derivative to control M_SC_l, GSC-5498A and AZ-inhibitor and (b) photoinduced release of calcein from liposome with SP functionalized M_SC_l. (Reproduced from the reference [164] with permission).

Activation of channel proteins was controlled by the modification with photochromic moiety [164–167] and with an inhibitor containing a photochromic unit [168]. A channel protein, large conductance mechanosensitive channel (M_SC_L), acts as a stretch-activated osmotic release valve in the inner membrane of *E*. *coli* [169]. A glycine residue at 22^nd^ amino acid in M_SC_L was replaced with cysteine in order to incorporate a SP derivative as shown in Figure 11(a). The SP functionalized M_SC_L opened by UV irradiation thanks to the hydrophilic MC and the release of calcein from a liposome containing the M_SC_L functionalized with SP was observed as shown in Figure 11(b) [164, 165]. Two cysteine residues of SecY, which are parts of a gate in a protein-translocating channel (SecYEG) [170], were crosslinked with bis(bromomethyl)azobenzene. Translocation of a protein, proOmpA, was stopped by the UV irradiation, which indicated that the gate was closed by the shorter distance between two cysteine residues with the *cis*-isomer than that with the *trans*-isomer.

GroEl, which is a tubular assembly of 14 identical protein subunits [171], incorporates denatured proteins and assists the refolding of the proteins in presence of adenosine triphosphate (ATP). ATP has a role in opening the cavity of GroEL to release the refolded protein. A cysteine at 231^st^ in GroEL was functionalized with 4-carboxyl-AZ to control the release of incorporated proteins from GroEL [166,167]. Green fluorescence protein (GFP) was incorporated in the AZ functionalized GroEL in a tris(hydroxymethyl)aminomethane hydrochloride (Tris-HCl) buffer. The release of GFP was monitored by fluorescence. The release of GFP from the AZ functionalized GroEL was initiated by the addition of ATP. The *cis*-AZ functionalized GroEL showed three times more efficient release of GFP than that from the *trans*-AZ functionalized GroEL, although unfunctionalized GroEL did not refold GFP. The difference in the accessibility of ATP to the *trans*- and *cis*-AZ functionalized GroELs was shown to affect the release of GFP.

An ion channel called calcium-release activated calcium (CRAC) channel is activated at low Ca^2+^ concentration to increase Ca^2+^ concentration in plasma membrane [172]. The activation of CRAC was inhibited by a channel blocker named GSK-5498A (Figure 11(a)) with a half maximal inhibitory concentration (IC_50_) of 3.10 μM [173]. Inspired by GSK-5498A, an AZ derivative (AZ-inhibitor, the molecular structure shown in Figure 11(a)) was designed to have the similar molecular structures with GSK-5498A [168]. The *trans*-AZ-inhibitor acted as a channel blocker for CRAC with IC_50_ of 73.2 μM, which was larger than that of GSK-5498A. On the other hand, *cis*-AZ-inhibitor, whose molecular structure was different from GSK-5498A, showed a lower IC_50_ value (0.5 μM) than *trans*-AZ-inhibitor and GSK-5498A. It was proposed that the structural difference between *trans*- and *cis*-AZ-inhibitor caused the change in CRAC.

#### 4.3.2. Artificial ion channel

**Figure 12.**
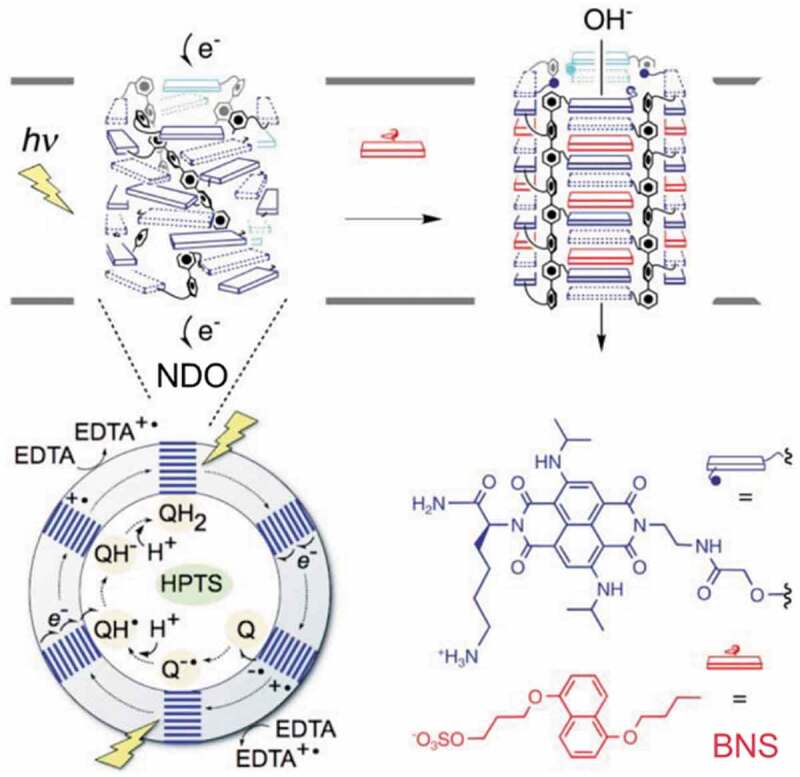
Photocatalytic electron transfer by naphthalene diimide octamer (NDO) and transformation by BNS to neutralize pH gradient. (Reproduced from the reference [178] with permission).

Artificial ion channels have been prepared in lipid bilayers [174–177] and photoinduced transmembrane molecular transport was achieved [178–182]. As shown in Figure 12, naphthalene diimide octamer (NDO, the structure is shown in Figure 12) formed a rod-like structure in a lipid bilayer of EYPC. NDO (Figure 12) generated a charge separated state by 635 nm irradiation to oxidize ethylenediaminetetraacetic acid (EDTA) and to reduce 1,4-naphthoquinone (Q), which caused pH difference [178]. Intercalation of 3-((5-butoxynaphthalen-1-yl)oxy)propyl hydrogen sulfate (BNS, the molecular structure is shown in Figure 12) induced transformation of the rod-like structure to ion channel with 0.48 nm pore. The ion channel transported OH^−^ to neutralize the pH difference between both sides of the lipid bilayer.

**Scheme 2.**
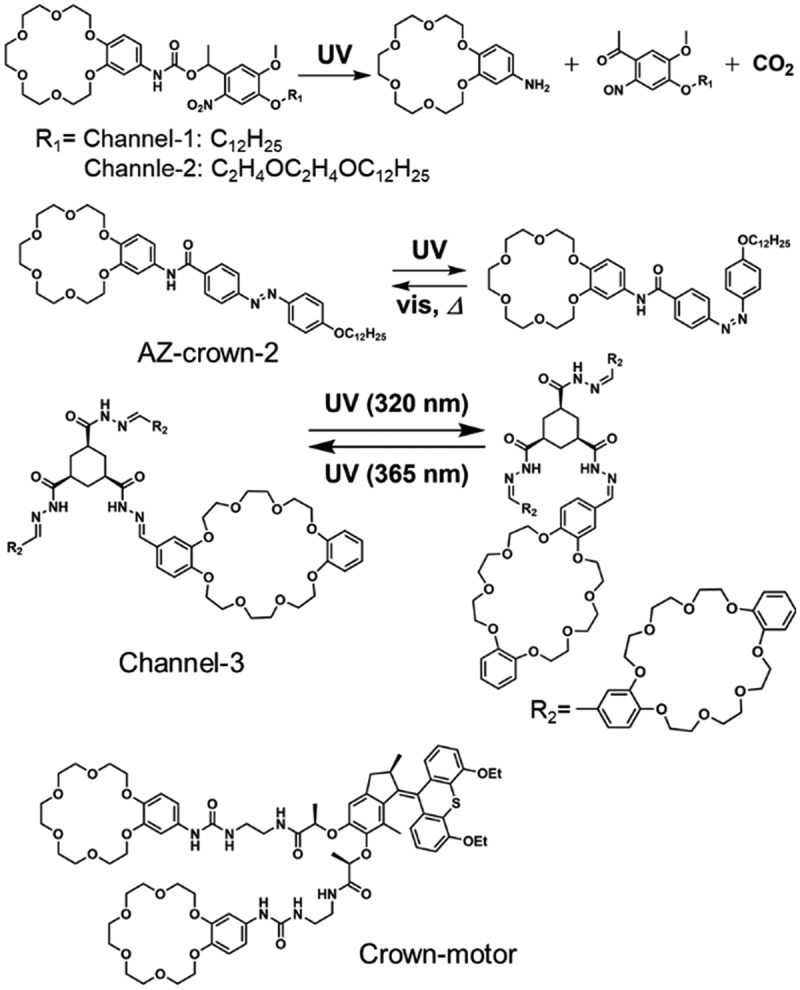
Molecular structure of artificial ion channels and their photoresponses.

Scheme 2 shows examples of the crown ethers connected with some photoresponsive units. Channel-1 and Channel-2, AZ-crown-2 and Crown-motor formed ion channels in the lipid bilayers of EYPC and Channel-3 did in diphytanoylphosphatidylcholine. The ion channels composed of Channel-1 and Channel-2 were decomposed by light irradiation to deactivate the ion transport. Reversible on/off switching of the ion transport was reported by using isomerization of AZ-crown-2, Chanel-3 and Crown-motor (Scheme 2) [180–182]. Vesicles of EYPC incorporating with AZ-crown-2 were prepared with encapsulating 8-hydroxy-1,3,6-pyrenetrisulfonate (HPTS) in an inner water phase [181]. The vesicle was irradiated UV light in an aqueous KCl solution, then, the increase of fluorescence at 425 nm ascribable to the emission from deprotonated HPTS was observed. The fluorescence at 425 nm was used as an indicator (probe) of the local environment of HPTS molecule. Deprotonation of HPTS was thought to occur by the transportation of K^+^ from the outer aqueous solution of the vesicle to the inside of the vesicle through the ion channel of AZ-crown-2 with simultaneous transportation of OH^−^ [183]. After the UV irradiation for 150 s, the fluorescence intensity became higher than that in the dark, suggesting the efficient transportation of K^+^ with *cis*-AZ-crown-2 compared to *trans*-AZ-crown-2, even though the channel size (0.58 nm) in *trans*-AZ-crown-2 was larger than that (0.28 nm) in *cis*-AZ-crown-2. The reason for the efficient transport through *cis*-AZ-crown-2 was proposed to be the hydrophilicity for *cis*-AZ-crown-2 in the channel. The photoswitching of fluorescence intensity caused by cation transport was also observed with Crown-motor (the molecular structure is shown in Scheme 2). The preferential cation transport at the order of Rb^+^> K^+^> Na^+^ was reported for Channel-3 and Crown-motor systems [180,182]. The sequence was attributed to the selectivity of alkali metal ions binding by the photoresponsive molecules shown in Scheme 2.

**Figure 13.**
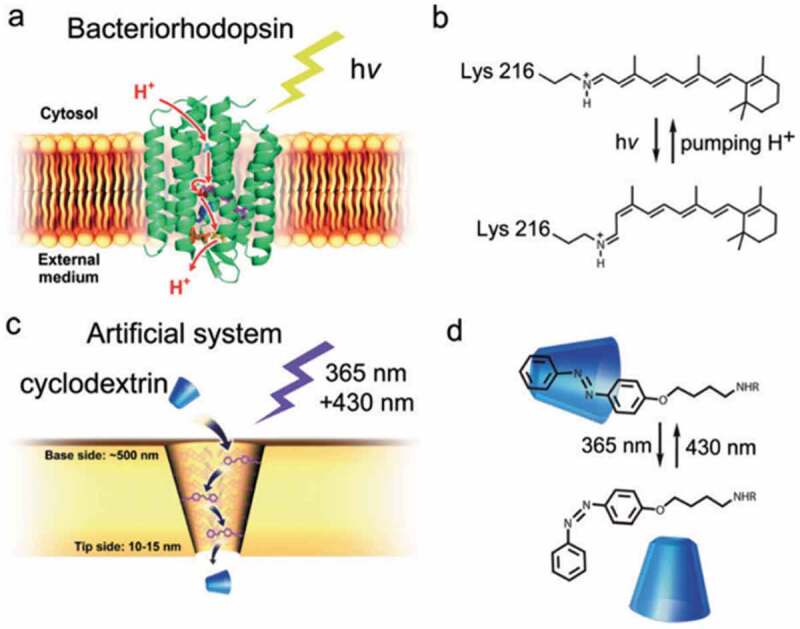
(a) The light-driven proton-pumping system of bacteriorhodopsin, (b) photochromism of a retinal Schiff base in bacteriorhodopsin, (c) bio-inspired light-driven CD-transportation system by (d) photoisomerization of AZ. (Reproduced from the reference [162] with permission).

Fully artificial ion channel has been prepared with conical shaped pores and photocontrol of impedance of the pores [184–187]. Fully photodriven ion pumps are scarcely reported [162]. As shown in Figure 13, a conical shaped pore was prepared in a polyimide membrane (thickness: 12 μm, pore size: 500/10–15 nm) and subsequently AZ moieties were incorporated by a reaction with surface COOH which was generated during the pore formation [162]. The charged molecules such as sulforhodamine B and rhodamine 6 G did not transport through the pore due to the hydrophobic pore surface given by the AZ moieties while cyclodextrins (CDs) transported by the UV irradiation, suggesting the higher affinity of CDs with *trans*-AZ than *cis*-AZ [188,189].

**Figure 14.**
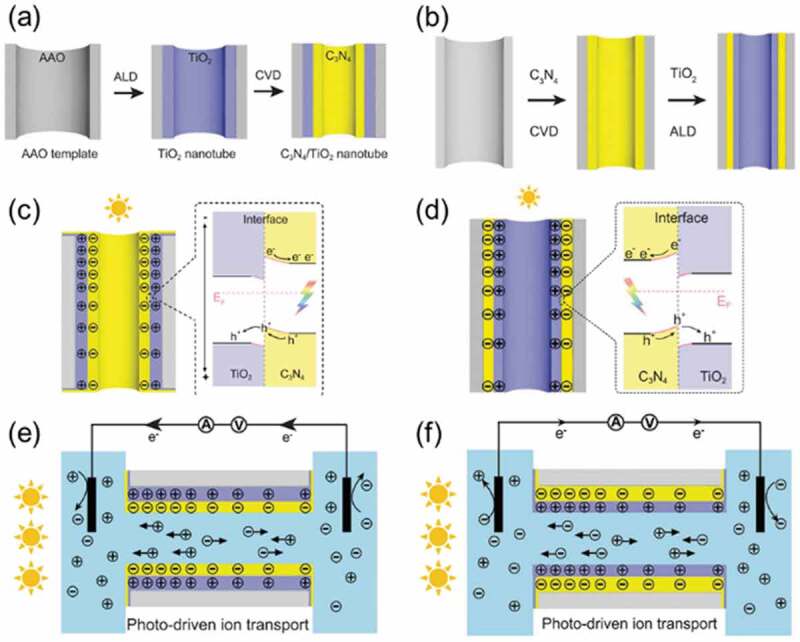
Schematic drawing of the fabrication of p/n heterojunction of TiO_2_/C_3_N_4_ (a) and (b), photoinduced charge separation in the heterojunctions (c) and (d) and ion transport under light irradiation (e) and (f). (Reproduced from the reference [163] with permission).

Ion transport through membrane was also achieved by photothermal effect [190]. Photoinduced charge separation between cationic and anionic polyacetylenes [191] and p/n heterojunction of TiO_2_/graphitic carbon nitride (g-C_3_N_4_) [163] was also used to trigger the ion transport. As shown in Figure 14(a), a porous anodic alumina (AAO) membrane with the cylindrical pore with the diameter of 100 nm was coated with TiO_2_ and g-C_3_N_4_ sequentially with the 10 nm thickness for each to prepare p/n heterojunction [163]. Light irradiation to the semiconductor induced charge separation and, as the result, localized negative charges were generated on the surface in the pore with the gradient to the light. The gradient of the negative charge induced cation transport to the light. On the other hand, the coating with reverse sequence (g-C_3_N_4_ => TiO_2_ as shown in Figure 14(b)) generated surface positive charges by the light irradiation and anion transport to the light was seen.

## 5. Photoinduced molecular migration in solid and on solid surface

Microscopic patterning such as gratings and dots is useful for lithography, holographic imaging, metamaterials and superhydrophobic surfaces. Mass transfer of polymer in film induced by light irradiation has been used to make images such as lithography and holography [192]. The light irradiation through a photomask or interfering laser to the polymer film induced the periodic surface texture (pattern), which is called as surface relief gratings (SRGs) [193]. The patterns were generated by molecular migration from irradiated to non-irradiated regions in the polymer films [194–197]. In this chapter, the strategy for the generation of SRG caused by photochemical reaction such as photodecomposition, polymerization and photoisomerization will be discussed. The surface tension change by the photochemical reaction leads to the motion of macroscopic objects.

### 5.1. Surface relief grating modified by photodecomposition and photopolymerization

In order to accelerate the SRG formation, photodecomposition [198,199] and photopolymerization [200–211] have been utilized. The doping of photosensitizer has also been examined to obtain SRG by longer and weaker light irradiation [212]. SRG formed in a cast film of mixture of polystyrene (PS) and poly(*tert*-butoxystyrene-*ran*-styrene) containing a photoacid, triphenylsulfonium nonaflate, by light (200–800 nm) irradiation for 1 min with a photomask and subsequent annealing at 120°C for 30 min [199]. *tert*-Butoxy groups in the copolymer were decomposed to hydroxyl groups by the photoacid to generate surface tension difference in the irradiated and the non-irradiated regions, leading to Marangoni flow, which is material flow caused by the difference in surface tension, of PS from the non-irradiated region to the irradiated region during the annealing.

**Figure 15.**
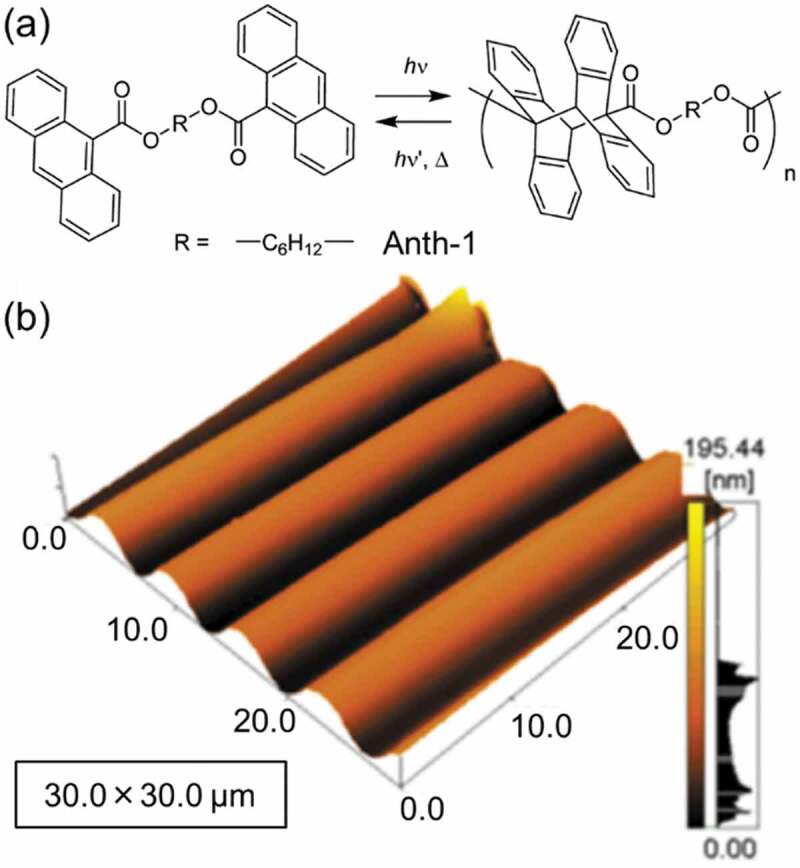
(a) Molecular structure of Anth-1 and (b) the AFM image of the resulting SRG formed in Anth-1 film by the UV irradiation (365 nm, 0.12 mW cm^−2^) for 10 min at 333 K under N_2_ atmosphere using photomask. (Reproduced from the reference [202] with permission).

Photopolymerization of diene [201], acrylates [200,204,205,209–211], epoxy [208], benzoic acid [203] and anthracene [202] has been used for the SRG formation. Because monomer was mobile during the light irradiation and photopolymer stabilized the SRG structure, effective formation and high thermal and photostability of SRG were achieved. However, the SRG was not rewritable as the polymerization is not reversible. In order to form rewritable SRG, a film of an anthracene derivative (Anth-1, the molecular structure is shown in Figure 15) with the thickness of 0.1 μm was prepared by spin coating [202]. SRG formation was seen by UV irradiation with a photomask (365 nm, 0.12 mW cm^−2^) at 333 K for 10 min. The AFM image of the SRG is shown in Figure 15(b). The SRG was stable at around 360 K and the structure was maintained at 390 K by the subsequent UV irradiation thanks to the dimerization of Anth-1. The effect of the back reaction to monomer was not reported, which is worth investigating to introduce rewritable property.

### 5.2. Surface relief grating by photochromism

Micrometer sized SRG formed in the systems containing photochromic units such as AZ [196,197,213–285], SP [286–289], SO [290], DAE [291], imidazole dimer [292], SB [293], phenylacetylene carboxylic acid [294], cinnamic acid [295–297], benzylideneaniline [298] and fluorene [299]. The importance of Marangoni flow was pointed out in the SRG formation in the systems containing AZ [300–323].

**Figure 16.**
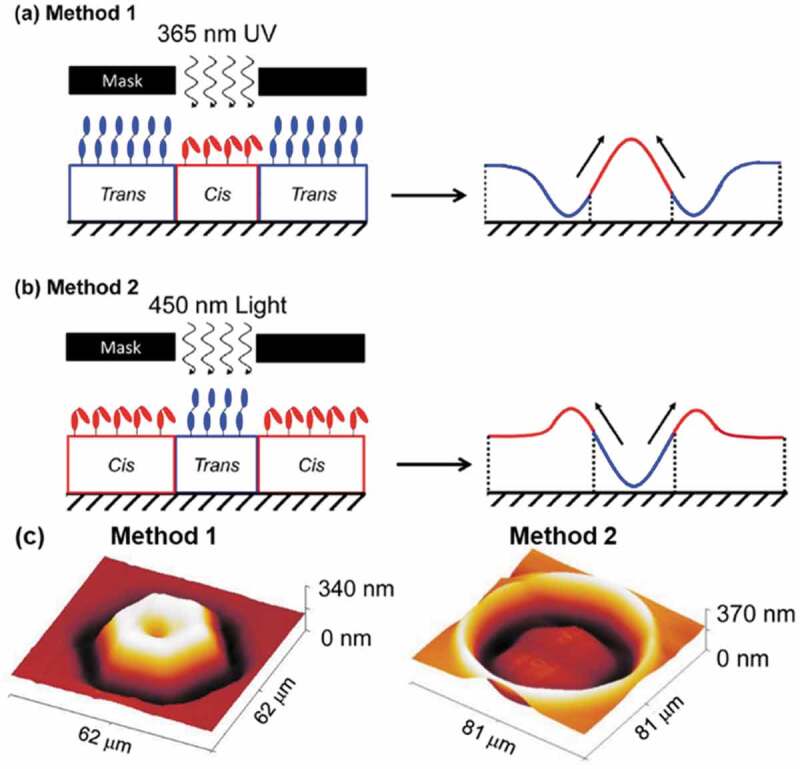
Schematic drawing of the SRG formation by irradiating to induce (a) *trans*-rich and (b) *cis*-rich polymer and (c) resulting surface structures. (Reproduced from the reference [269] with permission).

Extensive studies on the SRG formation suggested the importance of Marangoni flow induced by interactions among polymers and light [224,248,255,267,269,285,313]. As an example, the contact angle of 95° for a water droplet on the surface of *trans*-form of poly(4-(acryloyloxyhexyloxy)-4′-pentylazobenzene) was higher than that (73°) seen on the *cis*-form, suggesting that the surface tension in the *cis*-form was larger than that in the *trans*-form [269]. As shown in Figure 16, UV irradiation to a film with the *trans*-form induced mass flow from the non-irradiated (low surface tension) to the irradiated (high surface tension) areas. In contrast, visible light irradiation to a *cis*-rich film induced mass flow from the irradiated (low surface tension) to the non-irradiated (high surface tension) area. These facts suggested that the Marangoni flow played an important role on the SRG formation in the AZ polymers.

**Figure 17.**
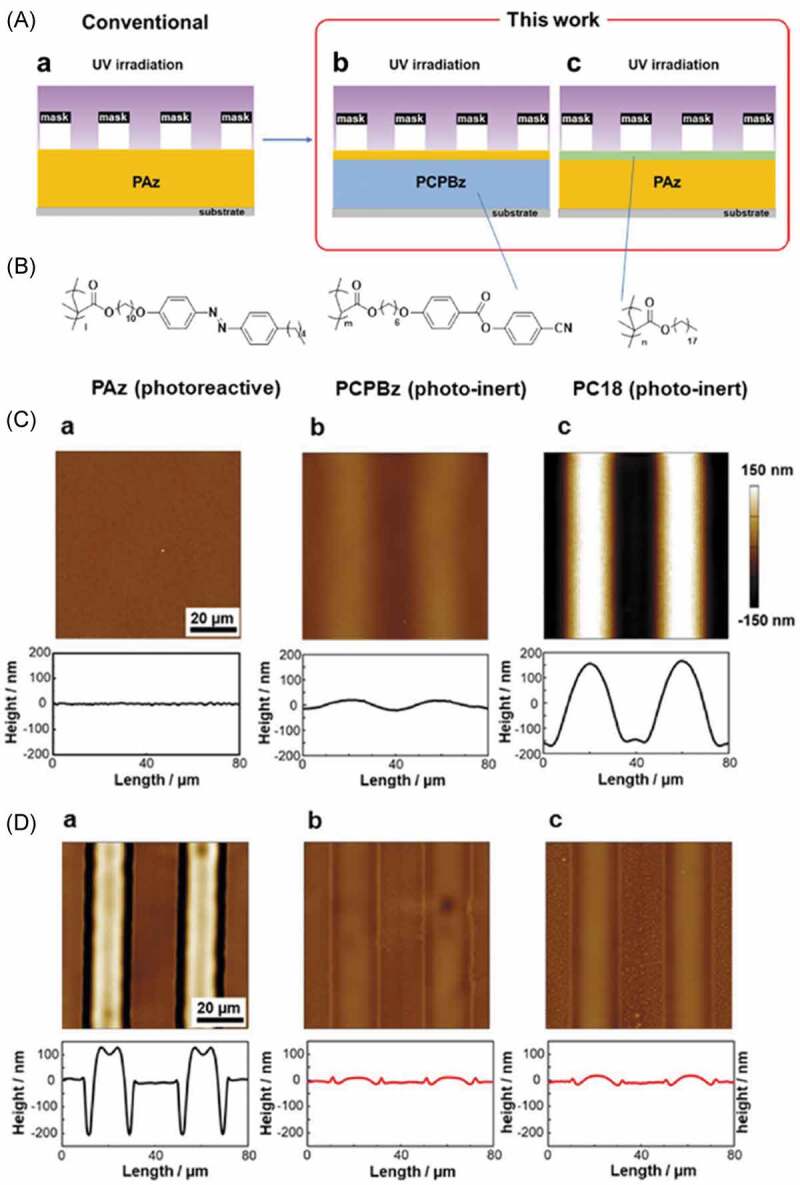
(A) Designed films for SRG (a) conventional and (b and c) designed in this study, (B) molecular structures of the polymers, (C) AFM images for SRG formation of (a) pure PCPBz film and PCPBz film with (b) 1 layer and (c) 10 layer PAz films and (D) AFM images for SRG formation of (a) pure PAz film and PAz film with (b) 1 layer and (c) 3 layer PC18 LC films on the surface. (Reproduced from the reference [314] with permission).

In order to clarify the effect of the surface tension on the SRG formation, SRG of a photoinactive polymer (PCPBz, the structure is given in Figure 17, (b)) film coated with AZ polymer (PAz, the structure is given in Figure 17, (b)) was studied [314]. Although the PCPBz film (the thickness of 200 nm) without the PAz coating did not give SRG by UV irradiation with a photomask as shown in Figure 17, (Ca), the PCPBz films with the PAz coating prepared by Langmuir-Schaefer (LS) method gave the SRG. Moreover, the formation of SRG of the PAz films was disturbed by poly(octadecyl methacrylate) (PC18, the structure is given in Figure 17, (b)) coating, suggesting that change of the surface tension was suppressed by the photoinactive PC18 coating (Figure 17, (d)). It was concluded that Marangoni flow was major driving force for the SRG formation in AZ polymers and further studies to establish a unified model which explains all SRG systems are expected. Based on the conclusion, studies on SRG of photoinactive polymers ionically interacted with AZ molecules were reported to show beneficial aspects of photoinduced surface tension difference with keeping high mobility of the components [237,278,315,324].

### 5.3. Structure variation of SRG

**Figure 18.**
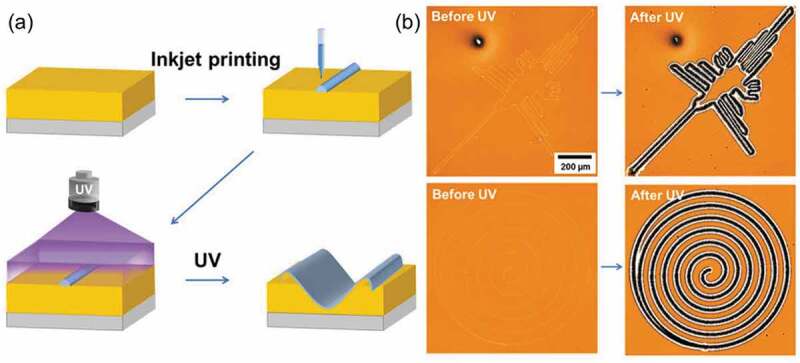
(a) Schematic drawing for the SRG formation by inkjet printing and (b) the SRG formation at inkjet printed line. (Reproduced from the reference [313] with permission).

Interfering laser and photomasks have been used to form grating structures, while several structures such as holes, needles, wells, spiral reliefs and lattice structures formed by controlling optical polarity [281], optical vortex [248,255] and shaping of irradiated area [212,269,281]. As shown in Figure 16(c), micrometer sized structures such as a truncated cone with the diameter of ca. 60 μm and a well with the diameter of 81 μm were obtained by the controlled light irradiation. Nanometer scale SRG whose size was same as the wavelength of visible light enabled to prepare holographic images [203,274,325–331]. By combining real time holography whose response time was within 200 ms [332–338], holography with a clearer image and quicker response time was suggested. Furthermore, inkjet printing technique led arbitrary shaped surface morphology by the SRG as shown in Figure 18 [313]. A block copolymer consisting of PAz (see Figure 17) and polybutyl methacrylate was printed on a PAz substrate (thickness: ca. 350 nm) by the inkjet printing with a sub-femtoliter droplet and was subsequently irradiated by UV light to form SRG with the shapes of Nazka Desert humming bird (Figure 18(b), upper) and a spiral circle (Figure 18(b), lower).

### 5.4. Movement of macroscopic objects

Not only for molecular species and colloidal particles, the movements of macroscopic objects are worth investigating even though the larger energy is required for the movement as the kinetic energy is expressed as a function of the weight of the object [339–342]. Surface tension difference [343–353] and resulting Marangoni flow [354,355] have been used to induce the movement of μm to cm sized objects such as liquid droplet, glass particles and molecular crystal [345,349–353]. In this section, photoinduced movement of the macroscopic size objects modified with photoresponsive surface tension control units is discussed.

**Figure 19.**
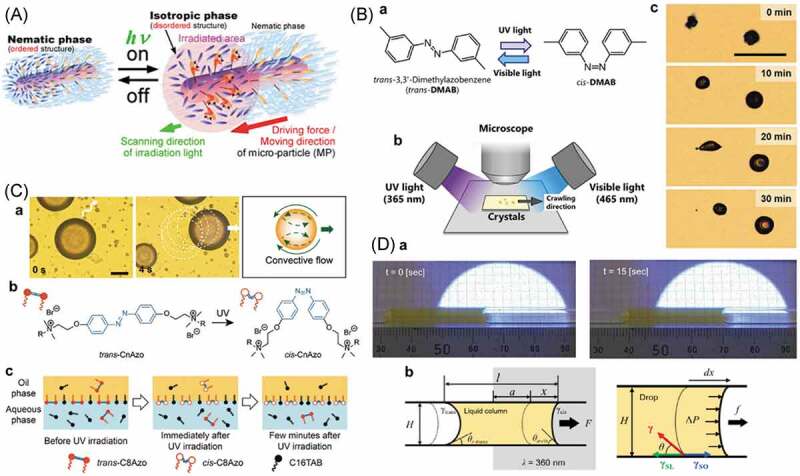
(A) Migration of AZ functionalized glass rod, (B) photochromism of DMAB (a), set-up to observe the movement of crystal of DMAB under simultaneous UV and visible light irradiation (b) and photographs for movement of DMAB crystals (c), (C) optical microscopy image and schematic illustration of droplet movement by the isomerization of AZ surfactant (a), molecular structure of CnAzo (b) and schematic illustration of proposed mechanism of the droplet movement (c) and (D) photographs of the movement of the AZ aqueous solution by the UV irradiation (a) and schematic illustration of proposed mechanism of the movement (b). (Reproduced from the references [343,346,355] and [347] with permission).

A rod-shaped glass particle (ca. 5 μm diameter × 24 μm length) functionalized with AZ moved to the light on a nematic liquid crystal film of 4-cyano-4′-pentyl-biphenyl (5CB) as illustrated in Figure 19(a) [343]. Before the irradiation, the *trans*-AZ groups aligned along the surrounding 5CB molecules, while by the irradiation, photogenerated *cis*-isomers caused the disordered alignment of 5CB and a phase transition to an isotropic phase. The phase transition was thought to induce the difference in the surface tension between the irradiated and the non-irradiated regions, being the driving force for the movements of the particle.

DMAB crystals (the molecular structure is given in Figure 19, (Ba)) moved by simultaneous UV and visible light irradiation [346]. The crystals became longer along the light irradiation during the movement to the visible light, suggesting the driving force for the movement was melting of the crystals by the UV irradiation to generate the *cis*-isomer and simultaneous recrystallization by the visible light irradiation [344,348]. The crystal moved by the difference in wettability in the melted and the crystalline regions

As shown in Figure 19, (Ca), a droplet of *n*-heptyloxybenzaldehyde in an aqueous solution of hexadecyltrimethylammonium bromide (CTAB) and CnAzo (the molecular structure is given in Figure 19, (Cb)) moved away from the UV light [355]. Before the UV irradiation, CTAB and *trans*-CnAzo being surrounded the oil droplet. The *cis*-isomer became the major isomer in the irradiated area and caused the change of surface tension and simultaneous Marangoni flow in the droplet (Figure 19, (Cb)). At the photostationary state, the migration was stopped as a result of the homogeneous surface tension.

An aqueous solution of an AZ surfactant moved to the light in the glass tube with the inner diameter of 2.5 mm as shown in Figure 19, (D) [347]. The surface tension of the irradiated region of the solution changed by the photogenerated *cis*-isomer to be a driving force for the movement [356].

### 5.5. Movement of macroscopic objects on photoresponsive surface

**Figure 20.**
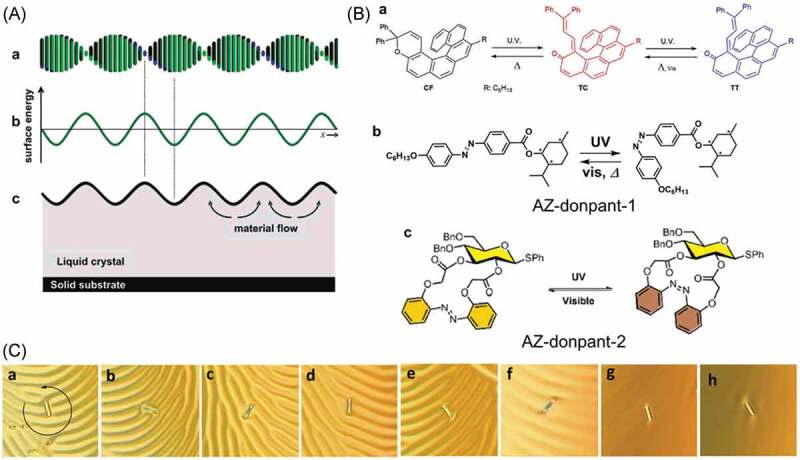
(A) Twisted alignment of ChLC (a), periodic surface energy of ChLC (b) and surface structure of ChLC (c), (B) molecular structures of photochromic chiral dopant discussed in this section and (C) rotation of a glass rod by photochromism of the naphthopyran (in Ba) in E7. (Reproduced from the references [357,360] and [369] with permission).

Micrometer sized glass rods and flakes rotated on the surface of chiral nematic liquid crystals, which are also known as cholesteric liquid crystals (ChLCs) [357–369]. ChLCs are prepared by adding chiral molecules to nematic liquid crystals and have a twisted alignment. On a glass substrate, ChLCs show characteristic fingerprint textures reflecting the twisted alignment as shown in Figure 20, (Aa) and (C). The pitch length was controlled by photoresponsive chiral dopants such as AZ [370] and DAE [371]. The photoinduced change in the pitch length caused the change in the surface energy (Figure 20, (Ab)) of ChLC, enabling the rotation of micrometer sized (30 μm) glass rods (Figure 20, (c)). A glass rod (average length: 25 μm, diameter: 5 μm) rotated almost 3 cycles (1150°) on a nematic liquid crystal E7 doped with a naphthopyran, which was functionalized with helicene (1 wt%, Figure 20, (Ba)) by the UV irradiation for 5 min [369]. Subsequently the glass rod retuned 206° in the dark. A nematic liquid crystal E44 containing AZ-dopant-1 (Figure 20, (Bb)) rotated a glass rod (length: ca. 30 μm, diameter: 7 μm) ca. 1200° by UV light irradiation [372]. By using the phase transition from a fingerprint texture to a focal conic state by the light irradiation, unidirectional rotation of a glass flake (size: several tens of μm) was achieved [360]. The finger print texture of AZ-dopant-2 (Figure 20, (Bc)) added 5CB (1.7 wt%) changed to the focal conic state by the UV irradiation without rotating the glass flake and the subsequent visible light irradiation led the focal conic state to the initial fingerprint texture with rotating the glass flake.

**Figure 21.**
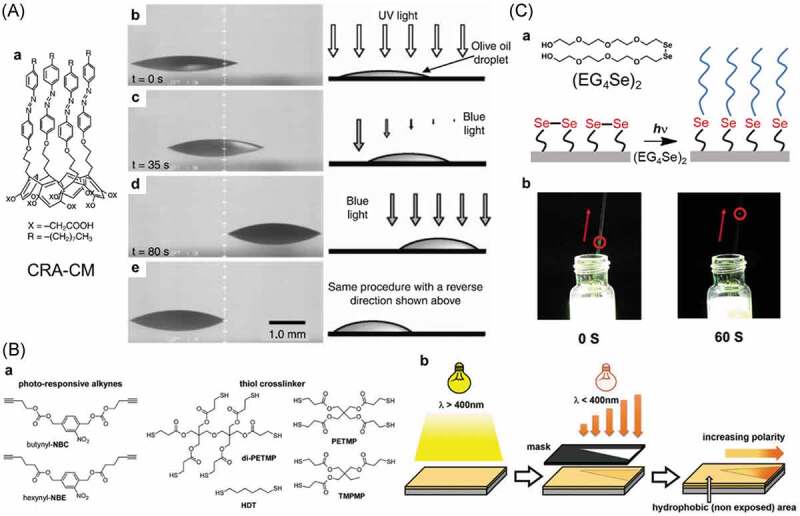
(A) Molecular structure of calixarene functionalized with AZ moieties (CRA-CM) (a) and lateral photographs of light-driven motion of an olive oil droplet on a silica plate modified with CRA-CM (b), (B) photoresponsive bifunctional alkynes and multifunctional thiols used for the preparation of thiol – yne networks with multigradient surface properties (a) and schematic representation of the photoinduced formation of a Laplace pressure gradient and a wettability gradient on thiol – yne photopolymer surfaces (b) and (C) exchange reaction of diselenide bonds on the surface of a capillary by (EG_4_Se)_2_ (a) and liquid motion was achieved by altering surface wettability inside the diselenide bond modified capillary tube (b). (Reproduced from the references [373,383] and [384] with permission).

Functionalization of substrate surface with AZ was done to impart switching function; the surface tension caused by the photoisomerization induced the movements of liquid droplet [373–379], PS sphere [380,381] and silica particles [382]. An olive oil droplet on a surface of a silica plate functionalized with CRA-CM (Figure 21, (Aa)) moved by light irradiation [373]. As shown in Figure 21, (Ab), the UV light was irradiated to the silica plate to prepare a *cis*-rich surface (a) and subsequent blue light irradiation made *trans*-rich (low surface energy) and *cis*-rich (high surface energy) regions. Consequently, the droplet transferred from the *trans*-rich (left) to the *cis*-rich regions (right) (b). Blue light irradiation to the substrate reset the surface state so that the droplet transferred opposite direction by the same protocol (d). Droplet with a molecular shuttle moved by the light irradiation, where the droplet climbed up 12° inclination [385].

Photodecomposition of surface components induced change in the wettability, which induced the movement of water droplets on the surfaces [383,386,387]. TiO_2_ particles on the surface of a poly(methyl methacrylate) (PMMA) film generated oxygen vacancies by UV irradiation, and the vacancies were used for the adsorption of water in air to form a hydrophilic surface [386]. The water contact angle decreased from 73° to 28° by the UV irradiation for 90 min. The difference in the surface wettability in the irradiated and the non-irradiated regions led the movement of the droplet to the irradiated region (more hydrophilic). A polymer shown in Figure 21, (Ba) synthesized from alkynes and thiol crosslinkers by visible light irradiation decomposed by subsequent UV irradiation. The photodecomposition of the polymer gave polar products such as carboxylates (Figure 21, (Bb) from middle to right) [383,387]. The asymmetric irradiation with a photomask resulted in a pattern, which led the water droplet to move to the irradiated area as a result of the hydrophilicity shown by the contact angle from 139° to 10° (Figure 21, (Bb) right).

Movement of liquid in tubes coated with diselenide bonds and AZ polymers was reported [384,388,389]. Diselenide bonds showed hemolytic bond cleavage by visible light irradiation or heat and recombined to the initial diselenide bonds, so that hetero dimers were obtained from homo dimers in the presence of some diselenide compounds [390–392]. The surface of a capillary (inner diameter of 0.5 mm) was functionalized with 3-aminopropyltriehyoxysilane and subsequent di(valeric acid) diselenide [384]. The capillary was put into an aqueous solution of (EG_4_Se)_2_ (Figure 21, (Ca)) and was irradiated by the visible light. The height of the solution in the capillary rose probably caused by the changing of surface wettability by the substitution of the surface selenide bonds with hydrophilic ethylene glycol units in (EG_4_Se)_2_ (Figure 21(C)). Such liquids as isopropanol, silicon oil and water moved away from the light in ethylene-vinyl acetate tube (inner diameter: 0.4 mm) coated with the AZ polymer by the irradiation shorter than 470 nm [388,389]. The difference in Laplace pressure in the irradiated and the non-irradiated regions was thought to be a driving force of the movement.

## 6. Photoinduced adsorption/desorption between liquid-solid

Adsorption/desorption on solids from solutions is important phenomenon connecting to the application for concentration, separation and purification especially for/from aqueous solutions. The desorption is a key for the slow or controlled release for agricultural and medical applications. Accordingly, photoresponsive adsorption/desorption is expected as advanced separation and controlled release systems.

### 6.1. Photoinduced adsorption/desorption of photochromic compounds

**Figure 22.**
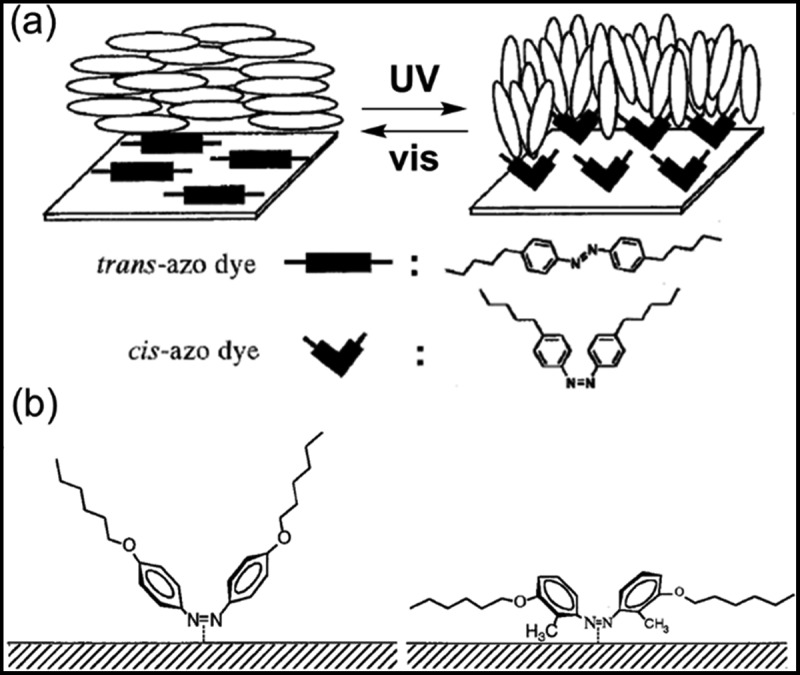
Schematic drawing of (a) photoinduced adsorption of AZ derivative with changing molecular orientation of liquid crystal and (b) adsorption of 4,4′-substituted and 3,3′-substituted AZ derivatives. (Reproduced from the reference [399] with permission).

Photochromic reactions accompany changes in the polarity, which has been used as a trigger for several photoinduced phenomena as introduced previously. It was reported that AZ derivatives such as methyl red induced change in the orientation of liquid crystals by UV irradiation [393–395]. The mechanism of the phenomena was explained as photoinduced adsorption of polar *cis*-isomers onto the substrates [396–398]. The non-polar *trans*-isomers followed the molecular orientation of the host nematic liquid crystals on the substrate, on the other hand, the polar *cis*-isomers were adsorbed on the substrate and caused the change in the molecular orientation as shown in Figure 22(a). The molecular structure of AZ derivatives affected the orientation of the liquid crystals [399–401]. As shown in Figure 22(b), a 4,4′-substituted AZ derivative was shown to induce the change in the orientation of a nematic liquid crystal, DON-103 (LODIC), from planar (parallel to the substrate) to homeotropic orientation (perpendicular to the substrate) by light irradiation [399]. It was explained that the substituents oriented perpendicular to the substrate. In contrast, the nematic liquid crystal changed its orientation from random planar (parallel to the substrate without preferred direction of the alignment) to uniform planer one (parallel to the substrate with one direction of the alignment) by photoisomerization of a 3,3′-substituted AZ derivative. The AZ derivative was adsorbed on the substrate with the molecular long axis parallel to the substrate and acted as a director of the alignment. The photoinduced adsorption of AZ derivatives caused the changes in the orientation of nematic liquid crystals from planar to homeotropic orientation [402–404], the rotation of the direction of cholesteric liquid crystal (ChLC) in planar orientation [405] and the phase transition from nematic to twisted nematic phases [402,406,407].

**Figure 23.**
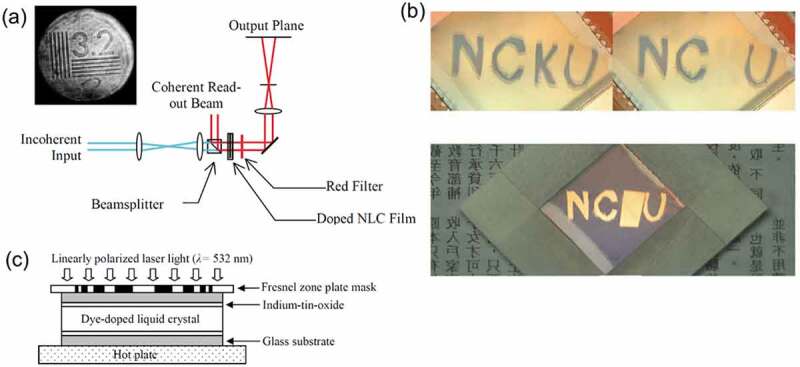
(a) Optical system to make holographic images and (inset) the resulting image, (b) example of display image of letters written by photoinduced adsorption (left top), subsequently erasing the latter of “K” (right top) and rewriting of a square shaped mark (bottom) and (c) a set up to prepare fresnel lens. (Reproduced from the references [413,414] and [415] with permission).

The changes of the surface tension and the refractive index caused by the photoinduced adsorption of AZ derivatives were used for making SRG [408–411] and holography [412]. A methyl red doped nematic liquid crystal, E7 (Merck), formed surface reliefs with 380 μm distance gratings by the interference fringes of He-Ne laser [408–411], which is regarded as a kind of SRG. By utilizing the refractive index change with the SRG formation, a holographic image was obtained as shown in Figure 23(a) [413,416]. Developments of display [414,417–419] and Fresnel lens [415,420,421] by photoinduced adsorption of methyl red were reported. As shown in Figure 23(b) left top, gray colored letters of ‘NCKU’ were written by 532 nm laser irradiation to E7 doped with methyl red [414]. The letters were written by the phase transition to twisted nematic phase. The letter of ‘K’ was erased by subsequent circularly polarized laser irradiation (right top). A square shaped mark was written instead of ‘K’ by the laser irradiation (bottom), where the bright and the dark parts were opposite due to a quarter-wave plate. By 532 nm irradiation to E7 doped with methyl red through a Fresnel zone plate mask (Figure 23(c)), Fresnel lens based on liquid crystal was prepared [415,420,421] with the first order diffraction efficiency up to 35% in the presence of addition of operating voltage at 3.6 V [421].

**Figure 24.**
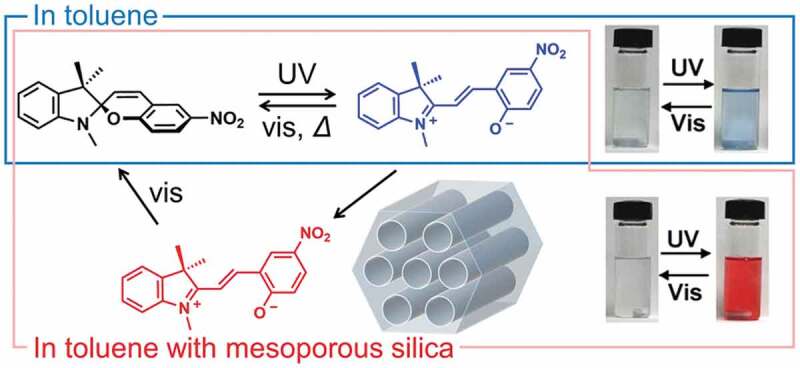
Photochromism of SP in toluene (blue square) and toluene with MPS (red square).

SP changed from colorless to blue by UV irradiation in toluene, while SP turned to red in the presence of mesoporous silicas (MPSs) as shown in Figure 24 [422]. The difference in color with/without the MPSs was thought to be due to the adsorption of the photogenerated MC. Adsorption kinetics were estimated by UV-vis absorption spectra depending on the irradiation time [423,424]. The changes of the concentration of SP followed *pseudo*-first or *pseudo*-second order kinetics and a MPS with larger pore (SBA-15 with 9.5 nm diameter) gave a larger *pseudo*-second adsorption rate constant (9.55 × 10^−3^ g(mg min)^−1^) than that with the smaller pore (MCM-41 with 2.2 nm diameter, 8.91 × 10^−3^ g(mg min)^−1^), suggesting that the diffusion of MC in the pore played an important role for the adsorption. The same phenomenon, photoinduced adsorption of MC, was reported in the presence of a clay mineral (a synthetic hectorite) [425,426]. Electrostatic interactions of MC with a negatively charged silicate layer and/or ion-dipole interactions with the interlayer cation were thought to be the driving forces. The effect of the interlayer cation on the photoinduced adsorption was examined by using Na^+^ and H^+^ intercalated hectorites [426]. The adsorption of MC onto the hectorites had two *pseudo*-first order constants attributed to the adsorption of MC and the protonated MC. The rate constant of the slower component for the Na^+^-hectorite (0.186 min^−1^) was larger than that (0.054 min^−1^) for H^+^-hectorite, suggesting that the importance of the interlayer cations on the photoinduced adsorption rate. The adsorbed amount of MC on H^+^-hectorite (8.75 mg/g) was larger than that (7.33 mg/g) on Na^+^-hectorite. Generation of the thermodynamically stable protonated MC instead of MC was thought to be a reason for the larger adsorbed amount seen for H^+^-hectorite [427].

### 6.2. Photoinduced uptake and release by photoresponsive vehicles

Adsorbent which shows controlled release is expected as vehicles to deliver the guest drugs into a target tissue in our body, which is known as drug delivery system (DDS). The light induces contactless release of the guest molecules only in the irradiated region.

#### 6.2.1. Release by photoinduced molecular vibration

**Figure 25.**
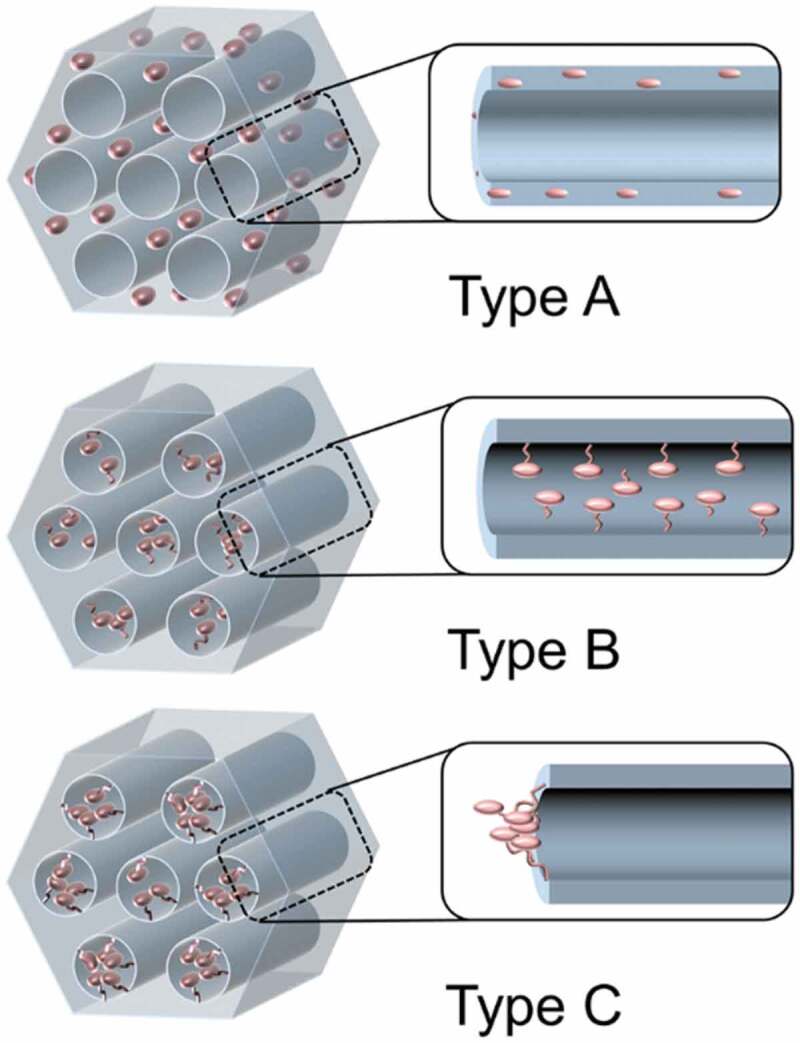
Schematic drawing for organic functionalization of MPSs (Type A) as framework backbone and grafted (Type B) in pores and (Type C) at orifices.

**Figure 26.**
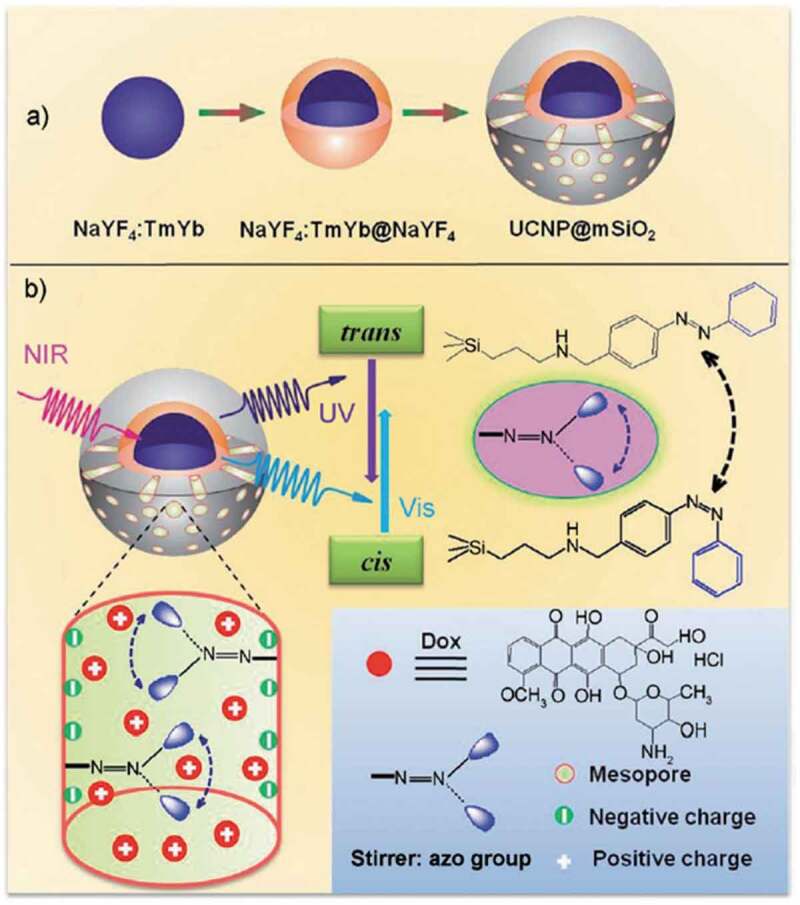
(a) Synthetic procedure for upconverting nanoparticles coated with a MPS outer layer and (b) NIR light-triggered doxorubicin release by making use of the upconversion property of the core-shell particle and *trans*–*cis* photoisomerization of AZ molecules grafted in the mesopore network of a MPS layer. (Reproduced from the reference [431] with permission).

Dyes such as fluorescein [428], rhodamine 6 G [429] and B [430], and propidium iodide [430] and drugs such as camptothecin [430] and doxorubicin [431] were released by photoinduced molecular vibration of AZ attached on MPSs with Type B functionalization shown in Figure 25. Near infrared (NIR) light triggered release of doxorubicin was reported by using an upconverting core-shell particle consisting of the core of NaYF_4_ containing 0.5 mol% Tm^3+^ and 20 mol% Yb^3+^ and the inner and external shells (inner shell: NaYF_4_ and external shell: Type B MPS functionalized with AZ moieties) as shown in Figure 26(a) [431]. Doxorubicin was released up to 80% from the MPS shell by 980 nm irradiation with 8.9 W/cm^2^ for 17 h, although the release was less than 5% in the dark. The efficient release by the irradiation was thought to be due to the molecular motion of AZ excited by 350 nm light upconverted in the core (Figure 26(b)). The light irradiation contributed to the release of the guest molecules through the induced molecular vibration.

#### 6.2.2. Release by photodecomposition

**Figure 27.**
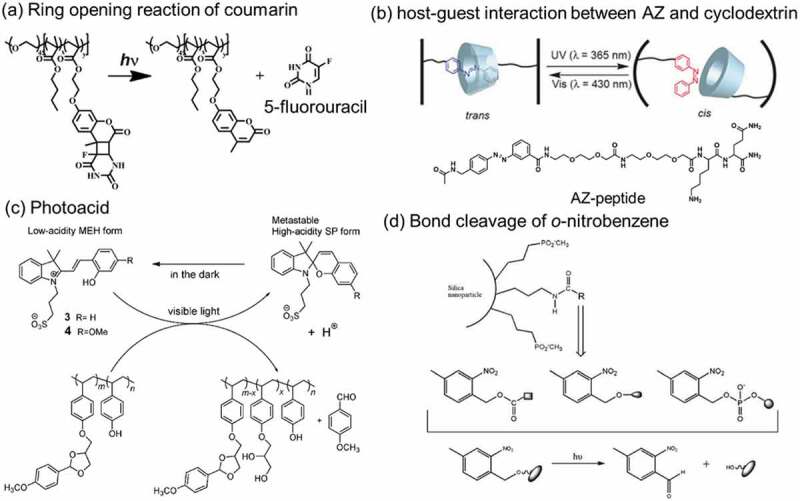
Photoreactions used for controlled release by (a) ring opening reaction of coumarin, (b) host-guest interaction between AZ and cyclodextrin, (c) photoacid and (d) bond cleavage of *o*-nitrobenzyl groups. (Reproduced from the references [188,435,436] and [443] with permission).

Nitrogen oxide [432], dyes such as AZ [433], Nile red [434] and anisaldehyde [435] and drugs such as 5-fluorouracil [436,437], camptothecin [438], doxorubicin [439–441] and bromoisophosphoramide [442] were released by the photodecomposition of polymers, MPS, Au nanoparticle and graphene oxide. As summarized in Figure 27, several strategies have been used for the photodecomposition via (a) ring-opening reaction of coumarin unit, (b) switching of host-guest interaction of AZ and cyclodextrin, and bond cleavage by (c) photoacid and activated oxygen, and of (d) *o*-nitrobenzil groups and diselenide bonds (Figure 21(C)). By using ring opening reaction of coumarins (Figure 27(a)), 5-fluorouracil was released from a block copolymer, poly(ethylene oxide) methyl ether and poly(*n*-butyl methacrylate), functionalized with coumarins [436]. Agglomerate of polyamidoamine (PAMAM) dendrimers was decomposed by the ring opening reaction of coumarin linkers with releasing of contained 5-fluorouracil and DNA [437]. By utilizing photoswitching of interactions of AZ with cyclodextrin as shown in Figure 27(b), a hydrogel of poly(ethylene glycol) functionalized *β*-cyclodextrin released AZ-peptide (molecular structure is in Figure 27(b)) by UV irradiation [433]. Anisaldehyde was released from poly(4-vinyl phenol) substituted with anisaldehyde through acetal by decomposition of the acetal by photoacid (SP) (Figure 27(c)) [435,443]. The bond cleavage of *o*-nitrobenzyl groups shown in Figure 27(d) [444] induced the release of camptothecin from self-assembled DNA particle [438], doxorubicin from DNA [440] and chitosan particle [439]. A semiconducting polymer consisted of cyclopenta[2,1-b:3,4-b’]dithiophene and benzo[c] [1,2,5]thiadiazole generated activated oxygen by light irradiation and the activated oxygen decomposed 2-nitroimidazole side chain of the polymer to release bromoisophosphoramide [442]. A polyphosphoester substituted with doxorubicin through thioketal and amide groups released doxorubicin by 660 nm irradiation [441]. Oxygen was activated by photoexcitation of the thioketal side chain and the activated oxygen decomposed the amide group to release doxorubicin [441].

Photodecomposition of hydrogels [445,446] and vesicle [447–450] resulted in the photoinduced release of the drugs such as doxorubicin and mitoxantrone, DNA and a protein (GFP). Two dextrans with *β*-cyclodextrin and AZ side chains connected each other by using host-guest interaction of *β*-cyclodextrin and AZ (Figure 27(b)) to form a hydrogel. The hydrogel showed gel-sol transition by UV irradiation with releasing encapsulated GFP [445]. Photoisomerization of AZ incorporated in vesicles of liposome [447], sodium dodecylbenzene sulfonate (SDBS) [448], sodium dodecyl sulfate (SDS) [448] and pillar [6]arene [449] induced change in critical aggregation concentrations of the vesicles by UV irradiation [450]. Liposome incorporating a cholesterol functionalized AZ released encapsulated calcein by UV irradiation and the release was stopped by subsequent visible light irradiation [447].

#### 6.2.3. Release by photoinduced structure change of host vehicles

**Figure 28.**
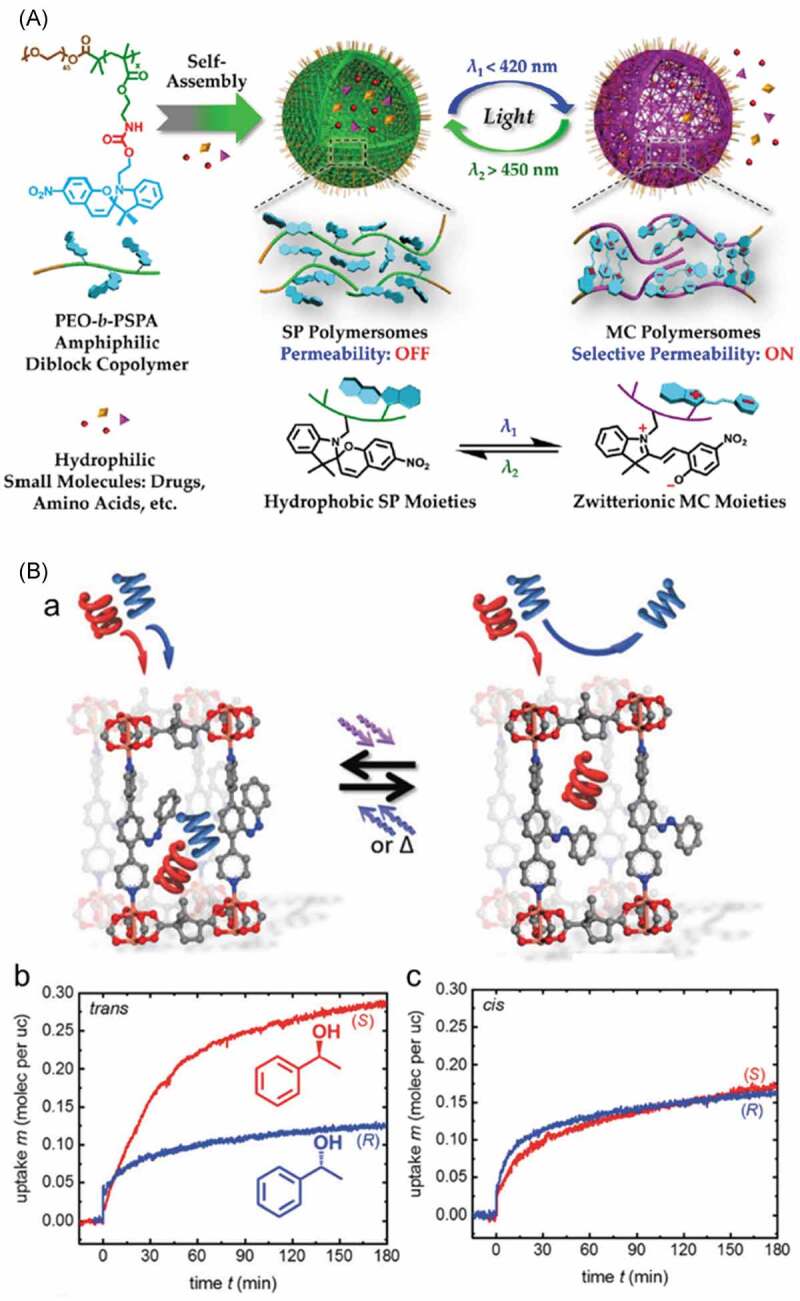
(A) Schematic representation of release of 2′-deoxy-5-fluorouracil and dextran from vesicle of PEO-*b*-PSPA by photogeneration of polar MC and (B) photoswitching of preferential adsorption of (*S*)-phenyl ethanol compared to (*R*)-phenyl ethanol (a) in the presence of *trans*-AZ (b) and *cis*-AZ ligands (c). (Reproduced from the references [453] and [459] with permission).

Isomerization of photochromic compounds such as AZ and SP induced structural changes of the hosts such as polymers, MPS and MOF, which accompanied the changes in the polarity [451–461], pore size [462–466] and switching of open/close of orifice of the vehicles [467–482]. The polarity change in polymers by the photochromism of AZ and SP was used to release butanediol [455], dyes such as fluorescein [456], methylene blue [457], Nile red [458] and coumarin [460], drugs such as 2′-deoxy-5-fluorouracil [459], dextran [459] and doxorubicin [460] and proteins such as lysozyme [452,454], chymotripsinogen [454] and chymotripsin [454]. Vesicles composed of PEO-*b*-PSPA and SP units (molecular structure is shown in Figure 28, (A)) were used as vehicles, which release 2′-deoxy-5-fluorouracil and dextran by UV irradiation [459]. Before the irradiation, the hydrophobic SP blocked the release of the hydrophilic guests in the vesicle, while the photogenerated hydrophilic MC allowed the release of the guests from the vesicle. The release was terminated in the dark due to the back reaction of MC to SP. An opposite phenomenon, photoinduced adsorption, was also achieved by the vesicle of PEO-*b*-PSPA with SP. Cysteine grafted Au nano particles was adsorbed to the vesicle by the UV irradiation. A Type A MOF substituted with AZ shown in Figure 28, (Ba) (and the category is in Figure 1) with the chemical composition of Cu_2_(DCam)_2_(AzoBiPyB) where DCam: D-camphoric acid and AzoBiPyB: (*E*)-2-(phenyldiazenyl)-1,4-bis(4-pyridyl)benzene) showed preferential adsorption of (*S*)-phenyl ethanol compared to (*R*)-phenyl ethanol in the presence of *trans*-AzoBiPyB (Figure 28, (Bb)) [453]. As the adsorbed amount of (*S*)- and that of (*R*)-phenyl ethanol with *cis*-AzoBiPyB was almost same as shown in Figure 28, (Bc), possible photoinduced chiral separation was proposed.

**Figure 29.**
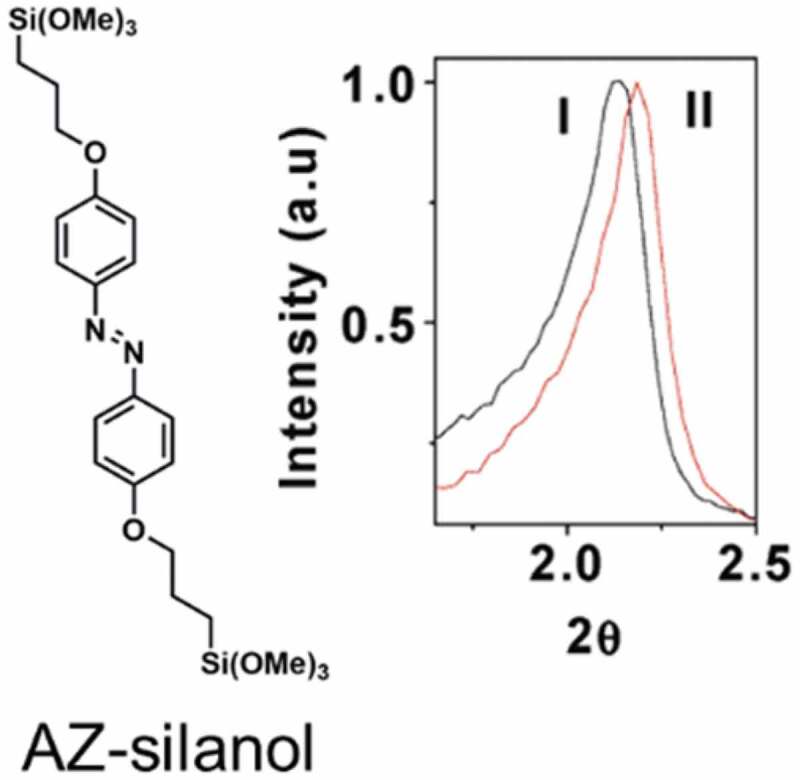
Molecular structure of AZ-silanol and change in the XRD pattern (I) before and (II) after UV irradiation. (Reproduced from the reference [462] with permission).

Pore size of MPS and MOF has been controlled by photochromism of AZ and coumarin, which enabled photoinduced release of propidium [463], butanediol [464,465], U(VI) [466] and Au nanoparticles [462]. A Type A MPS (category is shown in Figure 25) was synthesized from tetraethoxysilane (TEOS) and AZ-silanol (shown in Figure 29) in the presence of cetyltrimethylammonium bromide (CTAB), and was used for the collection of Au nanoparticles [462]. The efficiency shown by the removal of Au nanoparticles with the diameter less than 2.6 nm was different before (83%) and after the UV irradiation (64%). Change in the XRD pattern shown in Figure 29 suggested the pore size difference in the *trans*-rich and the *cis*-rich conditions achieved by the UV irradiation.

**Figure 30.**
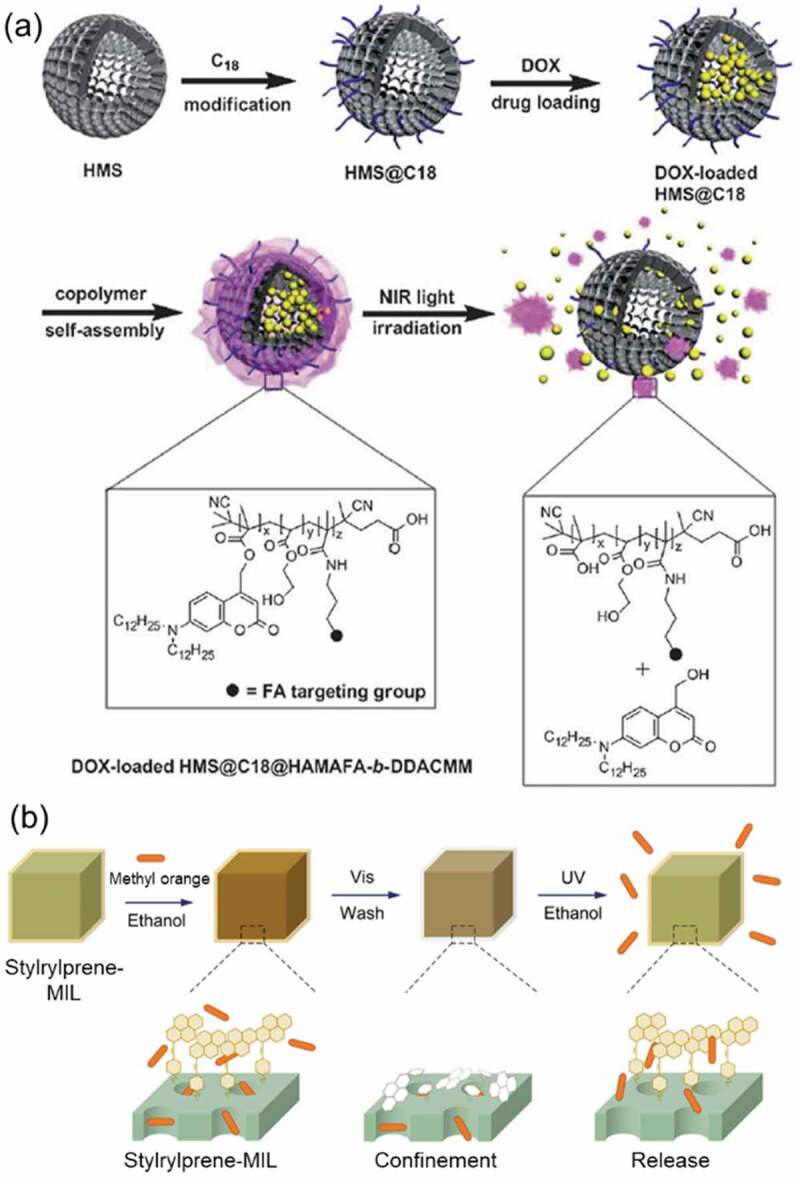
(a) Release of doxorubicin by two photon absorption and (b) encapsulation and subsequent release of methyl orange from SB (stylrylpyrene) grafted MIL-53. (Reproduced from the references [474] and [476] with permission).

Reversible opening and closing of orifices of MPSs [483] and MOFs [484] by light irradiation were used for the controlled release of butanediol [471], dyes such as pyrene [473], phenanthrene [468], methylene blue [481], methyl orange [474], brilliant blue [481], fluorescein [469,480,482] and rhodamine B [472], drugs such as cholestane [467], cholesterol [475], doxorubicin [470,476,479] and curcumin [478], and DNA [477]. Type B MPSs (category is shown in Figure 25) were prepared by substitution with photochromic units such as AZ [477–481], SP [469,470,482], coumarin [467,468,475,476] and SB [474]. The first example of this concept was reported in a MPS (MCM-41) functionalized with coumarin [467]. The orifice of Type B MPSs grafted with AZ was opened by photoswitching of interactions with cyclodextrins [478–480] and DNA [477] which acted as cap of the orifices. The orifice of Type B MPSs reversibly opened and closed by photoswitching of the polarity caused by the photochromism of the SP at the orifice [469,470,482]. Hydrophilic (polar) guests were not able to pass the orifice substituted with hydrophobic SP, while hydrophilic MC which was formed by UV irradiation allowed them to be released. Phenanthrene [468], cholestane [467] and cholesterol [475] were released by the ring opening reaction of coumarin dimers by 250 nm irradiation. As shown in Figure 30(a), an external surface of a hollow MPS particle synthesized with polystyrene (PS) latex template was grafted with octadecyltrimethoxysilane and subsequently coated with HAMAFA-*b*-DDACMM incorporating coumarin unit [476]. The polymer, HAMAFA-*b*-DDACMM, showed two photon absorption at 800 nm by the coumarin units, which caused the hydrolysis of the ester moieties. The decomposition induced the release of the polymer from the MPS and opened the pore of the MPS to release the loaded doxorubicin.

Orifice of MOF was opened and closed by the photochromism of the attached AZ [471,472] and SB [474]. A MOF, Cu_2_(BPDC)_2_(BiPy) (BPDC: biphenyl-4,4′-dicarboxylic acid, BiPy: 4,4′-bipyridine), whose external surfaces were grafted with AZ, induced a change in the pore size at the external surface by UV irradiation [471]. The change in the pore size was used as a trigger to release preloaded butanediol. Rhodamine B was loaded to a Type A MOF, which was consisted of zirconia cluster node and a terphenyl ligand substituted with *trans*-AZ side chain (ZrO(2′-ptolyldiazenyl-1,1′:4,4′-terphenyl-4,4′′-dicarboxylic acid)). In order to confine the rhodamine B, the orifice of the MOF was blocked by the complexation of *β*-cyclodextrin with the surface AZ side chain of the ligand of the MOF surface [471]. The loaded rhodamine B were released from the MOF by open of the orifice, which was induced by the desorption of *β*-cyclodextrin by the photoisomerization of the AZ side chain. The external surface of a MOF, MIL-53, was grafted with SB units (stylrylpyrene) as shown in Figure 30(b) [474]. The comparable pore size of MIL-53 (8.5 Å) and molecular size of the SB units (7.8 Å × 18.8 Å) allowed the dimerization of the SB units in the pore by visible light irradiation. As schematically shown in Figure 30(b), methyl orange was encapsulated in the SB grafted MIL-53 and immobilized by the visible light induced dimerization of the SB units. The backward reaction of the dimer to the monomer was induced by UV irradiation, leading the release of the methyl orange.

### 6.3. Movements of macroscopic objects

Camphor boat moves on water surface by surface tension difference between the front and the back of the boat generated by the sublimation of the attached camphor, which is a known example of self-propelled objects or moving objects driven by chemical energy. The movement of a camphor boat was controlled by using molecular layer of photochromic compounds (AZ [485] and hexaarylbiimidazole (HABI) [486]) spread on water surface. A camphor boat moved on the water surface covered with *trans*-4-[[(dodecyloxy)benz-4-yl]azo]benzoic acid and the movement was stopped by UV irradiation as a result of the surface pressure change [485]. Photoinduced movements of benzoquinone crystal (disk) [487] and 4-methoxyazobenzene (4-OMe-AZ) crystal [488] were reported. The 4-OMe-AZ crystal moved on the water surface by the dissolution of the photogenerated *cis*-isomer, which has higher solubility than that of the *trans*-isomer [489]. The movement of benzoquinone disk on a hydroquinone aqueous solution by the dissolution of benzoquinone was stopped by UV irradiation because the UV irradiation caused photoreaction of benzoquinone around the disk to uniformize the concentration gradient of hydroquinone and benzoquinone [487].

**Figure 31.**
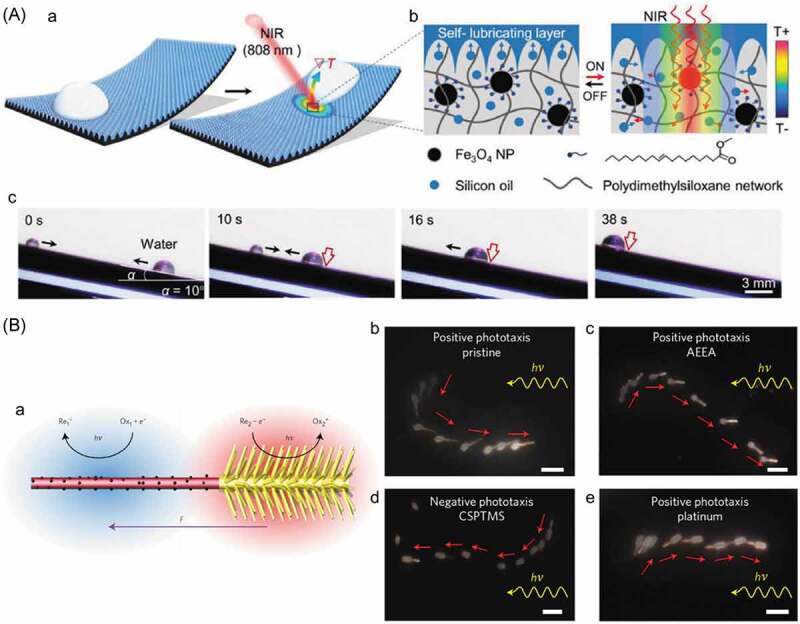
Schematic drawings of (A) migration of water droplet on photothermal surface of polydimethylsiloxane (PDMS) embedded Fe_3_O_4_ nanoparticles. (B) Janus type nanorod, where TiO_2_ nanowires (yellow) was grown on a silicon nanowire (pink) and platinum nanoparticles (black) and photocatalytic reaction on the surface (a), photoinduced motion of the nanorod (b and c) with positively charged surface, (d) positively charged head and negatively charged tail and (e) negatively charged surface. (Reproduced from the references [495] and [496] with permission).

The local heat was generated at the irradiated region owing to the thermal relaxation from the excited sate (photothermal effect). The heat caused the changes in the physicochemical properties of the irradiated region, which was used to drive a liquid flow [354,490,491]. Au coated poly(allylaminehydrochloride)/poly(styrenesulfonic acid) [492] and a MPS (MCM-41) [493] moved in water by light irradiation driven by the heat generated on the Au particle. An oil droplet coated with polyaniline moved away from the light on a water surface [494], which was explained as the heat generated in the irradiated region in polyaniline diffused on the water surface to cause Marangoni flow in the water. As shown in Figure 31, (A), such liquid droplets as water, glycerol and propylene glycol moved on the surface of polydimethylsiloxane (PDMS) plate embedded Fe_3_O_4_ nanoparticles. The heat caused by 808 nm irradiation to Fe_3_O_4_ induced Marangoni flow to the droplets on the PDMS surface. Consequently, the droplet moved away from the light and climbed up the hill of 10° inclination as shown in Figure 31(Ac) [495,497]. Photothermal effect of Au nanorods (length: 800 nm) on a glass substrate caused transformation of poly(*N*–isopropylacrylamide) coating, which thermotropically transformed from hydrophilic to hydrophobic at around 32°C [498]. The transformation caused the surface energy difference around the irradiated region, which induced the movement of a water droplet from hydrophobic (irradiated) to hydrophilic (non-irradiated) regions.

Movements of microscopic objects were also induced by concentration gradient generated by photocatalytic reactions [340]. A TiO_2_ particle and a Janus particle of a silica particle coated with Pt moved away from the UV irradiation [499,500]. The photothermal effect and the generation of O_2_ gas by the photocatalytic reaction on TiO_2_ and Pt were thought to drive the movement. A Janus nano rod, which had a silicon nano rod core (ca. 10 μm length) grafted with TiO_2_ nanowires and Pt nanoparticles as illustrated in Figure 31, (Ba), showed the surface charge dependent motion in water, which was induced by the photodecomposition of oxygen peroxide in the water [496]. The motion was controlled by the surface charge of the Janus rod and the external electric field caused by H^+^ and OH^−^ which were the products of the photocatalytic reactions by TiO_2_ and Pt, respectively. Positive phototaxis was observed when the rod had negative or positive surfaces (a, b and d), while negative phototaxis was observed when the two parts had opposite charges (c). Movement of water in tubes by photothermal effect was reported [501–504]. A tube of poly(*N*–isopropylacrylamide) whose external surface was coated with graphene oxide was used to shown the movement of water to the light [501]. The photothermal effect on graphene oxide induced the transformation of poly(*N*–isopropylacrylamide) from hydrophilic to hydrophobic, which accompanied increase of the inner diameter of the tube. The hydrophobic surface in the tube with larger diameter induced the movement of water away from the light. The photoinduced transformation of the tube surface character is expected to be used for optical actuator [505,506].

### 6.4. Directional movement of macroscopic object

**Figure 32.**
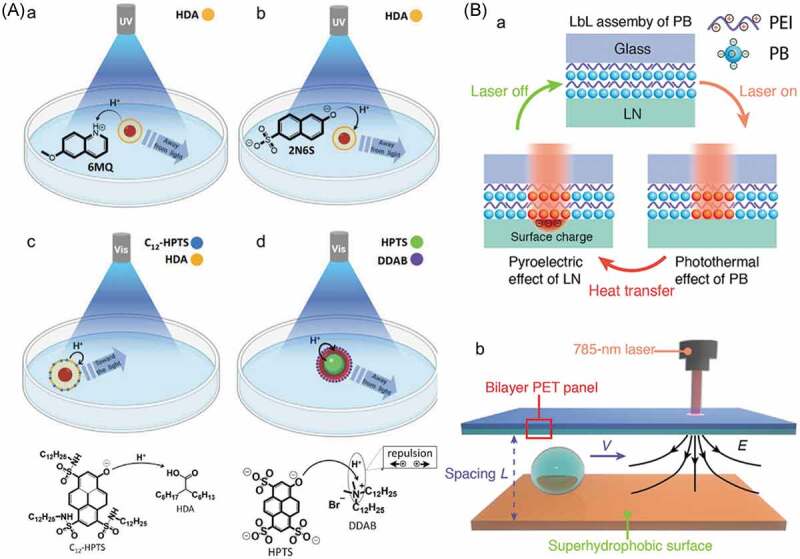
(A) Migration of nitrobenzene droplets surrounded with HDA (a) in an aqueous 6MQ solution, (b) in an aqueous 2N6S solution, (c) surrounded HDA and C_12_-HPTS and (d) surrounded DDAB and HPTS, and (B) by pyroelectric effected of lithium niobate caused by photothermal effect by Prussian blue. (Reproduced from the references [489] and [507] with permission).

Both positive and negative phototaxis was observed in photoinduced transfer of nitrobenzene droplets in water, depending on surfactants surrounded the nitrobenzene droplet and photoacid/photobase in the water phase as shown in Figure 32 [489]. A nitrobenzene droplet being surrounded with 2-hexyldecanoic acid (HDA) showed negative phototaxis in an aqueous solution of a photobase, 6-methoxyquinoline (6MQ, Figure 32(Aa)), or a photoacid, 2-naphthol-6-sulfonate (2N6S, Figure 32(Ab)). A droplet being surrounded with HDA and C_12_-HPTS (shown in Figure 32(Ac)) showed positive phototaxis while a water/oil/water vesicle composed of didodecyldimethylammonium bromide (DDAB) and 8-hydroxypyrene-1,3,6-trisulfonic acid (HPTS, shown in Figure 32(d)) showed negative phototaxis. In the aqueous solution of the photobase (6MQ, Figure 32(a)), 6MQ captured a proton from HDA to form deprotonated HDA (DA^−^) and non-reacted HDA diffused to the light to cause Marangoni flow away from the light. As schematically shown in Figure 32(Ab), the droplet was surrounded with DA^−^ initially. The DA^−^ accepted proton from the photoacid (2N6S), consequently the DA^−^ diffused to the irradiated region to make Marangoni flow away from the light. Photoinduced proton transfer from C_12_-HPTS to DA^−^ made flow of deprotonated C_12_-HPTS and HDA away from the light (Figure 32(Ac)), which induced the Marangoni flow to the light. In the water/oil/water vesicle shown in Figure 32(Ad), protons generated from photoacid changed the ion strength in the irradiated area in the water phase, which caused flow in the water phases to the light, as the result, the vesicle moved away from the light.

Pyroelectric effect of lithium niobate was induced by photothermal effect to provide surface charge on lithium niobate, enabling transfer of a water droplet along the electric field [507,508]. As shown in Figure 32(Ba), the glass substrate was coated with polyethylenimine (PEI) and Prussian blue (PB) and subsequently lithium niobate, which generated surface positive charges by the photothermal effect of PB [507]. The water droplet transferred on superhydrophobic surface of silver nanoparticles to the light irradiation (Figure 32(Bb)). The photoinduced movement of the droplet by the pyroelectric effect was also reported on a superhydrophobic surface of silica nanoparticles on a lithium niobate coated polydimethylsiloxane plate doped with graphene [508]. Surface positive charges were generated on the lithium niobate coating due to the photothermal effect of the graphene. Such liquid droplets as silicon oil, ethanol, *n*-heptane, glycerol and water migrated to the surface charge generated on the surface.

## 7. Conclusions

Inspired by ‘smart’ and ‘sustainable’ natural photoinduced phenomena in bio and geospheres, artificial systems from nano to micrometer scales have been designed to reveal movements of various objects upon photoirradiation. Photoresponsive molecules have been built from photochromic molecules and molecular linkers allowing binding to the target. They have been attached on the surface of particle, and incorporated or dispersed in polymers. Moving objects have been extended from molecules/ions to macroscopic particles. The movement of larger objects needs more energy, which was provided by photochemical and photothermal reactions. This combination of material driving force and system analysis allowed to create unique moving platforms; the movement direction in some of them can be controlled as an ‘artificial’ phototaxis. Driven by developments in the material design and the characterization (high-speed cameras, microscopes, positioning devises, etc.), the movements will be extended to improve the speed, direction, and response time of moving objects for applications in medical and other fields like molecular robot and actuators.

## References

[1] Bendix SW. Phototaxis. Bot Rev. 1960;26(2):145–208.

[2] Jékely G. Evolution of phototaxis. Philos Trans R Soc B Biol Sci. 2009;364(1531):2795–2808. 10.1098/rstb.2009.0072 PMC2781859 19720645

[3] Najafpour M, editor. Advances in photosynthesis - fundamental aspects. London: InTech; 2012.

[4] Kohen E, Santus R, Hirschberg J. Photobiology. 1st ed. London: Academic Press; 1995.

[5] Li Q, editor. Intelligent stimuli-responsive materials. Hoboken, NJ, USA: John Wiley & Sons, Inc.; 2013.

[6] White TJ, editor. Photomechanical materials, composites, and systems. Chichester, UK: John Wiley & Sons, Ltd; 2017.

[7] Shinkai S, Nakaji T, Ogawa T, et al. Photoresponsive crown ethers. 2. Photocontrol of ion extraction and ion transport by a bis(crown ether) with a butterfly-like motion. J Am Chem Soc. 1981;103(1):111–115. DOI:10.1021/ja00391a021

[8] Bouas-Laurent H, Dürr H. Organic photochromism (IUPAC technical report). Pure Appl Chem. 2001;73(4):639–665.

[9] Bullock DJW, Cumper CWN, Vogel AI. 989. Physical properties and chemical constitution. Part XLIII.* the electric dipole moments of azobenzene, azopyridine, and azoquinolines. J Chem Soc. 1965;5316–5323. DOI:10.1039/jr9650005316

[10] Ye Y, Pang J, Zhou X, et al. Understanding the torsion effects on optical properties of azobenzene derivatives. Comput Theory Chem. 2016;1076:17–22.

[11] Weissberger A. Dipole moment and structure of organic compounds. XVII.^1^ the electric moments of *α*- and *β*-stilbene dibromide and of *p*-diacetylbenzene. J Am Chem Soc. 1945;67(5):778–779.

[12] Jakobsson FLE, Marsal P, Braun S, et al. Tuning the energy levels of photochromic diarylethene compounds for opto-electronic switch devices. J Phys Chem C. 2009;113(42):18396–18405. DOI:10.1021/jp9043573

[13] Bletz M, Pfeifer-Fukumura U, Kolb U, et al. Ground-and first-excited-singlet-state electric dipole moments of some photochromic spirobenzopyrans in their spiropyran and merocyanine form. J Phys Chem A. 2002;106(10):2232–2236. DOI:10.1021/jp012562q

[14] Irie M, Fukaminato T, Matsuda K, et al. Photochromism of diarylethene molecules and crystals: memories, switches, and actuators. Chem Rev. 2014;114(24):12174–12277. DOI:10.1021/cr500249p 25514509

[15] Inouye M, Ueno M, Kitao T. Alkali metal recognition induced isomerization of spiropyrans. J Am Chem Soc. 1990;112(24):8977–8979.

[16] Tanaka M, Ikeda T, Xu Q, et al. Synthesis and photochromism of spirobenzopyrans and spirobenzothiapyran derivatives bearing monoazathiacrown ethers and noncyclic analogues in the presence of metal ions. J Org Chem. 2002;67(7):2223–2227. DOI:10.1021/jo0162843 11925232

[17] Heller CA, Fine DA, RA H. Photochromism. J Phys Chem. 1961;65(10):1908–1909.

[18] Irie M. Diarylethenes for memories and switches. Chem Rev. 2000;100(5):1685–1716. 11777416 10.1021/cr980069d

[19] Kumar GS, Neckers DC. Photochemistry of azobenzene-containing polymers. Chem Rev. 1989;89(8):1915–1925.

[20] Peng L, You M, Wu C, et al. Reversible Phase transfer of nanoparticles based on photoswitchable host–guest chemistry. ACS Nano. 2014;8(3):2555–2561. DOI:10.1021/nn4061385 24524295 PMC4004314

[21] Yamaguchi T, Ogawa M. Photochromic reactions in nanospace; host-guest interactions and opportunity. In: Douhal A, and Anpo M, editors. Dye photoactive mol microporous syst. 1st ed. Amsterdam: Elsevier; 2020. pp. 163–177.

[22] In: Nakato T, Kawamata J, Takagi S editors. Inorganic nanosheets and nanosheet-based materials. Tokyo: Springer Japan; 2017.

[23] Zhu J, Ding JJ, Liu XQ, et al. Realizing both selective adsorption and efficient regeneration using adsorbents with photo-regulated molecular gates. Chem Commun. 2016;52(21):4006–4009. DOI:10.1039/C5CC10634F 26864289

[24] Cheng L, Jiang Y, Qi SC, et al. Controllable adsorption of CO_2_ on smart adsorbents: an interplay between amines and photoresponsive molecules. Chem Mater. 2018;30(10):3429–3437. DOI:10.1021/acs.chemmater.8b01005

[25] Jiang Y, Park J, Tan P, et al. Maximizing photoresponsive efficiency by isolating metal–organic polyhedra into confined nanoscaled spaces. J Am Chem Soc. 2020;141(20):8221–8227. DOI:10.1021/jacs.9b01380 30983347

[26] Baroncini M, D’Agostino S, Bergamini G, et al. Photoinduced reversible switching of porosity in molecular crystals based on star-shaped azobenzene tetramers. Nat Chem. 2015;7(8):634–640. DOI:10.1038/nchem.2304 26201739

[27] Huang N, Ding X, Kim J, et al. A Photoresponsive smart covalent organic framework. Angew Chem Int Ed. 2015;54:8704–8707. 10.1002/anie.201503902 PMC4531826 26095503

[28] Getman RB, Bae YS, Wilmer CE, et al. Review and analysis of molecular simulations of methane, hydrogen, and acetylene storage in metal–organic frameworks. Chem Rev. 2012;112(2):703–723. DOI:10.1021/cr200217c 22188435

[29] Bétard A, Fischer RA. Metal–organic framework thin films: from fundamentals to applications. Chem Rev. 2012;112(2):1055–1083. 21928861 10.1021/cr200167v

[30] Dolgopolova EA, Rice AM, Martin CR, et al. Photochemistry and photophysics of MOFs: steps towards MOF-based sensing enhancements. Chem Soc Rev. 2018;47(13):4710–4728. DOI:10.1039/C7CS00861A 29546889

[31] Rice AM, Martin CR, Galitskiy VA, et al. Photophysics modulation in photoswitchable metal–organic frameworks. Chem Rev. 2020;120(16):8790–8813. DOI:10.1021/acs.chemrev.9b00350 31638383

[32] Hosono N, Uemura T. Metal–organic frameworks as versatile media for polymer adsorption and separation. Acc Chem Res. 2021;54(18):3593–3603. 34506124 10.1021/acs.accounts.1c00377

[33] Le Ouay B, Watanabe C, Mochizuki S, et al. Selective sorting of polymers with different terminal groups using metal-organic frameworks. Nat Commun. 2018;9(1):3635. DOI:10.1038/s41467-018-06099-z 30194388 PMC6128874

[34] Yao QX, Ju ZF, Jin XH, et al. Novel polythreaded coordination polymer: from an armed-polyrotaxane sheet to a 3D polypseudorotaxane array, photo-thermochromic behaviors. Inorg Chem. 2009;48(4):1266–1268. DOI:10.1021/ic8021672 19166285

[35] Modrow A, Zargarani D, Herges R, et al. The first porous MOF with photoswitchable linker molecules. Dalton Trans. 2011;40(16):4217–4222. DOI:10.1039/c0dt01629b 21394353

[36] Jin CM, Zhu Z, Chen ZF, et al. An unusual three-dimensional water cluster in metal−organic frameworks based on ZnX_2_ (X = ClO_4_, BF_4_) and an azo-functional ligand. Cryst Growth Des. 2010;10(5):2054–2056. DOI:10.1021/cg100226u

[37] Liu Y, Eubank JF, Cairns AJ, et al. Assembly of metal–organic frameworks (MOFs) based on indium-trimer building blocks: a porous MOF with soc topology and high hydrogen storage. Angew Chem Int Ed. 2007;46(18):3278–3283. DOI:10.1002/anie.200604306 17385775

[38] Sato H, Matsuda R, Sugimoto K, et al. Photoactivation of a nanoporous crystal for on-demand guest trapping and conversion. Nat Mater. 2010;9(8):661–666. DOI:10.1038/nmat2808 20651806

[39] Yanai N, Uemura T, Inoue M, et al. Guest-to-host transmission of structural changes for stimuli-responsive adsorption property. J Am Chem Soc. 2012;134(10):4501–4504. DOI:10.1021/ja2115713 22372403

[40] Castellanos S, Goulet-Hanssens A, Zhao F, et al. Structural effects in visible-light-responsive metal-organic frameworks incorporating ortho-fluoroazobenzenes. Chem - A Eur J. 2016;22(2):746–752. DOI:10.1002/chem.201503503 26617393

[41] Prasetya N, Donose BC, Ladewig BP. A new and highly robust light-responsive Azo-UiO-66 for highly selective and low energy post-combustion CO_2_ capture and its application in a mixed matrix membrane for CO_2_/N_2_ separation. J Mater Chem A. 2018;6(34):16390–16402.

[42] Modrow A, Zargarani D, Herges R, et al. Introducing a photo-switchable azo-functionality inside Cr-MIL-101-NH_2_ by covalent post-synthetic modification. Dalton Trans. 2012;41(28):8690–8696. DOI:10.1039/c2dt30672g 22692132

[43] Park J, Yuan D, Pham KT, et al. Reversible alteration of CO_2_ adsorption upon photochemical or thermal treatment in a metal–organic framework. J Am Chem Soc. 2012;134(1):99–102. DOI:10.1021/ja209197f 22148550

[44] Jiang Y, Shi XC, Tan P, et al. Controllable CO_2_ capture in metal–organic frameworks: making targeted active sites respond to light. Ind Eng Chem Res. 2020;59(50):21894–21900. DOI:10.1021/acs.iecr.0c04126

[45] Huang H, Sato H, Aida T. Crystalline nanochannels with pendant azobenzene groups: steric or polar effects on gas adsorption and diffusion? J Am Chem Soc. 2017;139(26):8784–8787. 28635263 10.1021/jacs.7b02979

[46] Jiang Y, Tan P, Qi SC, et al. Metal–organic frameworks with target-specific active sites switched by photoresponsive motifs: efficient adsorbents for tailorable CO_2_ capture. Angew Chem Int Ed. 2019;58(20):6600–6604. DOI:10.1002/anie.201900141 30714664

[47] Healey K, Liang W, Southon PD, et al. Photoresponsive spiropyran-functionalised MOF-808: postsynthetic incorporation and light dependent gas adsorption properties. J Mater Chem A. 2016;4(28):10816–10819. DOI:10.1039/C6TA04160D

[48] Choi HJ, Dinca M, Long JR. Broadly hysteretic H_2_ adsorption in the microporous metal−organic framework Co(1,4-benzenedipyrazolate). J Am Chem Soc. 2008;130(25):7848–7850. 18512921 10.1021/ja8024092

[49] Zeleňák V, Vargová Z, Almáši M, et al. Layer-pillared zinc(II) metal–organic framework built from 4,4′-azo(bis)pyridine and 1,4-BDC. Microporous Mesoporous Mater. 2010;129(3):354–359. DOI:10.1016/j.micromeso.2009.11.002

[50] Maji TK, Uemura K, Chang H-C, et al. Expanding and shrinking porous modulation based on pillared-layer coordination polymers showing selective guest adsorption. Angew Chem. 2004;116(25):3331–3334. DOI:10.1002/ange.200453923 15213951

[51] Noro S, Kitagawa S, Nakamura T, et al. Synthesis and crystallographic characterization of low-dimensional and porous coordination compounds capable of supramolecular aromatic interaction using the 4,4‘-Azobis(pyridine) ligand. Inorg Chem. 2005;44(11):3960–3971. DOI:10.1021/ic048371u 15907124

[52] Kondo M, Shimamura M, Noro S, et al. Microporous materials constructed from the interpenetrated coordination networks. Structures and methane adsorption properties. Chem Mater. 2000;12(5):1288–1299. DOI:10.1021/cm990612m

[53] Noro S, Kondo M, Ishii T, et al. Syntheses and crystal structures of iron co-ordination polymers with 4,4′-bipyridine (4,4′-bpy) and 4,4′-azopyridine (azpy). Two-dimensional networks supported by hydrogen bonding, {[Fe(azpy)(NCS)_2_(MeOH)_2_]·azpy}_n_ and {[Fe(4,4′-bpy)(NCS)_2_(H_2_O)_2_]·4,4′-bpy}_n_. J Chem Soc Dalt Trans. 1999;2010(10):1569–1574. DOI:10.1039/a809523j

[54] Kondo M, Shimamura M, Noro S, et al. Syntheses and structures of Zn coordination polymers with 4,4′-bipyridine and 4,4′-azopyridine. Effect of counter anions on the network system. Chem Lett. 1999;28(4):285–286. DOI:10.1246/cl.1999.285

[55] Kobatake S, Kitagawa D. Photomechanical behavior of photochromic diarylethene crystals. In: Koshima H, editor. Mech responsive mater soft robot. 1st ed. Weinheim, Germany: Wiley; 2020. pp. 1–28.

[56] Lyndon R, Konstas K, Ladewig BP, et al. Dynamic photo-switching in metal-organic frameworks as a route to low-energy carbon dioxide capture and release. Angew Chem Int Ed. 2013;52(13):3695–3698. DOI:10.1002/anie.201206359 23401101

[57] Li H, Martinez MR, Perry Z, et al. A robust metal–organic framework for dynamic light-induced swing adsorption of carbon dioxide. Chem - A Eur J. 2016;22(32):11176–11179. DOI:10.1002/chem.201602671 27273621

[58] Prasetya N, Teck AA, Ladewig BP. Matrimid-JUC-62 and matrimid-PCN-250 mixed matrix membranes displaying light-responsive gas separation and beneficial ageing characteristics for CO_2_/N_2_ separation. Sci Rep. 2018;8(1):2944. 29440732 10.1038/s41598-018-21263-7 PMC5811445

[59] Jiang Y, Tan P, Qi SC, et al. Breathing metal–organic polyhedra controlled by light for carbon dioxide capture and liberation. CCS Chem. 2020;3(6):1659–1668. DOI:10.31635/ccschem.020.202000314

[60] Drake HF, Xiao Z, Day GS, et al. Influence of metal identity on light-induced switchable adsorption in azobenzene-based metal–organic frameworks. ACS Appl Mater Interfaces. 2022;14(9):11192–11199. DOI:10.1021/acsami.1c18266 35192321

[61] Prasetya N, Ladewig BP. Dynamic photo-switching in light-responsive JUC-62 for CO_2_ capture. Sci Rep. 2017;7(1):13355. 29042605 10.1038/s41598-017-13536-4 PMC5645403

[62] Zheng Y, Sato H, Wu P, et al. Flexible interlocked porous frameworks allow quantitative photoisomerization in a crystalline solid. Nat Commun. 2017;8(1):100. DOI:10.1038/s41467-017-00122-5 28740107 PMC5524650

[63] Hazra A, Bonakala S, Adalikwu SA, et al. Fluorocarbon-functionalized superhydrophobic metal–organic framework: enhanced CO_2_ uptake via photoinduced postsynthetic modification. Inorg Chem. 2021;60(6):3823–3833. DOI:10.1021/acs.inorgchem.0c03575 33655749

[64] Xue M, Zhu G, Li Y, et al. Structure, hydrogen storage, and luminescence properties of three 3D metal−organic frameworks with NbO and PtS topologies. Cryst Growth Des. 2008;8(7):2478–2483. DOI:10.1021/cg8001114

[65] Stoeck U, Krause S, Bon V, et al. A highly porous metal–organic framework, constructed from a cuboctahedral super-molecular building block, with exceptionally high methane uptake. Chem Commun. 2012;48(88):10841–10843. DOI:10.1039/c2cc34840c 23033252

[66] Krause S, Evans JD, Bon V, et al. Cooperative light-induced breathing of soft porous crystals via azobenzene buckling. Nat Commun. 2022;13(1):1951. DOI:10.1038/s41467-022-29149-z 35414051 PMC9005654

[67] Krause S, Evans JD, Bon V, et al. Engineering micromechanics of soft porous crystals for negative gas adsorption. Chem Sci. 2020;11(35):9468–9479. DOI:10.1039/D0SC03727C 34094213 PMC8162094

[68] Zhai C, Lin S, Wang M, et al. Conformational freedom-enhanced optomechanical energy conversion efficiency in bulk azo-polyimides. Adv Funct Mater. 2021;31(45):2104414. DOI:10.1002/adfm.202104414

[69] Hermann D, Emerich H, Lepski R, et al. Metal–organic frameworks as hosts for photochromic guest molecules. Inorg Chem. 2013;52(5):2744–2749. DOI:10.1021/ic302856b 23409796

[70] Hermann D, Schwartz HA, Werker M, et al. Metal-organic frameworks as hosts for fluorinated azobenzenes: a path towards quantitative photoswitching with visible light. Chem Eur J. 2019;25(14):3606–3616. DOI:10.1002/chem.201805391 30633421

[71] Garg S, Schwartz H, Kozlowska M, et al. Conductance photoswitching of metal–organic frameworks with embedded spiropyran. Angew Chem Int Ed. 2019;58(4):1193–1197. DOI:10.1002/anie.201811458 30421842

[72] Tu M, Reinsch H, Rodríguez-Hermida S, et al. Reversible optical writing and data storage in an anthracene‐loaded metal‐organic framework. Angew Chem. 2018;2445–2449. DOI:10.1002/ange.201813996. 30548136

[73] Myers AL, Prausnitz JM. Thermodynamics of mixed-gas adsorption. AIChE J. 1965;11(1):121–127.

[74] Kim H, Yang S, Rao SR, et al. Water harvesting from air with metal-organic frameworks powered by natural sunlight. Science. 2017;356(6336):430–434. DOI:10.1126/science.aam8743 28408720

[75] Al-Rowaili FN, Zahid U, Onaizi S, et al. A review for metal-organic frameworks (MOFs) utilization in capture and conversion of carbon dioxide into valuable products. J CO2 Util. 2021;53:101715.

[76] Leodopoulos C, Doulia D, Gimouhopoulos K. Adsorption of cationic dyes onto bentonite. Sep Purif Rev. 2014;44(1):74–107.

[77] Phuekphong AF, Imwiset KJ, Ogawa M. Designing nanoarchitecture for environmental remediation based on the clay minerals as building block. J Hazard Mater. 2020;399:122888. 32937697 10.1016/j.jhazmat.2020.122888

[78] Okada T, Seki Y, Ogawa M. Designed nanostructures of clay for controlled adsorption of organic compounds. J Nanosci Nanotechnol. 2014;14(3):2121–2134. 24745206 10.1166/jnn.2014.8597

[79] Sanchez C, Julián B, Belleville P, et al. Applications of hybrid organic–inorganic nanocomposites. J Mater Chem. 2005;15(35–36):3559–3592. DOI:10.1039/b509097k

[80] Martínez-Martínez V, López Arbeloa F, editors. Dyes and photoactive molecules in microporous systems. Berlin, Heidelberg: Springer International Publishing; 2020. (Structure and Bonding; vol. 183).

[81] Teepakakorn AP, Yamaguchi T, Ogawa M. The improved stability of molecular guests by the confinement into nanospaces. Chem Lett. 2019;48(5):398–409.

[82] Choi G, Kim TH, Oh JM, et al. Emerging nanomaterials with advanced drug delivery functions; focused on methotrexate delivery. Coord Chem Rev. 2018;359:32–51.

[83] Ogawa M, Kuroda K. Photofunctions of intercalation compounds. Chem Rev. 1995;95(2):399–438.

[84] Yamaguchi T, Oh J-M, Ogawa M. Photofunctions of dye-clay hybrids: recent developments. In: Martínez-Martínez V López Arbeloa F, editors. Structure and Bonding. Berlin, Heidelberg: Springer; 2020. p. 251–320.

[85] Sasai R, Ogiso H, Shindachi I, et al. Photochromism in oriented thin films prepared by the hybridization of diarylethenes in clay interlayers. Tetrahedron. 2000;56(36):6979–6984. DOI:10.1016/S0040-4020(00)00519-6

[86] Shindachi I, Hanaki H, Sasai R, et al. The effect of layered sodium–magadiite on the photochromic reversibility of diarylethene immobilized on its surfaces. Chem Lett. 2004;33(9):1116–1117. DOI:10.1246/cl.2004.1116

[87] Sasai R, Itoh H, Shindachi I, et al. Photochromism of clay−diarylethene hybrid materials in optically transparent gelatin films. Chem Mater. 2001;13(6):2012–2016. DOI:10.1021/cm000822v

[88] Nakamura T, Takagi K, Itoh M, et al. Photodimerization of cinnamic acids controlled by molecular assemblies of surfactant amine *N*-oxides. J Chem Soc Perkin Trans 2. 1997;2(12):2751–2755. DOI:10.1039/a702413d

[89] Shichi T, Takagi K, Sawaki Y. Stereoselectivity control of [2 + 2] photocycloaddition by changing site distances of hydrotalcite interlayers. Chem Commun. 1996;17(17):2027–2028.

[90] Shichi T, Yamashita S, Takagi K. Photopolymerization of *4*-vinylbenzoate and *m*- and *p*-phenylenediacrylates in hydrotalcite interlayers. Supramol Sci. 1998;5(3–4):303–308.

[91] Takagi K, Shichi T, Usami H, et al. Controlled photocycloaddition of unsaturated carboxylates intercalated in hydrotalcite clay interlayers. J Am Chem Soc. 1993;115(10):4339–4344. DOI:10.1021/ja00063a060

[92] Takagi K, Usami H, Fukaya H, et al. Spatially controlled photocycloaddition of a clay-intercalated stilbazolium cation. J Chem Soc Chem Commun. 1989;(16):1174–1175. DOI:10.1039/c39890001174.

[93] Sasai R, Shin’ya N, Shichi T, et al. Molecular alignment and photodimerization of 4′-Chloro-4-stilbenecarboxylic acid in hydrotalcite clays: bilayer formation in the interlayers. Langmuir. 2002;15(2):413–418. DOI:10.1021/la980699a

[94] Kashima I, Okubo M, Qno Y, et al. Ferromagnetism and its photo-induced effect in 2D iron mixed-valence complex coupled with photochromic spiropyran. Synth Met. 2005;155(3):703–706. DOI:10.1016/j.synthmet.2005.09.033

[95] Enomoto M, Kojima N. Charge transfer phase transition and ferromagnetism in a novel iron mixed-valence complex (*n*-C_3_H_7_)_4_N[Fe^II^Fe^III^(tto)_3_] (tto=C_2_OS_3_). Synth Met. 2005;152(1–3):457–460.

[96] Kida N, Hikita M, Kashima I, et al. Mössbauer spectroscopic study of photo-sensitive organic–inorganic hybrid system, (SP)[Fe(II)Fe(III)(dto)_3_](dto = C_2_O_2_S_2_, SP = spiropyran). Polyhedron. 2009;28(9–10):1694–1697. DOI:10.1016/j.poly.2008.10.060

[97] Kida N, Hikita M, Kashima I, et al. Control of charge transfer phase transition and ferromagnetism by photoisomerization of spiropyran for an organic−inorganic hybrid system, (SP)[Fe^II^Fe^III^(dto)_3_] (SP= spiropyran, dto= C_2_O_2_S_2_. J Am Chem Soc. 2009;131(1):212–220. DOI:10.1021/ja806879a 19072154

[98] Tanaka N, Okazawa A, Sugahara A, et al. Development of a photoresponsive organic–inorganic hybrid magnet: layered cobalt hydroxides intercalated with spiropyran anions. Bull Chem Soc Jpn. 2015;88(8):1150–1155. DOI:10.1246/bcsj.20150129

[99] Abellán G, Coronado E, Martí-Gastaldo C, et al. Photo-switching in a hybrid material made of magnetic layered double hydroxides intercalated with azobenzene molecules. Adv Mater. 2014;26(24):4156–4162. DOI:10.1002/adma.201400713 24706546

[100] Abellán G, Jordá JL, Atienzar P, et al. Stimuli-responsive hybrid materials: breathing in magnetic layered double hydroxides induced by a thermoresponsive molecule. Chem Sci. 2015;6(3):1949–1958. DOI:10.1039/C4SC03460K 28706645 PMC5495995

[101] Okubo M, Enomoto M, Kojima N. Study on photomagnetism of 2-D magnetic compounds coupled with photochromic diarylethene cations. Synth Met. 2005;152(1–3):461–464.

[102] Ogawa M, Fujii K, Kuroda K, et al. Preparation of montmorillonite-*p*-aminoazobenzene intercalation compounds and their photochemical behavior. Mater Res Soc Symp Proc. 1991;233:89–94.

[103] Okada T, Nozaki N, Seo J, et al. Photoinduced structural changes of cationic azo dyes confined in a two dimensional nanospace by two different mechanisms. RSC Adv. 2017;7(13):8077–8081. DOI:10.1039/C6RA27749G

[104] Okada T, Sakai H, Ogawa M. The effect of the molecular structure of a cationic azo dye on the photoinduced intercalation of phenol in a montmorillonite. Appl Clay Sci. 2008;40(1–4):187–192.

[105] Ogawa M. Photoisomerization of azobenzene in the interlayer space of magadiite. J Mater Chem. 2002;12(11):3304–3307.

[106] Ogawa M, Ishikawa A. Controlled microstructures of amphiphilic cationic azobenzene-montmorillonite intercalation compounds. J Mater Chem. 1998;8(2):463–467.

[107] Ogawa M, Ishii T, Miyamoto N, et al. Intercalation of a cationic azobenzene into montmorillonite. Appl Clay Sci. 2003;22(4):179–185. DOI:10.1016/S0169-1317(02)00157-6

[108] Kim CS, Yates DM, Heaney PJ. The layered sodium silicate magadiite: an analog to smectite for benzene sorption from water. Clays Clay Miner. 1997;45(6):881–885.

[109] Ogawa M, Ishii T, Miyamoto N, et al. Photocontrol of the basal spacing of azobenzene–magadiite intercalation compound. Adv Mater. 2001;13(14):1107–1109. DOI:10.1002/1521-4095(200107)13:14<1107:AID-ADMA1107>3.0.CO;2-O

[110] Heinz H, Vaia RA, Koerner H, et al. Photoisomerization of azobenzene grafted to layered silicates: simulation and experimental challenges. Chem Mater. 2008;20(20):6444–6456. DOI:10.1021/cm801287d

[111] Fujita T, Iyi N, Klapyta Z. Optimum conditions for photoresponse of azobenzene-organophilic tetrasilicic mica complexes. Mater Res Bull. 2001;36(3–4):557–571.

[112] Fujita T, Iyi N, Klapyta Z, et al. Photomechanical response of azobenzene/organophilic mica complexes. Mater Res Bull. 2003;38(15):2009–2017. DOI:10.1016/j.materresbull.2003.09.012

[113] Ogawa M, Iwata D. Arrangements of interlayer quaternary ammonium ions in a layered silicate, octosilicate. Cryst Growth Des. 2010;10(5):2068–2072.

[114] Lagaly G, Fernandez Gonzalez M, Weiss A. Problems in layer-charge determination of montmorillonites. Clay Miner. 1976;11(3):173–187.

[115] Koteja A, Szczerba M, Matusik J. Smectites intercalated with azobenzene and aminoazobenzene: structure changes at nanoscale induced by UV light. J Phys Chem Solids. 2017;111:294–303.

[116] Abbaszad Rafi A, Hamidi N, Bashir-Hashemi A, et al. Photo-switchable nanomechanical systems comprising a nanocontainer (montmorillonite) and light-driven molecular jack (azobenzene-imidazolium ionic liquids) as drug delivery systems; synthesis, characterization, and in vitro release studies. ACS Biomater Sci Eng. 2018;4(1):184–192. DOI:10.1021/acsbiomaterials.7b00621 33418688

[117] Okada T, Watanabea Y, Ogawa M. Photocontrol of the adsorption behavior of phenol for an azobenzene-montmorillonite intercalation compound. Chem Commun. 2004;1(3):320–321. 10.1039/b312962d 14740057

[118] Okada T, Watanabe Y, Ogawa M. Photoregulation of the intercalation behavior of phenol for azobenzene–clay intercalation compounds. J Mater Chem. 2005;15(9):987.

[119] Nabetani Y, Takamura H, Hayasaka Y, et al. An artificial muscle model unit based on inorganic nanosheet sliding by photochemical reaction. Nanoscale. 2013;5(8):3182–3193. DOI:10.1039/c3nr34308a 23471173

[120] Nabetani Y, Takamura H, Hayasaka Y, et al. A photoactivated artificial muscle model unit: reversible, photoinduced sliding of nanosheets. J Am Chem Soc. 2011;133(43):17130–17133. DOI:10.1021/ja207278t 21978075

[121] Nabetani Y, Takamura H, Uchikoshi A, et al. Photo-induced morphological winding and unwinding motion of nanoscrolls composed of niobate nanosheets with a polyfluoroalkyl azobenzene derivative. Nanoscale. 2016;8(24):12289–12293. DOI:10.1039/C6NR02177H 27273772

[122] Tong Z, Takagi S, Shimada T, et al. Photoresponsive multilayer spiral nanotubes: intercalation of polyfluorinated cationic azobenzene surfactant into potassium niobate. J Am Chem Soc. 2006;128(3):684–685. DOI:10.1021/ja0564564 16417334

[123] Guo S, Matsukawa K, Miyata T, et al. Photoinduced bending of self-assembled azobenzene–siloxane hybrid. J Am Chem Soc. 2015;137(49):15434–15440. DOI:10.1021/jacs.5b06172 26575345

[124] Helmy S, Leibfarth FA, Oh S, et al. Photoswitching using visible light : a new class of organic photochromic molecules. J Am Chem Soc. 2014;136(23):8169–8172. DOI:10.1021/ja503016b 24848124

[125] Gomes RFA, Coelho JAS, Afonso CAM. Synthesis and applications of Stenhouse salts and derivatives. Chem - A Eur J. 2018;24(37):9170–9186. 10.1002/chem.201705851 29393530

[126] Lerch MM, Szymański W, Feringa BL. The (photo)chemistry of stenhouse photoswitches: guiding principles and system design. Chem Soc Rev. 2018;47(6):1910–1937. 29468232 10.1039/c7cs00772h

[127] García-López V, Chen F, Nilewski LG, et al. Molecular machines open cell membranes. Nature. 2017;548(7669):567–572. DOI:10.1038/nature23657 28858304

[128] Shinkai S, Minami T, Kusano Y, et al. Photoresponsive crown ethers 5. Light-driven ion transport by crown ethers with a photoresponsive anionic cap. J Am Chem Soc. 1982;104(7):1967–1972. DOI:10.1021/ja00371a028

[129] Shinkai S, Shigematsu K, Sato M, et al. Photoresponsive crown ethers. Part 6. Ion transport mediated by photoinduced *cis—trans* interconversion of azobis(benzocrown ethers). J Chem Soc Perkin Trans 1. 1982;2735–2739. DOI:10.1039/P19820002735.

[130] Shinkai S, Ishihara M, Ueda K, et al. Photoresponsive crown ethers Part 14. Photoregulated crown–metal complexation by competitive intramolecular tail(ammonium)-biting. J Chem Soc Perkin Trans 2. 1985;7(4):511–518. DOI:10.1039/P29850000511

[131] Sakamoto H, Takagaki H, Nakamura M, et al. Photoresponsive liquid membrane transport of alkali metal ions using crowned spirobenzopyrans. Anal Chem. 2005;77(7):1999–2006. DOI:10.1021/ac048642i 15801730

[132] Khairutdinov RF, Hurst JK. Light-driven transmembrane ion transport by spiropyran−crown ether supramolecular assemblies. Langmuir. 2004;20(5):1781–1785.

[133] Xie X, Crespo GA, Mistlberger G, et al. Photocurrent generation based on a light-driven proton pump in an artificial liquid membrane. Nat Chem. 2014;6(3):202–207. DOI:10.1038/nchem.1858 24557134

[134] Steinberg-Yfrach G, Liddell PA, Hung S-C, et al. Conversion of light energy to proton potential in liposomes by artificial photosynthetic reaction centres. Nature. 1997;385:239–241.

[135] Steinberg-Yfrach G, Rigaud JL, Durantini EN, et al. Light-driven production of ATP catalysed by F_0_F_1_-ATP synthase in an artificial photosynthetic membrane. Nature. 1998;392(6675):479–482. DOI:10.1038/33116 9548252

[136] de Thieulloy L, Barois C, Mongin C, et al. Is it possible to”simply” predict the photoejection of a cation? Example of azacrown-substituted [(bpy)Re(CO)_3_L]^+^ complexes. J Photochem Photobiol A Chem. 2022;426:113714.

[137] Bennett IM, Vanegas Farfano HM, Bogani F, et al. Active transport of Ca^2+^ by an artificial photosynthetic membrane. Nature. 2002;420:398–401. 12459780 10.1038/nature01209

[138] Lewis JD, Perutz RN, Moore JN. Light-controlled ion switching: direct observation of the complete nanosecond release and microsecond recapture cycle of an azacrown-substituted [(bpy)Re(CO)_3_L]^+^ complex. J Phys Chem A. 2004;108(42):9037–9047.

[139] Salunke SB, Malla JA, Talukdar P. Phototriggered release of a transmembrane chloride carrier from an *o*-nitrobenzyl-linked procarrier. Angew Chem Int Ed. 2019;58(16):5354–5358. 10.1002/anie.201900869 30758120

[140] Shinkai S, Honda Y, Ueda K, et al. Photoresponsive crown ethers. 12. Photocontrol of metal ion complexation with thiacrown ethers. Bull Chem Soc Jpn. 1984;57(8):2144–2149. DOI:10.1246/bcsj.57.2144

[141] Shinkai S, Shigematsu K, Honda Y, et al. Photoresponsive crown ethers 13. Synthesis of photoresponsive NS_2_O crown ethers and application of the Cu(I) complexes to O_2_-Binding. Bull Chem Soc Jpn. 1984;57:2879–2884.

[142] Shinkai S, Miyazaki K, Manabe O. Photoresponsive crown ethers. Part 18. Photochemically “switched-on” crown ethers containing an intra-annular azo substituent and their application to membrane transport. J Chem Soc Perkin Trans 1. 1987;449–456. DOI:10.1039/P19870000449

[143] Shinkai S, Ogawa T, Nakaji T, et al. Photocontrolled extraction ability of azobenzene-bridged azacrown ether. Tetrahedron Lett. 1979;20(47):4569–4572. DOI:10.1016/S0040-4039(01)86651-X

[144] Shinkai S, Nakaji T, Nishida Y, et al. Photoresponsive crown ethers. 1. Cis-trans isomerism of azobenzene as a tool to enforce conformational changes of crown ethers and polymers. J Am Chem Soc. 1980;102(18):5860–5865. DOI:10.1021/ja00538a026

[145] Shinkai S, Shigematsu K, Kusano Y, et al. Photoresponsive crown ethers. Part 3. Photocontrol of ion extraction and ion transport by several photofunctional bis(crown ethers). J Chem Soc Perkin Trans 1. 1981;3279–3283. DOI:10.1039/p19810003279.

[146] Shinkai S, Ogawa T, Kusano Y, et al. Photoresponsive crown ethers. 4. influence of alkali metal cations on photoisomerization and thermal isomerization of azobis(benzocrown ether)s. J Am Chem Soc. 1982;104(7):1960–1967. DOI:10.1021/ja00371a027

[147] Shinkai S, Minami T, Kusano Y, et al. Photoresponsive crown ethers. 8. Azobenzenophane-type “switched-on” crown ethers which exhibit an all-or-nothing change in ion-binding ability. J Am Chem Soc. 1983;105(7):1851–1856. DOI:10.1021/ja00345a029

[148] Shinkai S, Honda Y, Minami T, et al. Photoresponsive crown ethers. 9. Cylindrical and phane crown ethers with azobenzene segments as a light-switch functional group. Bull Chem Soc Jpn. 1983;56(6):1700–1704. DOI:10.1246/bcsj.56.1700

[149] Shinkai S, Honda Y, Ueda K, et al. Photoresponsive crown ethers. Part 11. Azobenzene‐pillared cylindrical macrocycle as a photoresponsive receptor. ISR J Chem. 1984;24(4):302–306. DOI:10.1002/ijch.198400052

[150] Choi YR, Kim GC, Jeon HG, et al. Azobenzene-based chloride transporters with light-controllable activities. Chem Commun. 2014;50(97):15305–15308. DOI:10.1039/C4CC07560A 25350406

[151] Kerckhoffs A, Langton MJ. Reversible photo-control over transmembrane anion transport using visible-light responsive supramolecular carriers. Chem Sci. 2020;11(24):6325–6331. 32953027 10.1039/d0sc02745f PMC7472928

[152] Ahmad M, Metya S, Das A, et al. A sandwich azobenzene–diamide dimer for photoregulated chloride transport. Chem Eur J. 2020;26(40):8703–8708. DOI:10.1002/chem.202000400 32129531

[153] Li H, Zhang Y, Jia Z, et al. Theoretical design on molecular tweezers of sodium cyanide by zinc porphyrin-azo-crown ether triads receptor. J Phys Org Chem. 2019;32(8):1–10. DOI:10.1002/poc.3963

[154] Shinkai S, Kinda H, Ishihara M, et al. Photoresponsive crown ethers. 10. Metal complexation by light-switched crown ethers immobilized in polymer matrices. J Polym Sci Part A. 1983;21(12):3525–3539. DOI:10.1002/pol.1983.170211214

[155] Sakamoto H, Yokohata T, Yamamura T, et al. Liquid−liquid extraction of alkali metal ions with photochromic crowned spirobenzopyrans. Anal Chem. 2002;74(11):2522–2528. DOI:10.1021/ac020003p 12069232

[156] Li E, Kang J, Ye P, et al. A prospective material for the highly selective extraction of lithium ions based on a photochromic crowned spirobenzopyran. J Mater Chem B. 2019;7(6):903–907. DOI:10.1039/C8TB02906G 32255095

[157] Kimura K, Nakahara Y. Analytical and separation chemistry by taking advantage of organic photochromism combined with macrocyclic chemistry. Anal Sci. 2009;25(1):9–20. 19139568 10.2116/analsci.25.9

[158] Nakamura M, Sakamoto H, Kimura K. Photocontrollable cation extraction with crowned oligo(spirobenzopyran)s. Anal Sci. 2005;21(4):403–408. 15844334 10.2116/analsci.21.403

[159] Kimura K, Sakamoto H, Nakamura M. Molecular design and applications of photochromic crown compounds —how can we manipulate metal ions photochemically?. Bull Chem Soc Jpn. 2003;76(2):225–245.

[160] Kandori H, Inoue K, Tsunoda SP. Light-driven sodium-pumping rhodopsin: a new concept of active transport. Chem Rev. 2018;118(21):10646–10658. 29513519 10.1021/acs.chemrev.7b00548

[161] Engelhard C, Chizhov I, Siebert F, et al. Microbial halorhodopsins: light-driven chloride pumps. Chem Rev. 2018;118(21):10629–10645. DOI:10.1021/acs.chemrev.7b00715 29882660

[162] Xie G, Li P, Zhao Z, et al. Bacteriorhodopsin-inspired light-driven artificial molecule motors for transmembrane mass transportation. Angew Chem Int Ed. 2018;57(51):16708–16712. DOI:10.1002/anie.201809627 30358031

[163] Xiao K, Giusto P, Chen F, et al. Light-driven directional ion transport for enhanced osmotic energy harvesting. Natl Sci Rev. 2021;8(8):nwaa231. DOI:10.1093/nsr/nwaa231 34691706 PMC8363323

[164] Koçer A, Walko M, Meijberg W, et al. A light-actuated nanovalve derived from a channel protein. Science. 2005;309(5735):755–758. DOI:10.1126/science.1114760 16051792

[165] Bonardi F, London G, Nouwen N, et al. Light-induced control of protein translocation by the SecYEG complex. Angew Chem Int Ed. 2010;49(40):7234–7238. DOI:10.1002/anie.201002243 20803592

[166] Muramatsu S, Kinbara K, Taguchi H, et al. Semibiological molecular machine with an implemented “AND” logic gate for regulation of protein folding. J Am Chem Soc. 2006;128(11):3764–3769. DOI:10.1021/ja057604t 16536551

[167] Sendai T, Biswas S, Aida T. Photoreconfigurable supramolecular nanotube. J Am Chem Soc. 2013;135(31):11509–11512. 23875534 10.1021/ja4060146

[168] Yang X, Ma G, Zheng S, et al. Optical control of CRAC channels using photoswitchable azopyrazoles. J Am Chem Soc. 2020;142(20):9460–9470. DOI:10.1021/jacs.0c02949 32330031

[169] Sukharev S, Anishkin A. Mechanosensitive channels: what can we learn from “simple” model systems? Trends Neurosci. 2004;27(6):345–351. 15165739 10.1016/j.tins.2004.04.006

[170] du Plessis DJF, Berrelkamp G, Nouwen N, et al. The lateral gate of SecYEG opens during protein translocation. J Biol Chem. 2009;284(23):15805–15814. DOI:10.1074/jbc.M901855200 19366685 PMC2708877

[171] Bukau B, Horwich AL. The Hsp70 and Hsp60 chaperone machines. Cell. 1998;92:351–366. 9476895 10.1016/s0092-8674(00)80928-9

[172] Berridge MJ, Bootman MD, Roderick HL. Calcium signalling: dynamics, homeostasis and remodelling. Nat Rev Mol Cell Biol. 2003;4(7):517–529. 12838335 10.1038/nrm1155

[173] Derler I, Schindl R, Fritsch R, et al. The action of selective CRAC channel blockers is affected by the Orai pore geometry. Cell Calcium. 2013;53(2):139–151. DOI:10.1016/j.ceca.2012.11.005 23218667 PMC3580291

[174] Fyles TM, James TD, Kaye KC. Activities and modes of action of artificial ion-channel mimics. J Am Chem Soc. 1993;115(26):12315–12321.

[175] Voyer N, Robitaille M. A novel functional artificial ion channel. J Am Chem Soc. 1995;117(24):6599–6600.

[176] Hu Y, Roberts JM, Kilgore HR, et al. Triple, mutually orthogonal bioorthogonal pairs through the design of electronically activated sulfamate-containing cycloalkynes. J Am Chem Soc. 2020;142(44):18859–18865. DOI:10.1021/jacs.0c06725 33085477 PMC7891878

[177] Liu T, Bao C, Wang H, et al. Self-assembly of crown ether-based amphiphiles for constructing synthetic ion channels: the relationship between structure and transport activity. New J Chem. 2014;38(8):3507–3513. DOI:10.1039/C4NJ00297K

[178] Bhosale S, Sisson AL, Talukdar P, et al. Photoproduction of proton gradients with π-stacked fluorophore scaffolds in lipid bilayers. Science. 2006;313(5783):84–86. DOI:10.1126/science.1126524 16825567

[179] Bao C, Ma M, Meng F, et al. Efficient synthetic supramolecular channels and their light-deactivated ion transport in bilayer lipid membranes. New J Chem. 2015;39(8):6297–6302. DOI:10.1039/C5NJ00937E

[180] Zhou Y, Chen Y, Zhu P, et al. Reversible photo-gated transmembrane channel assembled from an acylhydrazone-containing crown ether triad. Chem Commun. 2017;53(26):3681–3684. DOI:10.1039/C7CC01123G 28294246

[181] Liu T, Bao C, Wang H, et al. Light-controlled ion channels formed by amphiphilic small molecules regulate ion conduction *via cis–trans* photoisomerization. Chem Commun. 2013;49(87):10311–10313. DOI:10.1039/c3cc45618h 24064555

[182] Wang WZ, Huang LB, Zheng SP, et al. Light-driven molecular motors boost the selective transport of alkali metal ions through phospholipid bilayers. J Am Chem Soc. 2021;143(38):15653–15660. DOI:10.1021/jacs.1c05750 34520204

[183] Matile S, Sakai N. The characterization of synthetic ion channels and pores. In: Schalley C, editor. Analytical Methods in Supramolecular Chemistry. Weinheim, Germany: Wiley-VCH; 2007. pp. 391–418.

[184] Vlassiouk I, Park C-D, Vail SA, et al. Control of nanopore wetting by a photochromic spiropyran: a light-controlled valve and electrical switch. Nano Lett. 2006;6(5):1013–1017. DOI:10.1021/nl060313d 16683842 PMC2529173

[185] Wang G, Bohaty AK, Zharov I, et al. Photon gated transport at the glass nanopore electrode. J Am Chem Soc. 2006;128(41):13553–13558. DOI:10.1021/ja064274j 17031969

[186] Bohaty AK, Newton MR, Zharov I. Light-controlled ion transport through spiropyran-modified nanoporous silica colloidal films. J Porous Mater. 2010;17(4):465–473.

[187] Zhang M, Hou X, Wang J, et al. Light and pH cooperative nanofluidic diode using a spiropyran-functionalized single nanochannel. Adv Mater. 2012;24(18):2424–2428. DOI:10.1002/adma.201104536 22488964

[188] Takashima Y, Harada A. Functioning via host–guest interactions. J Incl Phenom Macrocyclic Chem. 2017;87(3–4):313–330.

[189] Harada A, Kobayashi R, Takashima Y, et al. Macroscopic self-assembly through molecular recognition. Nat Chem. 2011;3(1):34–37. DOI:10.1038/nchem.893 21160514

[190] Liu P, Zhou T, Teng Y, et al. Light-induced heat driving active ion transport based on 2D MXene nanofluids for enhancing osmotic energy conversion. CCS Chem. 2021;3(4):1325–1335. DOI:10.31635/ccschem.020.202000296

[191] Lin F, Lonergan MC. Semiconducting bipolar membranes: photochemical salt pumps. ACS Appl Energy Mater. 2020;3(5):4103–4107.

[192] Barrio J, Sánchez-Somolinos C. Light to shape the future : from photolithography to 4D printing. Adv Opt Mater. 2019;7(16):1900598.

[193] Phillips HM, Callahan DL, Sauerbrey R, et al. Sub-100 nm lines produced by direct laser ablation in polyimide. Appl Phys Lett. 1991;58(24):2761–2763. DOI:10.1063/1.104778

[194] Natansohn A, Rochon P. Photoinduced motions in azo-containing polymers. Chem Rev. 2002;102(11):4139–4175. 12428986 10.1021/cr970155y

[195] Viswanathan NK, Kim DY, Bian S, et al. Surface relief structures on azo polymer films. J Mater Chem. 1999;9(9):1941–1955. DOI:10.1039/a902424g

[196] Oscurato SL, Salvatore M, Maddalena P, et al. From nanoscopic to macroscopic photo-driven motion in azobenzene-containing materials. Nanophotonics. 2018;7(8):1387–1422. DOI:10.1515/nanoph-2018-0040

[197] Lee S, Kang HS, Park JK. Directional photofluidization lithography: micro/nanostructural evolution by photofluidic motions of azobenzene materials. Adv Mater. 2012;24(16):2069–2103. 22454301 10.1002/adma.201104826

[198] Kim CB, Janes DW, Zhou SX, et al. Bidirectional control of flow in thin polymer films by photochemically manipulating surface tension. Chem Mater. 2015;27(13):4538–4545. DOI:10.1021/acs.chemmater.5b01744

[199] Jones AR, Kim CB, Zhou SX, et al. Generating large thermally stable marangoni-driven topography in polymer films by stabilizing the surface energy gradient. Macromolecules. 2017;50(11):4588–4596. DOI:10.1021/acs.macromol.7b00055

[200] Dai M, Picot OT, Hughes-Brittain NF, et al. Formation of relief structures on fibres by photo-embossing. J Mater Chem. 2011;21(39):15527–15531. DOI:10.1039/c1jm12365c

[201] Liedtke A, Lei C, O’Neill M, et al. One-step photoembossing for submicrometer surface relief structures in liquid crystal semiconductors. ACS Nano. 2010;4(6):3248–3253. DOI:10.1021/nn100012g 20455584

[202] Ubukata T, Nakayama M, Sonoda T, et al. Highly sensitive formation of stable surface relief structures in bisanthracene films with spatially patterned photopolymerization. ACS Appl Mater Interfaces. 2016;8(34):21974–21978. DOI:10.1021/acsami.6b07943 27513175

[203] Kawatsuki N, Tashima A, Manabe S, et al. Holographic recording in a photo-cross-linkable liquid crystalline copolymer using a 325-nm laser with various polarizations. React Funct Polym. 2010;70(12):980–985. DOI:10.1016/j.reactfunctpolym.2010.10.006

[204] Lavielle L, Croutxé-Barghorn C, Schuller E, et al. Photopolymerization of acrylate films under an imposed spatial irradiation: thermodynamics of relief self-development. J Colloid Interface Sci. 1997;192(1):149–155. DOI:10.1006/jcis.1997.4972 9268552

[205] Kawatsuki N, Hasegawa T, Ono H, et al. Formation of polarization gratings and surface relief gratings in photocrosslinkable polymer liquid crystals by polarization holography. Adv Mater. 2003;15(12):991–994. DOI:10.1002/adma.200304988

[206] Veltri A, Caputo R, Umeton C, et al. Model for the photoinduced formation of diffraction gratings in liquid-crystalline composite materials. Appl Phys Lett. 2004;84(18):3492–3494. DOI:10.1063/1.1738182

[207] Goldenberg L, Sakhno O, Stumpe J. Application of Norland adhesive for holographic recording. Opt Mater. 2005;27(8):1379–1385.

[208] Aoki K, Ichimura K. Self-developable surface relief photoimaging generated by anionic UV-curing of epoxy resins. Polym J. 2009;41(11):988–992.

[209] Hughes-Brittain NF, Qiu L, Wang W, et al. Photoembossing of surface relief structures in polymer films for biomedical applications. J Biomed Mater Res - Part B Appl Biomater. 2014;102(2):214–220. DOI:10.1002/jbm.b.32997 23908051

[210] Picot OT, Alcalá R, Sánchez C, et al. Manufacturing of surface relief structures in moving substrates using photoembossing and pulsed-interference holography. Macromol Mater Eng. 2013;298(1):33–37. DOI:10.1002/mame.201100433

[211] Hashimoto S, Aizawa M, Akamatsu N, et al. Simultaneous formation behaviour of surface structures and molecular alignment by patterned photopolymerisation. Liq Cryst. 2019;46(13–14):1995–2002. DOI:10.1080/02678292.2019.1610980

[212] Ubukata T, Yamamoto S, Moriya Y, et al. Photo-triggered surface relief of polystyrene films-highly photo-sensitive formation by the addition of a benzophenone derivative. J Photopolym Sci Technol. 2012;25(5):675–678. DOI:10.2494/photopolymer.25.675

[213] Fiorini C, Prudhomme N, De Veyrac G, et al. Molecular migration mechanism for laser induced surface relief grating formation. Synth Met. 2000;115(1–3):121–125. DOI:10.1016/S0379-6779(00)00332-5

[214] Yager KG, Barrett CJ. All-optical patterning of azo polymer films. Curr Opin Solid State Mater Sci. 2001;5(6):487–494.

[215] Baldus O, Zilker SJ. Surface relief gratings in photoaddressable polymers generated by cw holography. Appl Phys B Lasers Opt. 2001;72(4):425–427.

[216] Hubert C, Fiorini-Debuisschert C, Maurin I, et al. Spontaneous patterning of hexagonal structures in an azo-polymer using light-controlled mass transport. Adv Mater. 2002;14(10):729–732. DOI:10.1002/1521-4095(20020517)14:10<729:AID-ADMA729>3.0.CO;2-1

[217] Sumaru K, Fukuda T, Kimura T, et al. Photoinduced surface relief formation on azopolymer films: a driving force and formed relief profile. J Appl Phys. 2002;91(5):3421–3430. DOI:10.1063/1.1432482

[218] Ciuchi F, Mazzulla A, Carbone G, et al. Complex structures of surface relief induced by holographic recording in azo-dye-doped elastomer thin films. Macromolecules. 2003;36(15):5689–5693. DOI:10.1021/ma021772d

[219] Ivanov M, Rochon P. Infrared-laser-induced periodic surface structure in azo-dye polymer. Appl Phys Lett. 2004;84(22):4511–4513.

[220] Yu H, Okano K, Shishido A, et al. Enhancement of surface-relief gratings recorded on amphiphilic liquid-crystalline diblock copolymer by nanoscale phase separation. Adv Mater. 2005;17(18):2184–2188. DOI:10.1002/adma.200500346

[221] Oliveira ON, Dos Santos DS, Balogh DT, et al. Optical storage and surface-relief gratings in azobenzene-containing nanostructured films. Adv Colloid Interface Sci. 2005;116(1–3):179–192. DOI:10.1016/j.cis.2005.05.008 16257385

[222] Ubukata T, Isoshima T, Hara M. Wavelength-programmable organic distributed-feedback laser based on a photoassisted polymer-migration system. Adv Mater. 2005;17(13):1630–1633.

[223] Kim DY, Tripathy SK, Li L, et al. Laser-induced holographic surface relief gratings on nonlinear optical polymer films. Appl Phys Lett. 1995;66(10):1166. DOI:10.1063/1.113845

[224] You F, Paik MY, Häckel M, et al. Control and suppression of surface relief gratings in liquid-crystalline perfluoroalkyl–azobenzene polymers. Adv Funct Mater. 2006;16(12):1577–1581. DOI:10.1002/adfm.200500711

[225] Ubukata T, Isoshima T, Hara M. Wavelength programmable organic distributed feedback laser using a photoinduced surface relief grating. Mol Cryst Liq Cryst. 2006;445(1):[269/[559]–273/[563].

[226] He Y, Yin J, Che P, et al. Epoxy-based polymers containing methyl-substituted azobenzene chromophores and photoinduced surface relief gratings. Eur Polym J. 2006;42(2):292–301. DOI:10.1016/j.eurpolymj.2005.07.019

[227] Yager KG, Barrett CJ. Photomechanical surface patterning in azo-polymer materials. Macromolecules. 2006;39(26):9320–9326.

[228] Kulikovska O, Goldenberg LM, Kulikovsky L, et al. Smart ionic sol−gel-based azobenzene materials for optical generation of microstructures. Chem Mater. 2008;20(10):3528–3534. DOI:10.1021/cm800106x

[229] Gao J, He Y, Liu F, et al. Azobenzene-containing supramolecular side-chain polymer films for laser-induced surface relief gratings. Chem Mater. 2007;19(16):3877–3881. DOI:10.1021/cm0707197

[230] Yu H, Naka Y, Shishido A, et al. Well-defined liquid-crystalline diblock copolymers with an azobenzene moiety: synthesis, photoinduced alignment and their holographic properties. Macromolecules. 2008;41(21):7959–7966. DOI:10.1021/ma801077g

[231] Lee S, Jeong YC, Park JK. Unusual surface reliefs from photoinduced creeping and aggregation behavior of azopolymer. Appl Phys Lett. 2008;93(3):031912.

[232] Guo M, Xu Z, Wang X. Photofabrication of two-dimensional quasi-crystal patterns on UV-curable molecular azo glass films. Langmuir. 2008;24(6):2740–2745. 18237214 10.1021/la703091x

[233] He Y, Gu X, Guo M, et al. Dendritic azo compounds as a new type amorphous molecular material with quick photoinduced surface-relief-grating formation ability. Opt Mater. 2008;31(1):18–27. DOI:10.1016/j.optmat.2008.01.003

[234] Rochon P, Batalla E, Natansohn A. Optically induced surface gratings on azoaromatic polymer films. Appl Phys Lett. 1995;136(2):136.

[235] Lev B, Chernyshuk SB, Yamamoto T, et al. Photochemical switching between colloidal photonic crystals at the nematic-air interface. Phys Rev E. 2008;78(2):020701. DOI:10.1103/PhysRevE.78.020701 18850778

[236] Zhang Y, Zhang W, Chen X, et al. Synthesis of novel three-arm star azo side-chain liquid crystalline polymer *via* ATRP and photoinduced surface relief gratings. J Polym Sci Part A. 2008;46(3):777–789. DOI:10.1002/pola.22423

[237] Zhang Q, Wang X, Barrett CJ, et al. Spacer-free ionic dye−polyelectrolyte complexes: influence of molecular structure on liquid crystal order and photoinduced motion. Chem Mater. 2009;21(14):3216–3227. DOI:10.1021/cm900810r

[238] Goldenberg LM, Kulikovsky L, Kulikovska O, et al. New materials with detachable azobenzene: effective, colourless and extremely stable surface relief gratings. J Mater Chem. 2009;19(43):8068–8071. DOI:10.1039/b918130j

[239] Vapaavuori J, Priimagi A, Kaivola M. Photoinduced surface-relief gratings in films of supramolecular polymer–bisazobenzene complexes. J Mater Chem. 2010;20(25):5260–5264.

[240] Yin J, Ye G, Wang X. Self-structured surface patterns on molecular azo glass films induced by laser light irradiation. Langmuir. 2010;26(9):6755–6761. 20000616 10.1021/la9041056

[241] Ambrosio A, Maddalena P, Carella A, et al. Two-photon induced self-structuring of polymeric films based on Y-shape azobenzene chromophore. J Phys Chem C. 2011;115(28):13566–13570. DOI:10.1021/jp200050h

[242] Schuh C, Lomadze N, Rühe J, et al. Photomechanical degrafting of azo-functionalized poly(methacrylic acid) (PMAA) brushes. J Phys Chem B. 2011;115(35):10431–10438. DOI:10.1021/jp2041229 21790176

[243] Lomadze N, Kopyshev A, Rühe J, et al. Light-induced chain scission in photosensitive polymer brushes. Macromolecules. 2011;44(18):7372–7377. DOI:10.1021/ma201016q

[244] Wang X, Yin J, Wang X. Photoinduced self-structured surface pattern on a molecular azo glass film: structure–property relationship and wavelength correlation. Langmuir. 2011;27(20):12666–12676. 21875109 10.1021/la2027253

[245] Barrett CJ, Natansohn AL, Rochon PL. Mechanism of optically inscribed high-efficiency diffraction gratings in azo polymer films. J Phys Chem. 1996;100(21):8836–8842.

[246] Wang X, Yin J, Wang X. Self-structured surface patterns on epoxy-based azo polymer films induced by laser light irradiation. Macromolecules. 2011;44(17):6856–6867.

[247] Ahmed R, Priimagi A, Faul CFJ, et al. Redox-active, organometallic surface-relief gratings from azobenzene-containing polyferrocenylsilane block copolymers. Adv Mater. 2012;24(7):926–931. DOI:10.1002/adma.201103793 22250040

[248] Ambrosio A, Marrucci L, Borbone F, et al. Light-induced spiral mass transport in azo-polymer films under vortex-beam illumination. Nat Commun. 2012;3(1):989. DOI:10.1038/ncomms1996 22871808 PMC3432464

[249] Jacquart A, Morin E, Yang F, et al. Influence of extrinsic and intrinsic parameters onto the formation of surface relief gratings in polar azo molecular glasses. Dyes Pigm. 2012;92:790–797.

[250] Priimagi A, Cavallo G, Forni A, et al. Halogen bonding versus hydrogen bonding in driving self-assembly and performance of light-responsive supramolecular polymers. Adv Funct Mater. 2012;22(12):2572–2579. DOI:10.1002/adfm.201200135

[251] Priimagi A, Saccone M, Cavallo G, et al. Photoalignment and surface-relief-grating formation are efficiently combined in low-molecular-weight halogen-bonded complexes. Adv Mater. 2012;24(44):345–352. DOI:10.1002/adma.201204060 23081696

[252] Koskela JE, Vapaavuori J, Hautala J, et al. Surface-relief gratings and stable birefringence inscribed using light of broad spectral range in supramolecular polymer-bisazobenzene complexes. J Phys Chem C. 2012;116(3):2363–2370. DOI:10.1021/jp210706n

[253] Schab-Balcerzak E, Sobolewska A, Stumpe J, et al. Surface relief gratings in azobenzene supramolecular systems based on polyimides. Opt Mater. 2012;35(2):155–167. DOI:10.1016/j.optmat.2012.07.029

[254] Ishikawa D, Ito E, Han M, et al. Effect of the steric molecular structure of azobenzene on the formation of self-assembled monolayers with a photoswitchable surface morphology. Langmuir. 2013;29(14):4622–4631. DOI:10.1021/la302552v 23249363

[255] Ambrosio A, Maddalena P, Marrucci L. Molecular model for light-driven spiral mass transport in azopolymer films. Phys Rev Lett. 2013;110(14):1–5. 10.1103/PhysRevLett.110.146102 25167010

[256] Darracq B, Chaput F, Lahlil K, et al. Photoinscription of surface relief gratings on azo-hybrid gels. Adv Mater. 1998;10(14):1133–1136. DOI:10.1002/(SICI)1521-4095(199810)10:14<1133:AID-ADMA1133>3.0.CO;2-F

[257] Vapaavuori J, Priimagi A, Soininen AJ, et al. Photoinduced surface patterning of azobenzene-containing supramolecular dendrons, dendrimers and dendronized polymers. Opt Mater Express. 2013;3(6):711. DOI:10.1364/OME.3.000711

[258] Yadavalli NS, Linde F, Kopyshev A, et al. Soft matter beats hard matter: rupturing of thin metallic films induced by mass transport in photosensitive polymer films. ACS Appl Mater Interfaces. 2013;5(16):7743–7747. DOI:10.1021/am400682w 23895573

[259] König T, Tsukruk VV, Santer S. Controlled topography change of subdiffraction structures based on photosensitive polymer films induced by surface plasmon polaritons. ACS Appl Mater Interfaces. 2013;5(13):6009–6016. 23701312 10.1021/am400712r

[260] Tomczyk J, Sobolewska A, Nagy ZT, et al. Photo- and thermal-processing of azobenzene-containing star-shaped liquid crystals. J Mater Chem C. 2013;1(5):924–932. DOI:10.1039/C2TC00627H

[261] Florio GD, Bründermann E, Yadavalli NS, et al. Graphene multilayer as nanosized optical strain gauge for polymer surface relief gratings. Nano Lett. 2014;14(10):5754–5760. DOI:10.1021/nl502631s 25244634

[262] Koskela JE, Vapaavuori J, Ras RHA, et al. Light-driven surface patterning of supramolecular polymers with extremely low concentration of photoactive molecules. ACS Macro Lett. 2014;3(11):1196–1200. DOI:10.1021/mz500616q 35610824

[263] Wei R, Xu Z, Wang X. Epoxy-based azo polymer for photofabricating surface-relief quasi-crystal structures. Opt Mater Express. 2015;5(6):1348.

[264] Kopyshev A, Galvin CJ, Patil RR, et al. Light-induced reversible change of roughness and thickness of photosensitive polymer brushes. ACS Appl Mater Interfaces. 2016;8(29):19175–19184. DOI:10.1021/acsami.6b06881 27351592

[265] Wang X, Vapaavuori J, Wang X, et al. Influence of supramolecular interaction type on photoresponsive azopolymer complexes: a surface relief grating formation study. Macromolecules. 2016;49(13):4923–4934. DOI:10.1021/acs.macromol.6b01009

[266] Bedrov D, Hooper JB, Glaser MA, et al. Photoinduced and thermal relaxation in surface-grafted azobenzene-based monolayers: a molecular dynamics simulation study. Langmuir. 2016;32(16):4004–4015. DOI:10.1021/acs.langmuir.6b00120 27027147

[267] Pedersen TG, Johansen PM, Holme NCR, et al. Mean-field theory of photoinduced formation of surface reliefs in side-chain azobenzene polymers. Phys Rev Lett. 1998;80(1):89–92. DOI:10.1103/PhysRevLett.80.89

[268] Frascella F, Angelini A, Ricciardi S, et al. Surface-relief formation in azo-polyelectrolyte layers with a protective polymer coating. Opt Mater Express. 2016;6(2):444. DOI:10.1364/OME.6.000444

[269] Kim CB, Wistrom JC, Ha H, et al. Marangoni instability driven surface relief grating in an azobenzene-containing polymer film. Macromolecules. 2016;49(18):7069–7076. DOI:10.1021/acs.macromol.6b01848

[270] Noga J, Sobolewska A, Bartkiewicz S, et al. Periodic surface structures induced by a single laser beam irradiation. Macromol Mater Eng. 2017;302(2):1600329. DOI:10.1002/mame.201600329

[271] Lomadze N, Kopyshev A, Bargheer M, et al. Mass production of polymer nano-wires filled with metal nano-particles. Sci Rep. 2017;7(1):8506. DOI:10.1038/s41598-017-08153-0 28819103 PMC5561068

[272] Hendrikx M, Ter Schiphorst J, van Heeswijk EPA, et al. Re- and preconfigurable multistable visible light responsive surface topographies. Small. 2018;14(50):1803274. DOI:10.1002/smll.201803274 30353702

[273] Vapaavuori J, Bazuin CG, Priimagi A. Supramolecular design principles for efficient photoresponsive polymer–azobenzene complexes. J Mater Chem C. 2018;6(9):2168–2188.

[274] Oscurato SL, Salvatore M, Borbone F, et al. Computer-generated holograms for complex surface reliefs on azopolymer films. Sci Rep. 2019;9(1):6775. DOI:10.1038/s41598-019-43256-w 31043674 PMC6494893

[275] Kim K, Park H, Park KJ, et al. Light-directed soft mass migration for micro/nanophotonics. Adv Opt Mater. 2019;7(16):1900074. DOI:10.1002/adom.201900074

[276] Jelken J, Santer S. Light induced reversible structuring of photosensitive polymer films. RSC Adv. 2019;9(35):20295–20305. 35514701 10.1039/c9ra02571e PMC9065545

[277] Kumar J, Li L, Jiang XL, et al. Gradient force: the mechanism for surface relief grating formation in azobenzene functionalized polymers. Appl Phys Lett. 1998;72:2096–2098.

[278] Vapaavuori J, Stimpson TC, Moran-Mirabal JM. Dynamically evolving surface patterns through light-triggered wrinkling erasure. Langmuir. 2019;35(4):875–881. 30532978 10.1021/acs.langmuir.8b03542

[279] Spiridon MC, Demazy N, Brochon C, et al. Optical alignment of Si-containing nanodomains formed by photoresponsive amorphous block copolymer thin films. Macromolecules. 2020;53(1):68–77. DOI:10.1021/acs.macromol.9b01551

[280] Salvatore M, Borbone F, Oscurato SL. Deterministic realization of quasicrystal surface relief gratings on thin azopolymer films. Adv Mater Interfaces. 2020;7(11):1902118.

[281] Miniewicz A, Sobolewska A, Piotrowski W, et al. Thermocapillary marangoni flows in azopolymers. Materials. 2020;13(11):2464. DOI:10.3390/ma13112464 32481714 PMC7321112

[282] Chen J, Xu T, Zhao W, et al. Photoresponsive thin films of well-synthesized azobenzene side-chain liquid crystalline polynorbornenes as command surface for patterned graphic writing. Polymer. 2021;218:123492.

[283] Feng W, Chu L, de Rooij MB, et al. Photoswitching between water-tolerant adhesion and swift release by inverting liquid crystal fingerprint topography. Adv Sci. 2021;8(8):2004051. DOI:10.1002/advs.202004051 PMC8061410 33898189

[284] Bian S, Li L, Kumar J, et al. Single laser beam-induced surface deformation on azobenzene polymer films. Appl Phys Lett. 1998;73:1817–1819.

[285] Viswanathan NK, Balasubramanian S, Li L, et al. Surface-initiated mechanism for the formation of relief gratings on azo-polymer films. J Phys Chem B. 1998;102(31):6064–6070. DOI:10.1021/jp981425z

[286] Fong WK, Malic N, Evans RA, et al. Alkylation of spiropyran moiety provides reversible photo-control over nanostructured soft materials. Biointerphases. 2012;7(1):3–7. DOI:10.1007/s13758-011-0003-9 22589046

[287] Ito M, Ubukata T. Photoconstruction of a microrelief in a photochromic crystalline spirooxazine film. Chem Lett. 2019;48(1):32–35.

[288] Mele E, Pisignano D, Varda M, et al. Smart photochromic gratings with switchable wettability realized by green-light interferometry. Appl Phys Lett. 2006;88(20):203124. DOI:10.1063/1.2198509

[289] Ubukata T, Takahashi K, Yokoyama Y. Photoinduced surface relief structures formed on polymer films doped with photochromic spiropyrans. J Phys Org Chem. 2007;20(11):981–984.

[290] Ubukata T, Fujii S, Arimatsu K, et al. Phototriggered micromanufacturing using photoresponsive amorphous spirooxazine films. J Mater Chem. 2012;22(29):14410–14417. DOI:10.1039/c2jm32149a

[291] Ubukata T, Yamaguchi S, Yokoyama Y. Photoinduced surface relief structures formed on polymer films mixed with diarylethenes. Chem Lett. 2007;36(10):1224–1225.

[292] Kikuchi A, Harada Y, Yagi M, et al. Photoinduced diffusive mass transfer in *o*-Cl-HABI amorphous thin films. Chem Commun. 2010;46(13):2262–2264. DOI:10.1039/b919180a 20234926

[293] Park JW, Nagano S, Yoon SJ, et al. High contrast fluorescence patterning in cyanostilbene-based crystalline thin films: crystallization-induced mass flow *via* a photo-triggered phase transition. Adv Mater. 2014;26(9):1354–1359. DOI:10.1002/adma.201304250 24734301

[294] Zhao D, Xu Z, Wang G, et al. Formation of surface relief gratings with homeotropically oriented photopolymer from a photocross-linkable organic monomer. Phys Chem Chem Phys. 2010;12(7):1436–1439. DOI:10.1039/B919659E 20126755

[295] Ono H, Emoto A, Kawatsuki N, et al. Self-organized phase gratings in photoreactive polymer liquid crystals. Appl Phys Lett. 2003;82(9):1359–1361. DOI:10.1063/1.1557327

[296] Ono H, Hatayama A, Emoto A, et al. Migration induced reorientation and anisotropic grating formation in photoreactive polymer liquid crystals. Opt Mater. 2007;30(2):248–254. DOI:10.1016/j.optmat.2006.11.048

[297] Emoto A, Matsumoto T, Yamashita A, et al. Large birefringence and polarization holographic gratings formed in photocross-linkable polymer liquid crystals comprising bistolane mesogenic side groups. J Appl Phys. 2009;106(7):073505. DOI:10.1063/1.3234385

[298] Kawatsuki N, Matsushita H, Kondo M, et al. Photoinduced reorientation and polarization holography in a new photopolymer with 4-methoxy-*N*-benzylideneaniline side groups. APL Mater. 2013;1(2):022103. DOI:10.1063/1.4818003

[299] Okano K, Ogino S, Kawamoto M, et al. Mass migration on a polymer surface caused by photoinduced molecular rotation. Chem Commun. 2011;47(43):11891–11893. DOI:10.1039/c1cc14375a 21975651

[300] Ubukata T, Seki T, Ichimura K. Surface relief gratings in host-guest supramolecular materials. Adv Mater. 2000;12(22):1675–1678.

[301] Zettsu N, Ubukata T, Seki T, et al. Soft crosslinkable azo polymer for rapid surface relief formation and persistent fixation. Adv Mater. 2001;13(22):1693–1697. DOI:10.1002/1521-4095(200111)13:22<1693:AID-ADMA1693>3.0.CO;2-2

[302] Zettsu N, Ogasawara T, Arakawa R, et al. Highly photosensitive surface relief gratings formation in a liquid crystalline azobenzene polymer: new implications for the migration process. Macromolecules. 2007;40:4607–4613.

[303] Zettsu N, Ogasawara T, Mizoshita N, et al. Photo-triggered surface relief grating formation in supramolecular liquid crystalline polymer systems with detachable azobenzene units. Adv Mater. 2008;20(3):516–521. DOI:10.1002/adma.200701110

[304] Nishizawa K, Nagano S, Seki T. Novel liquid crystalline organic−inorganic hybrid for highly sensitive photoinscriptions. Chem Mater. 2009;21(13):2624–2631.

[305] Nishizawa K, Nagano S, Seki T. Micropatterning of titanium oxide film *via* phototactic mass transport. J Mater Chem. 2009;19(39):7191–7194.

[306] Li W, Nagano S, Seki T. Photo-crosslinkable liquid-crystalline azo-polymer for surface relief gratings and persistent fixation. New J Chem. 2009;33(6):1343–1348.

[307] Isayama J, Nagano S, Seki T. Phototriggered mass migrating motions in liquid crystalline azobenzene polymer films with systematically varied thermal properties. Macromolecules. 2010;43(9):4105–4112.

[308] Li W, Dohi T, Hara M, et al. Phototriggered mass migration consorted with surface dewetting in thin films of a liquid crystalline azobenzene-containing dendrimer. Macromolecules. 2012;45(16):6618–6627. DOI:10.1021/ma301170x

[309] Seki T. Meso- and microscopic motions in photoresponsive liquid crystalline polymer films. Macromol Rapid Commun. 2014;35:271–290. 24343758 10.1002/marc.201300763

[310] Mitsui S, Nagano S, Hara M, et al. SRG inscription in supramolecular liquid crystalline polymer film: replacement of mesogens. Crystals. 2017;7(2):52. DOI:10.3390/cryst7020052

[311] Beppu K, Nagashima Y, Hara M, et al. Photoalignment of vertically oriented microphase separated lamellae in LC–LC diblock copolymer thin film. Macromol Rapid Commun. 2017;38(13):1600659. DOI:10.1002/marc.201600659 28338244

[312] Zettsu N, Ubukata T, Seki T, et al. Azo polymers with oligo(ethylene oxide) side chain for rapid surface relief formation. J Photopolym Sci Technol. 2001;14(2):193–194. DOI:10.2494/photopolymer.14.193

[313] Kitamura I, Oishi K, Hara M, et al. Photoinitiated marangoni flow morphing in a liquid crystalline polymer film directed by super-inkjet printing patterns. Sci Rep. 2019;9(1):2556. DOI:10.1038/s41598-019-38709-1 30796238 PMC6385296

[314] Kitamura I, Kato K, Berk RB, et al. Photo-triggered large mass transport driven only by a photoresponsive surface skin layer. Sci Rep. 2020;10(1):12664. DOI:10.1038/s41598-020-69605-8 32728143 PMC7391747

[315] Yamakado R, Kitamura I, Hara M, et al. Photoisomerization-induced patterning of ion-pairing materials based on anionic azobenzene and its complex with a fluorescent π-electronic system. Chem Commun. 2021;57(35):4287–4290. DOI:10.1039/D0CC07640F 33913948

[316] Zhang D, Liu D, Ubukata T, et al. Unconventional approaches to light-promoted dynamic surface morphing on polymer films. Bull Chem Soc Jpn. 2022;95(1):138–162. DOI:10.1246/bcsj.20210348

[317] Ubukata T, Seki T, Ichimura K. Surface relief grating in hybrid films composed of azobenzene polymer and liquid crystal molecule. Colloids Surf A Physicochem Eng Asp. 2002;198–200:113–117.

[318] Ubukata T, Hara M, Seki T. Photogeneration of surface relief gratings in azobenzene polymer/liquid crystal hybrid films. Mol Cryst Liq Cryst. 2002;377(1):173–176.

[319] Ubukata T, Hara M, Ichimura K, et al. Phototactic mass transport in polymer films for micropatterning and alignment of functional materials. Adv Mater. 2004;16(3):220–223. DOI:10.1002/adma.200305535

[320] Zettsu N, Ubukata T, Seki T. Two-dimensional manipulation of poly(3-dodecylthiophene) using light-driven instant mass migration as a molecular conveyer. Japanese J Appl Physics. 2004;43(No. 9A/B):L1169–1171. Part 2 Lett. DOI:10.1143/JJAP.43.L1169.

[321] Zettsu N, Seki T. Highly efficient photogeneration of surface relief structure and its immobilization in cross-linkable liquid crystalline azobenzene polymers. Macromolecules. 2004;37(23):8692–8698.

[322] Ubukata T, Higuchi T, Zettsu N, et al. Spontaneous motion observed in highly sensitive surface relief formation system. Colloids Surf A Physicochem Eng Asp. 2005;257-258:123–126.

[323] Seki T. Photoresponsive self-assembly motions in polymer thin films. Curr Opin Solid State Mater Sci. 2006;10(5–6):241–248.

[324] Kopyshev A, Kanevche K, Lomadze N, et al. Light-induced structuring of photosensitive polymer brushes. ACS Appl Polym Mater. 2019;1(11):3017–3026. DOI:10.1021/acsapm.9b00705

[325] Yoneyama S, Yamamoto T, Tsutsumi O, et al. High-performance material for holographic gratings by means of a photoresponsive polymer liquid crystal containing a tolane moiety with high birefringence. Macromolecules. 2002;35(23):8751–8758. DOI:10.1021/ma020886m

[326] Yamamoto T, Hasegawa M, Kanazawa A, et al. Holographic gratings and holographic image storage *via* photochemical phase transitions of polymer azobenzene liquid-crystal films. J Mater Chem. 2000;10(2):337–342. DOI:10.1039/a905501k

[327] Yamamoto T, Yoneyama S, Tsutsumi O, et al. Holographic gratings in the optically isotropic state of polymer azobenzene liquid-crystal films. J Appl Phys. 2000;88(5):2215–2220. DOI:10.1063/1.1287761

[328] Shishido A, Ishiguro M, Ikeda T. Circular arrangement of mesogens induced in Bragg-type polarization holograms of thick azobenzene copolymer films with a tolane moiety. Chem Lett. 2007;36(9):1146–1147.

[329] Ishiguro M, Sato D, Shishido A, et al. Bragg-type polarization gratings formed in thick polymer films containing azobenzene and tolane moieties. Langmuir. 2007;23(1):332–338. DOI:10.1021/la061587j 17190523

[330] Bang CU, Shishido A, Ikeda T. Azobenzene liquid-crystalline polymer for optical switching of grating waveguide couplers with a flat surfacea. Macromol Rapid Commun. 2007;28(9):1040–1044.

[331] Ikeda T, Yoneyama S, Yamamoto T, et al. Holographic grating by means of polymer liquid crystals. J Inf Disp. 2001;2(3):6–12. DOI:10.1080/15980316.2001.9651860

[332] Hasegawa M, Yamamoto T, Kanazawa A, et al. Real-time holographic grating by means of photoresponsive polymer liquid crystals with a flexible siloxane spacer in the side chain. J Mater Chem. 1999;9(11):2765–2769. DOI:10.1039/a903948a

[333] Hasegawa M, Yamamoto T, Kanazawa A, et al. A dynamic grating using a photochemical phase transition of polymer liquid crystals containing azobenzene derivatives. Adv Mater. 1999;11(8):675–677. DOI:10.1002/(SICI)1521-4095(199906)11:8<675:AID-ADMA675>3.0.CO;2-Z

[334] Yamamoto T, Hasegawa M, Kanazawa A, et al. Phase-type gratings formed by photochemical phase transition of polymer azobenzene liquid crystals: enhancement of diffraction efficiency by spatial modulation of molecular alignment. J Phys Chem B. 1999;103(45):9873–9878. DOI:10.1021/jp992172s

[335] Tsutsumi O, Ikeda T. Photochemical modulation of alignment of liquid crystals and photonic applications. Curr Opin Solid State Mater Sci. 2002;6(6):563–568.

[336] Shishido A, Cha H-B, Ikeda T. Rewritable bragg holograms of azobenzene polymers with fast response. Liq Cryst. 2009 XIII(7414):74140S.

[337] Ishii N, Kato T, Abe J. A real-time dynamic holographic material using a fast photochromic molecule. Sci Rep. 2012;2(1):819. 23139865 10.1038/srep00819 PMC3492843

[338] Kobayashi Y, Abe J. Real-time dynamic hologram of a 3D object with fast photochromic molecules. Adv Opt Mater. 2016;4(9):1354–1357.

[339] Wang H, Pumera M. Fabrication of micro/nanoscale motors. Chem Rev. 2015;115(16):8704–8735. 26234432 10.1021/acs.chemrev.5b00047

[340] Tu Y, Peng F, Wilson DA. Motion manipulation of micro- and nanomotors. Adv Mater. 2017;29(39):1701970. 10.1002/adma.201701970 28841755

[341] Eskandarloo H, Kierulf A, Abbaspourrad A. Light-harvesting synthetic nano- and micromotors: a review. Nanoscale. 2017;9:12218–12230. 28809422 10.1039/c7nr05166b

[342] Xu L, Mou F, Gong H, et al. Light-driven micro/nanomotors: from fundamentals to applications. Chem Soc Rev. 2017;46(22):6905–6926. DOI:10.1039/C7CS00516D 28949354

[343] Kuwahara Y, Oda T, Kim S, et al. Photo-responsive traveling of small-particles modified with azobenzene groups as molecular motors in a liquid crystal. Mater Lett. 2016;181:257–260.

[344] Uchida E, Azumi R, Norikane Y. Light-induced crawling of crystals on a glass surface. Nat Commun. 2015;6(1):7310. 26084483 10.1038/ncomms8310 PMC4557305

[345] Nakano H. Direction control of photomechanical bending of a photochromic molecular fiber. J Mater Chem. 2010;20(11):2071–2074.

[346] Norikane Y, Hayashino M, Ohnuma M, et al. Photo-induced crawling motion of azobenzene crystals on modified gold surfaces. Langmuir. 2021;37(48):14177–14185. DOI:10.1021/acs.langmuir.1c02494 34808058

[347] Muto M, Ayako Y, Yamamoto K, et al. Photochemical migration of liquid column in a glass tube. Eur Phys J Spec Top. 2017;226(6):1199–1205. DOI:10.1140/epjst/e2016-60217-y

[348] Norikane Y, Hayashino M, Ohnuma M, et al. Effect of surface properties on the photo-induced crawling motion of azobenzene crystals on glass surfaces. Front Chem. 2021;9:684767. 34422758 10.3389/fchem.2021.684767 PMC8374144

[349] Suzuki M, Nakano H. Moving fragments of photochromic molecuar glass of 4-[bis(9,9-dimethylfluoren-2-yl)amino]-4′-cyanoazobenzene. J Photopolym Sci Technol. 2012;25(2):159–160.

[350] Lin G, Richardson JJ, Ahmed H, et al. Programmable phototaxis of metal–phenolic particle microswimmers. Adv Mater. 2021;33(13):2006177. DOI:10.1002/adma.202006177 33634513

[351] Nakano H, Suzuki M. Photoinduced mass flow of photochromic molecular materials. J Mater Chem. 2012;22(9):3702–3704.

[352] Ichikawa R, Nakano H. Photoinduced change in the shape of azobenzene-based molecular glass particles fixed in agar gel. RSC Adv. 2016;6(43):36761–36765.

[353] Matsubara M, Ukai H, Kuragano M, et al. Chiral photomechanical behavior of achiral azobenzene-based molecular glass particles fixed in agar gel. Chem Lett. 2022;51(5):493–496. DOI:10.1246/cl.220054

[354] Liu GL, Kim J, Lu YU, et al. Optofluidic control using photothermal nanoparticles. Nat Mater. 2006;5(1):27–32. DOI:10.1038/nmat1528 16362056

[355] Kaneko S, Asakura K, Banno T. Phototactic behavior of self-propelled micrometer-sized oil droplets in a surfactant solution. Chem Commun. 2017;53(14):2237–2240. 10.1039/c6cc09236e 28144652

[356] Nitta K, Tsukahara T. Numerical demonstration of in-tube liquid-column migration driven by photoisomerization. Micromach. 2018;9(10):533. 10.3390/mi9100533 PMC6215142 30424466

[357] Eelkema R, Pollard MM, Katsonis N, et al. Rotational reorganization of doped cholesteric liquid crystalline films. J Am Chem Soc. 2006;128(44):14397–14407. DOI:10.1021/ja065334o 17076514

[358] Kausar A, Nagano H, Okada S, et al. Micro manipulation with optical responsive cholesteric and compensated nematic liquid crystal. Mol Cryst Liq Cryst. 2009;513(1):122–130. DOI:10.1080/15421400903196112

[359] Mafy NN, Kim Y, Thomas R, et al. Molecular crankshaft effect converting piston-like molecular motion to continuous rotation of macro objects. ACS Appl Mater Interfaces. 2019;11(16):15097–15102. DOI:10.1021/acsami.9b03706 30931554

[360] Kim Y, Mafy NN, Maisonneuve S, et al. Glycomacrocycle-based azobenzene derivatives as chiral dopants for photoresponsive cholesteric liquid crystals. ACS Appl Mater Interfaces. 2020;12(46):52146–52155. DOI:10.1021/acsami.0c14880 33141559

[361] Eelkema R, Pollard MM, Vicario J, et al. Nanomotor rotates microscale objects. Nature. 2006;440(7081):163. DOI:10.1038/440163a 16525460

[362] Tamaoki N, Kamei T. Reversible photo-regulation of the properties of liquid crystals doped with photochromic compounds. J Photochem Photobiol C Photochem Rev. 2010;11(2–3):47–61.

[363] Jau HC, Lin TH, Chen YY, et al. Direction switching and beam steering of cholesteric liquid crystal gratings. Appl Phys Lett. 2012;100(13):131909. DOI:10.1063/1.3698384

[364] Thomas R, Yoshida Y, Akasaka T, et al. Influence of a change in helical twisting power of photoresponsive chiral dopants on rotational manipulation of micro-objects on the surface of chiral nematic liquid crystalline films. Chem - A Eur J. 2012;18(39):12337–12348. DOI:10.1002/chem.201200836 22907600

[365] Ma S, Kuwahara Y, Nagano H, et al. Photo-controlled manipulation of micrometer-scale objects on polyethyleneglycol thin films with azobenzene compounds. Mol Cryst Liq Cryst. 2014;601(1):126–133. DOI:10.1080/15421406.2014.944382

[366] Kim Y, Tamaoki N. A photoresponsive planar chiral azobenzene dopant with high helical twisting power. J Mater Chem C. 2014;2(43):9258–9264.

[367] Kim Y, Tamaoki N. Asymmetric dimers of chiral azobenzene dopants exhibiting unusual helical twisting power upon photoswitching in cholesteric liquid crystals. ACS Appl Mater Interfaces. 2016;8(7):4918–4926. 26815738 10.1021/acsami.5b11888

[368] Wu ZL, Wang ZJ, Keller P, et al. Light responsive microstructured surfaces of liquid crystalline network with shape memory and tunable wetting behaviors. Macromol Rapid Commun. 2016;37(4):311–317. DOI:10.1002/marc.201500533 26676211

[369] Kim Y, Frigoli M, Vanthuyne N, et al. A helical naphthopyran dopant for photoresponsive cholesteric liquid crystals. Chem Commun. 2017;53(1):200–203. DOI:10.1039/C6CC08667E 27917417

[370] Mathews M, Tamaoki N. Planar chiral azobenzenophanes as chiroptic switches for photon mode reversible reflection color control in induced chiral nematic liquid crystals. J Am Chem Soc. 2008;130(34):11409–11416. 18680250 10.1021/ja802472t

[371] Li Y, Li Q. Photochemically reversible and thermally stable axially chiral diarylethene switches. Org Lett. 2012;14(17):4362–4365. 22909002 10.1021/ol3018165

[372] Kausar A, Nagano H, Kuwahara Y, et al. Photocontrolled manipulation of a microscale object: a rotational or translational mechanism. Chem Eur J. 2011;17(2):508–515. DOI:10.1002/chem.201001238 21207567

[373] Ichimura K, Oh S-K, Nakagawa M. Light-driven motion of liquids on a photoresponsive surface. Science. 2000;288:1624–1626. 10834837 10.1126/science.288.5471.1624

[374] Oh SK, Nakagawa M, Ichimura K. Photocontrol of liquid motion on an azobenzene monolayer. J Mater Chem. 2002;12(8):2262–2269.

[375] Monobe H, Ohzono T, Akiyama H, et al. Manipulation of liquid filaments on photoresponsive microwrinkles. ACS Appl Mater Interfaces. 2012;4(4):2212–2217. DOI:10.1021/am300225m 22448895

[376] Zhu F, Tan S, Dhinakaran MK, et al. The light-driven macroscopic directional motion of a water droplet on an azobenzene–calix[4]arene modified surface. Chem Commun. 2020;56(74):10922–10925. DOI:10.1039/D0CC00519C 32808622

[377] De Jong E, Kremer R, Liu L, et al. Mechanowetting drives droplet and fluid transport on traveling surface waves generated by light-responsive liquid crystal polymers. Phys Fluids. 2021;33(6):063307. DOI:10.1063/5.0050864

[378] Yamamoto T, Yamamoto J, Lev BI, et al. Light-induced assembly of tailored droplet arrays in nematic emulsions. Appl Phys Lett. 2002;81(12):2187–2189. DOI:10.1063/1.1508816

[379] Diguet A, Guillermic RM, Magome N, et al. Photomanipulation of a droplet by the chromocapillary effect. Angew Chem Int Ed. 2009;48(49):9281–9284. DOI:10.1002/anie.200904868 19890927

[380] Kausar A, Nagano H, Ogata T, et al. Photocontrolled translational motion of a microscale solid object on azobenzene-doped liquid-crystalline films. Angew Chem Int Ed. 2009;48(12):2144–2147. DOI:10.1002/anie.200804762 19204969

[381] Varanakkottu SN, George SD, Baier T, et al. Particle manipulation based on optically controlled free surface hydrodynamics. Angew Chem Int Ed. 2013;52(28):7291–7295. DOI:10.1002/anie.201302111 23729315

[382] Kavokine N, Anyfantakis M, Morel M, et al. Light-driven transport of a liquid marble with and against surface flows. Angew Chem Int Ed. 2016;55(37):11183–11187. DOI:10.1002/anie.201603639 27381297

[383] Rossegger E, Hennen D, Griesser T, et al. Directed motion of water droplets on multi-gradient photopolymer surfaces. Polym Chem. 2019;10(15):1882–1893. DOI:10.1039/C9PY00123A

[384] Xia J, Zhao P, Zheng K, et al. Surface modification based on diselenide dynamic chemistry: towards liquid motion and surface bioconjugation. Angew Chem. 2018;310036:552–556. 10.1002/anie.201810588 30457188

[385] Berná J, Leigh DA, Lubomska M, et al. Macroscopic transport by synthetic molecular machines. Nat Mater. 2005;4(9):704–710. DOI:10.1038/nmat1455 16127455

[386] Monteleone FV, Caputo G, Canale C, et al. Light-controlled directional liquid drop movement on TiO_2_ nanorods-based nanocomposite photopatterns. Langmuir. 2010;26(23):18557–18563. DOI:10.1021/la1026398 21028854

[387] Rossegger E, Nees D, Turisser S, et al. Photo-switching of surface wettability on micropatterned photopolymers for fast transport of water droplets over a long-distance. Polym Chem. 2020;11(18):3125–3135. DOI:10.1039/D0PY00263A

[388] Xu B, Zhu C, Qin L, et al. Light-directed liquid manipulation in flexible bilayer microtubes. Small. 2019;15(24):1901847. DOI:10.1002/smll.201901847 31062929

[389] Liu Q, Yu G, Zhu C, et al. An Integrated droplet manipulation platform with photodeformable microfluidic channels. Small Methods. 2021;5(12):2100969. DOI:10.1002/smtd.202100969 34928016

[390] Ji S, Cao W, Yu Y, et al. Dynamic diselenide bonds: exchange reaction induced by visible light without catalysis. Angew Chem Int Ed. 2014;53(26):6781–6785. DOI:10.1002/anie.201403442 24842614

[391] Ji S, Xia J, Xu H. Dynamic chemistry of selenium: Se–N and Se–Se dynamic covalent bonds in polymeric systems. ACS Macro Lett. 2016;5(1):78–82. 35668594 10.1021/acsmacrolett.5b00849

[392] Xia J, Ji S, Xu H. Diselenide covalent chemistry at the interface: stabilizing an asymmetric diselenide-containing polymer *via* micelle formation. Polym Chem. 2016;7(44):6708–6713.

[393] Voloshchenko D, Khyzhnyak A, Reznikov Y, et al. Control of an easy axis on a nematic-polymer interface by a light action to a nematic bulk. Jpn J Appl Phys. 1995;34(Part 1, No. 2A):566. DOI:10.1143/JJAP.34.566

[394] Slussarenko S, Francescangeli O, Simoni F, et al. High resolution polarization gratings in liquid crystals. Appl Phys Lett. 1997;71(25):3613–3615. DOI:10.1063/1.120457

[395] Simoni F, Francescangeli O, Reznikov Y, et al. Dye-doped liquid crystals as high-resolution recording media: errata. Opt Lett. 1997;22(12):937. DOI:10.1364/OL.22.000937 18185712

[396] Francescangeli O, Slussarenko S, Simoni F, et al. Light-induced surface sliding of the nematic director in liquid crystals. Phys Rev Lett. 1999;82(9):1855–1858. DOI:10.1103/PhysRevLett.82.1855

[397] Simoni F, Francescangeli O. Effects of light on molecular orientation of liquid crystals. J Phys Condens Matter. 1999;11(41):439–487.

[398] Motevalli B, Taherifar N, Wu B, et al. A density functional theory computational study of adsorption of di-meta-cyano azobenzene molecules on Si (111) surfaces. Appl Surf Sci. 2017;422:557–565.

[399] Komitov L, Ruslim C, Matsuzawa Y, et al. Photoinduced anchoring transitions in a nematic doped with azo dyes. Liq Cryst. 2000;27(8):1011–1016. DOI:10.1080/02678290050080733

[400] Komitov L, Ichimura K, Strigazzi A. Light-induced anchoring transition in a 4,4’-disubstituted azobenzene nematic liquid crystal. Liq Cryst. 2000;27(1):51–55.

[401] Ruslim C, Komitov L, Matsuzawa Y, et al. Effect of conformations of *trans*- and *cis*-azobenzenes on photoinduced anchoring transitions in a nematic liquid crystal. Japanese J Appl Physics. 2000;39(Part 2, No. 2A):L104–106. Part 2 Lett:

[402] Lee C-H, Chen C-H, Kao C-L, et al. Photo and electrical tunable effects in photonic liquid crystal fiber. Opt Express. 2010;18(3):2814. DOI:10.1364/OE.18.002814 20174110

[403] Ouskova E, Vapaavuori J, Kaivola M. Self-orienting liquid crystal doped with polymer-azo-dye complex. Opt Mater Express. 2011;1(8):1463.

[404] Yang K-Y, Lee W. Voltage-assisted photoaligning effect of an azo dye doped in a liquid crystal with negative dielectric anisotropy. Opt Express. 2010;18(19):19914. 20940882 10.1364/OE.18.019914

[405] Shen Y, Xu Y-C, Ge Y-H, et al. Photoalignment of dye-doped cholesteric liquid crystals for electrically tunable patterns with fingerprint textures. Opt Express. 2018;26(2):1422. DOI:10.1364/OE.26.001422 29402016

[406] Lee CR, Fu TL, Cheng KT, et al. Surface-assisted photoalignment in dye-doped liquid-crystal films. Phys Rev E. 2004;69(3):031704. DOI:10.1103/PhysRevE.69.031704 15089307

[407] Chen CH, Lee CH, Lin TH. Loss-reduced photonic liquid-crystal fiber by using photoalignment method. Appl Opt. 2010;49(26):4846–4850. 20830171 10.1364/AO.49.004846

[408] Fuh A-G, Liao C-C, Hsu K-C, et al. Laser-induced reorientation effect and ripple structure in dye-doped liquid-crystal films. Opt Lett. 2003;28(14):1179. DOI:10.1364/OL.28.001179 12885013

[409] Lee CR, Mo TS, Cheng KT, et al. Electrically switchable and thermally erasable biphotonic holographic gratings in dye-doped liquid crystal films. Appl Phys Lett. 2003;83(21):4285–4287. DOI:10.1063/1.1629374

[410] Chen WZ, Tsai YT, Lin TH. Photoalignment effect in a liquid-crystal film doped with nanoparticles and azo-dye. Appl Phys Lett. 2009;94(20):201114.

[411] Fuh AYG, Chen JC, Huang SY, et al. Binary liquid crystal alignments based on photoalignment in azo dye-doped liquid crystals and their application. Appl Phys Lett. 2010;96(5):2008–2011. DOI:10.1063/1.3299268

[412] Wu WY, Fuh AYG. Rewritable liquid crystal gratings fabricated using photoalignment effect in dye-doped poly(vinyl alcohol) film. Jpn J Appl Phys. 2007;46(10A):6761–6766.

[413] Khoo IC, Wood M, Shih MY, et al. Extremely nonlinear photosensitive liquid crystals for image sensing and sensor protection. Opt Express. 1999;4(11):432. DOI:10.1364/OE.4.000432 19396300

[414] Lin TH, Jau HC, Hung SY, et al. Photoaddressable bistable reflective liquid crystal display. Appl Phys Lett. 2006;89(2):021116. DOI:10.1063/1.2219406

[415] Lin L-C, Jau H-C, Lin T-H, et al. Highly efficient and polarization-independent fresnel lens based on dye-doped liquid crystal. Opt Express. 2007;15(6):2900–2906. DOI:10.1364/OE.15.002900 19532525

[416] Khoo IC, Shih M-Y, Wood MV, et al. Dye-doped photorefractive liquid crystals for dynamic and storage holographic grating formation and spatial light modulation. Proc IEEE. 1999;87(11):1897–1911. DOI:10.1109/5.796353

[417] Wang C-T, Jau H-C, Lin T-H. Optically controllable bistable reflective liquid crystal display. Opt Lett. 2012;37(12):2370. 22739911 10.1364/OL.37.002370

[418] Huang SY, Wu ST, Fuh AYG. Optically switchable twist nematic grating based on a dye-doped liquid crystal film. Appl Phys Lett. 2006;88(4):041104.

[419] Wu W-Y, Mo T-S, Fuh A-G. Polarization characteristics of diffracted beams from twisted nematic gratings fabricated by the photoalignment effect in dye-doped liquid-crystal films. J Opt Soc Am B. 2006;23(9):1737.

[420] Fuh A-G, Chen J-C, Cheng K-T, et al. Polarization-independent and electrically tunable liquid-crystal fresnel lenses based on photoalignment in dye-doped liquid crystals. J Soc Inf Disp. 2010;18(8):572. DOI:10.1889/JSID18.8.572

[421] Huang Y, Ko S, Chu S, et al. High-efficiency fresnel lens fabricated by axially symmetric photoalignment method. Appl Opt. 2012;51(32):7739–7744. DOI:10.1364/AO.51.007739 23142883

[422] Okabe Y, Ogawa M. Photoinduced adsorption of spiropyran into mesoporous silicas as photomerocyanine. RSC Adv. 2015;5(123):101789–101793.

[423] Yamaguchi T, Maity A, Polshettiwar V, et al. Photochromism of a spiropyran in the presence of a dendritic fibrous nanosilica; simultaneous photochemical reaction and adsorption. J Phys Chem A. 2017;121(42):8080–8085. DOI:10.1021/acs.jpca.7b08466 28972757

[424] Yamaguchi T, Leelaphattharaphan NN, Shin H, et al. Acceleration of photochromism and negative photochromism by the interactions with mesoporous silicas. Photochem Photobiol Sci. 2019;18(7):1742–1749. DOI:10.1039/C9PP00081J 31093626

[425] Yamaguchi T, Ogawa M. Photochromism of a spiropyran in the presence of a synthetic hectorite. Chem Lett. 2018;47(2):189–191.

[426] Leelaphattharaphan NN, Deepracha SB, Yamaguchi T, et al. Adsorption of a spiropyran on a layered clay mineral. IOP Conf Ser Earth Environ Sci. 2022;950(1):012041. DOI:10.1088/1755-1315/950/1/012041

[427] Colaço M, Carletta A, Van Gysel M, et al. Direct access by mechanochemistry or sonochemistry to protonated merocyanines: components of a four-state molecular switch. ChemistryOpen. 2018;7(7):520–526. DOI:10.1002/open.201800082 30003006 PMC6031862

[428] Wang Y, Li B, Zhang L, et al. Targeted delivery system based on magnetic mesoporous silica nanocomposites with light-controlled release character. ACS Appl Mater Interfaces. 2013;5(1):11–15. DOI:10.1021/am302492e 23245393

[429] Angelos S, Choi E, Vögtle F, et al. Photo-driven expulsion of molecules from mesostructured silica nanoparticles. J Phys Chem C. 2007;111(18):6589–6592. DOI:10.1021/jp070721l

[430] Lu J, Choi E, Tamanoi F, et al. Light-activated nanoimpeller-controlled drug release in cancer cells. Small. 2008;4(4):421–426. DOI:10.1002/smll.200700903 18383576 PMC2712492

[431] Liu J, Bu W, Pan L, et al. NIR-triggered anticancer drug delivery by upconverting nanoparticles with integrated azobenzene-modified mesoporous silica. Angew Chem Int Ed. 2013;52(16):4375–4379. DOI:10.1002/anie.201300183 23495013

[432] Duan Y, Wang Y, Li X, et al. Light-triggered nitric oxide (NO) release from photoresponsive polymersomes for corneal wound healing. Chem Sci. 2020;11(1):186–194. DOI:10.1039/C9SC04039K 32110370 PMC7012058

[433] Nehls EM, Rosales AM, Anseth KS. Enhanced user-control of small molecule drug release from a poly(ethylene glycol) hydrogel via azobenzene/cyclodextrin complex tethers. J Mater Chem B. 2016;4(6):1035–1039. 27127630 10.1039/C5TB02004B PMC4843523

[434] Huang Q, Bao C, Ji W, et al. Photocleavable coumarin crosslinkers based polystyrene microgels: phototriggered swelling and release. J Mater Chem. 2012;22(35):18275–18282. DOI:10.1039/c2jm33789d

[435] Wang Z, Johns VK, Liao Y. Controlled release of fragrant molecules with visible light. Chem - A Eur J. 2014;20(45):14637–14640. 10.1002/chem.201404203 25284277

[436] Jin Q, Mitschang F, Agarwal S. Biocompatible drug delivery system for photo-triggered controlled release of 5-fluorouracil. Biomacromolecules. 2011;12(10):3684–3691. 21863834 10.1021/bm2009125

[437] Wang H, Miao W, Wang F, et al. A self-assembled coumarin-anchored dendrimer for efficient gene delivery and light-responsive drug delivery. Biomacromolecules. 2018;19(6):2194–2201. DOI:10.1021/acs.biomac.8b00246 29684275

[438] Tan X, Li BB, Lu X, et al. Light-triggered, self-immolative nucleic acid-drug nanostructures. J Am Chem Soc. 2015;137(19):6112–6115. DOI:10.1021/jacs.5b00795 25924099

[439] Bozuyuk U, Yasa O, Yasa IC, et al. Light-triggered drug release from 3D-printed magnetic chitosan microswimmers. ACS Nano. 2018;12(9):9617–9625. DOI:10.1021/acsnano.8b05997 30203963

[440] Zang C, Wang H, Li T, et al. A light-responsive, self-immolative linker for controlled drug delivery *via* peptide- and protein-drug conjugates. Chem Sci. 2019;10(39):8973–8980. DOI:10.1039/C9SC03016F 31762977 PMC6857671

[441] Pei P, Sun C, Tao W, et al. ROS-sensitive thioketal-linked polyphosphoester-doxorubicin conjugate for precise phototriggered locoregional chemotherapy. Biomaterials. 2019;188:74–82. 30336287 10.1016/j.biomaterials.2018.10.010

[442] Cui D, Huang J, Zhen X, et al. A semiconducting polymer nano-prodrug for hypoxia-activated photodynamic cancer therapy. Angew Chem Int Ed. 2019;58(18):5920–5924. DOI:10.1002/anie.201814730 30793456

[443] Wu C, Chen C, Lai J, et al. Molecule-scale controlled-release system based on light-responsive silica nanoparticles. Chem Commun. 2008;23(23):2662–2664. DOI:10.1039/b804886j 18535700

[444] Pelliccioli AP, Wirz J. Photoremovable protecting groups: reaction mechanisms and applications. Photochem Photobiol Sci. 2002;1(7):441–458. 12659154 10.1039/b200777k

[445] Peng K, Tomatsu I, Kros A. Light controlled protein release from a supramolecular hydrogel. Chem Commun. 2010;46(23):4094–4096. 10.1039/c002565h 20464018

[446] Pianowski ZL, Karcher J, Schneider K. Photoresponsive self-healing supramolecular hydrogels for light-induced release of DNA and doxorubicin. Chem Commun. 2016;52(15):3143–3146. 10.1039/c5cc09633b 26804160

[447] Liu XM, Yang B, Wang YL, et al. Photoisomerisable cholesterol derivatives as photo-trigger of liposomes: effect of lipid polarity, temperature, incorporation ratio, and cholesterol. Biochim Biophys Acta - Biomembr. 2005;1720(1–2):28–34. DOI:10.1016/j.bbamem.2005.10.016 16368070

[448] Liu YC, Ny ALML, Schmidt J, et al. Photo-assisted gene delivery using light-responsive catanionic vesicles. Langmuir. 2009;25(10):5713–5724. DOI:10.1021/la803588d 19435291

[449] Hu XY, Jia K, Cao Y, et al. Dual photo- and pH-responsive supramolecular nanocarriers based on water-soluble pillar[6]arene and different azobenzene derivatives for intracellular anticancer drug delivery. Chem - A Eur J. 2015;21(3):1208–1220. DOI:10.1002/chem.201405095 25370941

[450] Liu H, Liu Y, Shang Y, et al. Molecular dynamics simulation for drug delivery in azobenzene-containing membranes. Mol Simul. 2020;46(4):300–307. DOI:10.1080/08927022.2019.1699655

[451] Ishihara K, Negishi N, Shinohara I. Photoregulated binding ability of azoaromatic polymer for surfactant. J Polym Sci. 1981;19(12):3039–3046.

[452] Ishihara K, Negishi N, Shinohara I. Photocontrolled adsorption chromatography for lysozyme using azoaromatic polymer. J Appl Polym Sci. 1982;27(6):1897–1902.

[453] Kanj AB, Bürck J, Grosjean S, et al. Switching the enantioselectivity of nanoporous host materials by light. Chem Commun. 2019;55(60):8776–8779. DOI:10.1039/C9CC02849H 31099346

[454] Ishihara K, Kato S, Shinohara I. Separation of proteins by polymeric adsorbents containing azobenzene moiety as a ligand. J Appl Polym Sci. 1982;27(11):4273–4282.

[455] Wang Z, Grosjean S, Bräse S, et al. Photoswitchable adsorption in metal-organic frameworks based on polar guest-host interactions. Chemphyschem. 2015;16:3779–3783. 26455589 10.1002/cphc.201500829

[456] Puntoriero F, Ceroni P, Balzani V, et al. Photoswitchable dendritic hosts: a dendrimer with peripheral azobenzene groups. J Am Chem Soc. 2007;129(35):10714–10719. DOI:10.1021/ja070636r 17696531

[457] Park J, Sun LB, Chen YP, et al. Azobenzene-functionalized metal-organic polyhedra for the optically responsive capture and release of guest molecules. Angew Chem Int Ed. 2014;53(23):5842–5846. DOI:10.1002/anie.201310211 24803325

[458] Nakahara Y, Okazaki Y, Kimura K. Self-assembling phenomena of benzo-18-crown-6/crowned-spirobenzopyran copolymers in aqueous solution and controlled release of entrapped organic dyes using external stimuli. Soft Matter. 2012;8:3192–3199.

[459] Wang X, Hu J, Liu G, et al. Reversibly switching bilayer permeability and release modules of photochromic polymersomes stabilized by cooperative noncovalent interactions. J Am Chem Soc. 2015;137(48):15262–15275. DOI:10.1021/jacs.5b10127 26583385

[460] Chen S, Gao Y, Cao Z, et al. Nanocomposites of spiropyran-functionalized polymers and upconversion nanoparticles for controlled release stimulated by near-infrared light and pH. Macromolecules. 2016;49(19):7490–7496. DOI:10.1021/acs.macromol.6b01760

[461] Müller K, Wadhwa J, Singh Malhi J, et al. Photoswitchable nanoporous films by loading azobenzene in metal–organic frameworks of type HKUST-1. Chem Commun. 2017;53(57):8070–8073. DOI:10.1039/C7CC00961E 28676871

[462] Alvaro M, Benitez M, Das D, et al. Reversible porosity changes in photoresponsive azobenzene-containing periodic mesoporous silicas. Chem Mater. 2005;17:4958–4964.

[463] Brown JW, Henderson BL, Kiesz MD, et al. Photophysical pore control in an azobenzene-containing metal-organic framework. Chem Sci. 2013;4:2858–2864.

[464] Wang Z, Heinke L, Jelic J, et al. Photoswitching in nanoporous, crystalline solids: an experimental and theoretical study for azobenzene linkers incorporated in MOFs. Phys Chem Chem Phys. 2015;17(22):14582–14587. DOI:10.1039/C5CP01372K 25966648

[465] Müller K, Knebel A, Zhao F, et al. Switching thin films of azobenzene-containing metal–organic frameworks with visible light. Chem Eur J. 2017;23(23):5434–5438. DOI:10.1002/chem.201700989 28370503

[466] Zhang L, Wang LL, Le GL, et al. Coumarin-modified microporous-mesoporous Zn-MOF-74 showing ultra-high uptake capacity and photo-switched storage/release of U^VI^ ions. J Hazard Mater. 2016;311:30–36. 26954473 10.1016/j.jhazmat.2016.01.082

[467] Mal NK, Fujiwara M, Tanaka Y. Photocontrolled reversible release of guest molecules from coumarin-modified mesoporous silica. Nature. 2003;421(6921):350–353. 12540896 10.1038/nature01362

[468] Mal NK, Fujiwara M, Tanaka Y, et al. Photo-switched storage and release of guest molecules in the pore void of coumarin-modified MCM-41. Chem Mater. 2003;15(17):3385–3394. DOI:10.1021/cm0343296

[469] Chen L, Wang W, Su B, et al. A light-responsive release platform by controlling the wetting behavior of hydrophobic surface. ACS Nano. 2014;8(1):744–751. DOI:10.1021/nn405398d 24383581

[470] Xing Q, Li N, Chen D, et al. Light-responsive amphiphilic copolymer coated nanoparticles as nanocarriers and real-time monitors for controlled drug release. J Mater Chem B. 2014;2(9):1182–1189. DOI:10.1039/c3tb21269f 32261354

[471] Heinke L, Cakici M, Dommaschk M, et al. Photoswitching in two-component surface-mounted metal–organic frameworks: optically triggered release from a molecular container. ACS Nano. 2014;8(2):1463–1467. DOI:10.1021/nn405469g 24400960

[472] Meng X, Gui B, Yuan D, et al. Mechanized azobenzene-functionalized zirconium metal-organic framework for on-command cargo release. Sci Adv. 2016;2(8):2–8. DOI:10.1126/sciadv.1600480 PMC4972467 27493996

[473] Mutruc D, Goulet-Hanssens A, Fairman S, et al. Modulating guest uptake in core–shell MOFs with visible light. Angew Chem Int Ed. 2019;58(37):12862–12867. DOI:10.1002/anie.201906606 31183909

[474] Ou R, Zhang H, Zhao C, et al. Photoresponsive styrylpyrene-modified MOFs for gated loading and release of cargo molecules. Chem Mater. 2020;32(24):10621–10627. DOI:10.1021/acs.chemmater.0c03726

[475] Zhu Y, Fujiwara M. Installing dynamic molecular photomechanics in mesopores: a multifunctional controlled-release nanosystem. Angew Chem Int Ed. 2007;46(13):2241–2244. 10.1002/anie.200604850 17295377

[476] Ji W, Li N, Chen D, et al. Coumarin-containing photo-responsive nanocomposites for NIR light-triggered controlled drug release *via* a two-photon process. J Mater Chem B. 2013;1(43):5942–5949. DOI:10.1039/c3tb21206h 32261061

[477] Yuan Q, Zhang Y, Chen T, et al. Photon-manipulated drug release from a mesoporous nanocontainer controlled by azobenzene-modified nucleic acid. ACS Nano. 2012;6(7):6337–6344. DOI:10.1021/nn3018365 22670595 PMC3407578

[478] Yan H, Teh C, Sreejith S, et al. Functional mesoporous silica nanoparticles for photothermal-controlled drug delivery in vivo. Angew Chem Int Ed. 2012;51(33):8373–8377. DOI:10.1002/anie.201203993 22777795

[479] Wang D, Wu S. Red-light-responsive supramolecular valves for photocontrolled drug release from mesoporous nanoparticles. Langmuir. 2016;32(2):632–636. 26700509 10.1021/acs.langmuir.5b04399

[480] Li M, Yan H, Teh C, et al. NIR-triggered drug release from switchable rotaxane-functionalized silica-covered Au nanorods. Chem Commun. 2014;50(68):9745–9748. DOI:10.1039/C4CC02966F 25019373

[481] Cheng L, Jiang Y, Yan N, et al. Smart adsorbents with photoregulated molecular gates for both selective adsorption and efficient regeneration. ACS Appl Mater Interfaces. 2016;8(35):23404–23411. DOI:10.1021/acsami.6b07853 27559985

[482] Zhang Z, Balogh D, Wang F, et al. Light-induced and redox-triggered uptake and release of substrates to and from mesoporous SiO_2_ nanoparticles. J Mater Chem B. 2013;1(25):3159. DOI:10.1039/c3tb20292e 32260916

[483] Aznar E, Oroval M, Pascual L, et al. Gated materials for on-command release of guest molecules. Chem Rev. 2016;116(2):561–718. DOI:10.1021/acs.chemrev.5b00456 26730615

[484] Wu MX, Yang YW. Metal-organic framework (MOF)-based drug/cargo delivery and cancer therapy. Adv Mater. 2017;29(23):1606134. 10.1002/adma.201606134 28370555

[485] Nakata S, Miyaji T, Matsuda Y, et al. Mode switching of a self-propelled camphor disk sensitive to the photoisomerization of a molecular layer on water. Langmuir. 2014;30(25):7353–7357. DOI:10.1021/la5016803 24901870

[486] Nakata S, Nasu K, Irie Y, et al. Self-propelled motion of a camphor disk on a photosensitive amphiphilic molecular layer. Langmuir. 2019;35(12):4233–4237. DOI:10.1021/acs.langmuir.8b04285 30807697

[487] Matsuda Y, Suematsu NJ, Nakata S. Photo-sensitive self-motion of a BQ disk. Phys Chem Chem Phys. 2012;14:5988–5991. 22446889 10.1039/c2cp40306d

[488] Norikane Y, Tanaka S, Uchida E. Azobenzene crystals swim on water surface triggered by light. Cryst Eng Comm. 2016;18(38):7225–7228.

[489] Yucknovsky A, Rich BB, Westfried A, et al. Self-propulsion of droplets via light-stimuli rapid control of their surface tension. Adv Mater Interfaces. 2021;8(22):2100751. DOI:10.1002/admi.202100751

[490] Liu CW, Hsu CP, Yeh JA, et al. Light-actuated water droplet motions on ZnO nanorods. Microsyst Technol. 2013;19(2):245–251. DOI:10.1007/s00542-012-1562-5

[491] Li W, Lei Y, Chen R, et al. Light-caused droplet bouncing from a cavity trap-assisted superhydrophobic surface. Langmuir. 2020;36(37):11068–11078. DOI:10.1021/acs.langmuir.0c02062 32847362

[492] Wu Z, Lin X, Wu Y, et al. Near-infrared light-triggered “on/off” motion of polymer multilayer rockets. ACS Nano. 2014;8(6):6097–6105. DOI:10.1021/nn501407r 24806430

[493] Xuan M, Wu Z, Shao J, et al. Near infrared light-powered Janus mesoporous silica nanoparticlemotors. J Am Chem Soc. 2016;138(20):6492–6497. DOI:10.1021/jacs.6b00902 27152728

[494] Uda M, Kawashima H, Mayama H, et al. Locomotion of a nonaqueous liquid marble induced by near-infrared-light irradiation. Langmuir. 2021;37(14):4172–4182. DOI:10.1021/acs.langmuir.1c00041 33788574

[495] Gao C, Wang L, Lin Y, et al. Droplets manipulated on photothermal organogel surfaces. Adv Funct Mater. 2018;28(35):1803072. DOI:10.1002/adfm.201803072

[496] Dai B, Wang J, Xiong Z, et al. Programmable artificial phototactic microswimmer. Nat Nanotechnol. 2016;11(12):1087–1092. DOI:10.1038/nnano.2016.187 27749832

[497] Wu S, Zhou L, Chen C, et al. Photothermal actuation of diverse liquids on an Fe_3_O_4_-doped slippery surface for electric switching and cell culture. Langmuir. 2019;35(43):13915–13922. DOI:10.1021/acs.langmuir.9b02068 31566979

[498] Yilmaz M, Kuloglu HB, Erdogan H, et al. Light-driven unidirectional liquid motion on anisotropic gold nanorod arrays. Adv Mater Interfaces. 2015;2(12):1500226. DOI:10.1002/admi.201500226

[499] Chen C, Mou F, Xu L, et al. Light-steered isotropic semiconductor micromotors. Adv Mater. 2017;29(3):1603374. DOI:10.1002/adma.201603374 27748536

[500] Wu Y, Si T, Lin X, et al. Near infrared-modulated propulsion of catalytic janus polymer multilayer capsule motors. Chem Commun. 2015;51(3):511–514. DOI:10.1039/C4CC07182D 25409875

[501] Geng H, Zhou K, Zhou J, et al. Sunlight-driven water transport *via* a reconfigurable pump. Angew Chem Int Ed. 2018;57(47):15435–15440. DOI:10.1002/anie.201808835 30311339

[502] Ge F, Yang R, Tong X, et al. A multifunctional dye-doped liquid crystal polymer actuator: light-guided transportation, turning in locomotion, and autonomous motion. Angew Chem Int Ed. 2018;57(36):11758–11763. DOI:10.1002/anie.201807495 30025194

[503] Lv X, Wang W, Yu H. A bioinspired photothermal pneumatic device enabling optical manipulation of microfluid toward precise control of microreactions. Adv Eng Mater. 2019;21(12):1900977.

[504] Ren Y, Qi H, Chen Q, et al. Optofluidic control using light illuminated plasmonic nanostructure as microvalve. Int J Heat Mass Transf. 2019;133:1019–1025.

[505] Yu Y, Nakano M, Ikeda T. Directed bending of a polymer film by light. Nature. 2003;425(6954):145. 12968169 10.1038/425145a

[506] Miyasaka H, Matsuda K, Abe J, et al. Photosynergetic responses in molecules and molecular aggregates. 1st ed. Miyasaka H, Matsuda K Abe J, editors. Singapore: Springer Singapore; 2020.

[507] Tang X, Wang L. Loss-free photo-manipulation of droplets by pyroelectro-trapping on superhydrophobic surfaces. ACS Nano. 2018;12(9):8994–9004. 30125483 10.1021/acsnano.8b02470

[508] Li W, Tang X, Wang L. Photopyroelectric microfluidics. Sci Adv. 2020;6(38):1693. 10.1126/sciadv.abc1693 PMC7494354 32938667

